# Heterogeneity and predictors of the effects of AI assistance on radiologists

**DOI:** 10.1038/s41591-024-02850-w

**Published:** 2024-03-19

**Authors:** Feiyang Yu, Alex Moehring, Oishi Banerjee, Tobias Salz, Nikhil Agarwal, Pranav Rajpurkar

**Affiliations:** 1 Department of Biomedical Informatics, Harvard Medical School, Boston, MA USA; 2 Department of Computer Science, Stanford University, Stanford, CA USA; 3 Sloan School of Management, Massachusetts Institute of Technology, Cambridge, MA USA; 4 Department of Economics, Massachusetts Institute of Technology, Cambridge, MA USA

**Keywords:** Machine learning, Radiography

## Abstract

The integration of artificial intelligence (AI) in medical image interpretation requires effective collaboration between clinicians and AI algorithms. Although previous studies demonstrated the potential of AI assistance in improving overall clinician performance, the individual impact on clinicians remains unclear. This large-scale study examined the heterogeneous effects of AI assistance on 140 radiologists across 15 chest X-ray diagnostic tasks and identified predictors of these effects. Surprisingly, conventional experience-based factors, such as years of experience, subspecialty and familiarity with AI tools, fail to reliably predict the impact of AI assistance. Additionally, lower-performing radiologists do not consistently benefit more from AI assistance, challenging prevailing assumptions. Instead, we found that the occurrence of AI errors strongly influences treatment outcomes, with inaccurate AI predictions adversely affecting radiologist performance on the aggregate of all pathologies and on half of the individual pathologies investigated. Our findings highlight the importance of personalized approaches to clinician–AI collaboration and the importance of accurate AI models. By understanding the factors that shape the effectiveness of AI assistance, this study provides valuable insights for targeted implementation of AI, enabling maximum benefits for individual clinicians in clinical practice.

## Abstract (web summary)

A large-scale study examined the heterogeneous effects of artificial intelligence (AI) assistance on 140 radiologists across 15 chest X-ray diagnostic tasks and found that conventional experience-based factors, such as years of experience, subspecialty and familiarity with AI tools, fail to reliably predict the impact of AI assistance.

## Main

The integration of artificial intelligence (AI) into medical image interpretation has shown great potential for improving diagnostic accuracy and efficiency^1–9^. Collaborative setups, where AI systems assist clinicians in decision-making, have emerged as practical approaches to harness the benefits of AI while leveraging clinician expertise^10–13^. However, to optimize the implementation of AI in clinical practice, it is crucial to have a comprehensive understanding of the heterogeneity—the diverse and individualized effects—of AI assistance on clinicians. Clinicians possess varying levels of expertise, experience and decision-making styles, and ensuring that AI support accommodates this heterogeneity is essential for targeted implementation and maximizing the positive impact on patient care.

Previous studies on clinician–AI collaboration predominantly focused on analyzing groups of clinicians as a whole, overlooking the variations in how AI affects individual clinicians^14–20^. Although some studies explored the heterogeneity of AI effects based on factors such as a radiologist’s seniority^21–23^, task expertise^24^ and experience level^25–28^, these studies have certain limitations. They often measure changes in predictions rather than changes in prediction accuracy, and they tend to neglect potential predictors, such as experience with AI tools. Additionally, although some research considered indirect measures of diagnostic skill, such as years of experience, there remains a limited understanding of whether direct measures of clinicians’ diagnostic skill can accurately predict the effects of AI assistance. Therefore, conducting a comprehensive investigation into the heterogeneous effects of AI is crucial for determining which clinicians should receive AI assistance in real-world healthcare settings.

In the present study, we investigated the predictors of heterogeneous treatment effects of AI assistance in radiology, where treatment effect refers to the change in diagnostic performance of radiologists from without to with AI assistance. To achieve this, we examined a large-scale diagnostic study that measured the performance of 140 radiologists with and without AI assistance on 15 chest X-ray diagnosis tasks. Participating radiologists received onboarding training on the assistive AI system before starting the experiment and were shown example AI predictions from the same AI model used in the experiment, which would help them calibrate their interpretation of AI predictions and inform their incorporation of AI. Our analysis focuses on the influence of experience-based predictors, direct measures of diagnostic skill and AI error on the outcome of treatment effects and examines this influence in terms of both calibration performance and discrimination performance. We found substantial heterogeneity in the treatment effects of AI assistance among radiologists. Moreover, our findings reveal that experience-based characteristics and direct measures of diagnostic skill prove inadequate in predicting the treatment effect of AI assistance on radiologists. Additionally, we highlight the influential role of AI error on the treatment effect. Lower absolute AI error leads to a greater treatment effect on all pathologies aggregated and on half of the individual pathologies investigated, and the direction of AI error also impacts the treatment effect outcome. By uncovering the heterogeneity of AI effects and identifying predictors of the treatment effect, our study offers valuable insights for the targeted implementation of AI assistance in clinical practice. Comprehending the factors contributing to the heterogeneity of AI effects is vital for the development of tailored strategies to optimize clinician–AI collaboration, to guide resource allocation and training efforts and to foster trust and acceptance among clinicians.

## Results

### Heterogeneous treatment effects of AI assistance

We analyzed data collected using a diagnostic study involving 140 radiologists, 324 patient cases and 15 pathologies with corresponding AI predictions from two study designs: one with repeated measurements on the same case and one without^29^. The non-repeated-measure design involved 107 radiologists who each reviewed a total of 60 patient cases, with 30 cases assessed without AI assistance and 30 cases assessed with AI assistance. For each set of 30 patient cases, radiologists examined half without clinical histories and half with clinical histories. The repeated-measure design included 33 radiologists who evaluated 60 patient cases under four conditions: with AI assistance and clinical histories, with AI assistance without clinical histories, without AI assistance with clinical histories and without either AI assistance or clinical histories. In our analysis, we combined data from the clinical history conditions and investigated the heterogeneous treatment effect of AI assistance.

In this study, calibration performance was measured by absolute error. Absolute error was defined as the absolute difference between the radiologist-predicted probability and the ground truth probability on a 0–100 scale. Treatment effect was defined as the improvement in absolute error, specifically the difference between the absolute error of a radiologist when unassisted by AI (unassisted error) and the absolute error when assisted by AI (assisted error), unless otherwise specified. Absolute error was the primary metric of analysis; thus, references to performance and treatment effects are based on this metric by default. Discrimination performance was measured by area under the receiver operating characteristic (ROC) curve, where the ground truth labels were computed by thresholding the continuous ground truth probabilities at 50. Treatment effect on AUROC was defined as the improvement in AUROC, specifically the difference between the AUROC of a radiologist when assisted by AI (assisted AUROC) and the AUROC when unassisted by AI (unassisted AUROC).

**Extended Data Fig. 1:**
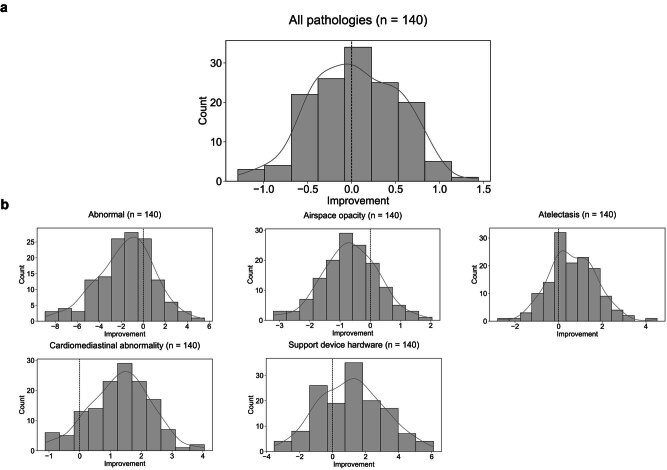
Individual heterogeneity in treatment effects. **a**, **b**, Individual heterogeneity in treatment effects of 140 radiologists as determined using the empirical Bayes method on (**a**) all pathologies aggregated and (**b**) high-prevalence pathology labels (pathology labels with greater than 10% prevalence). The curve is the kernel density estimate (KDE).

**Extended Data Fig. 2:**
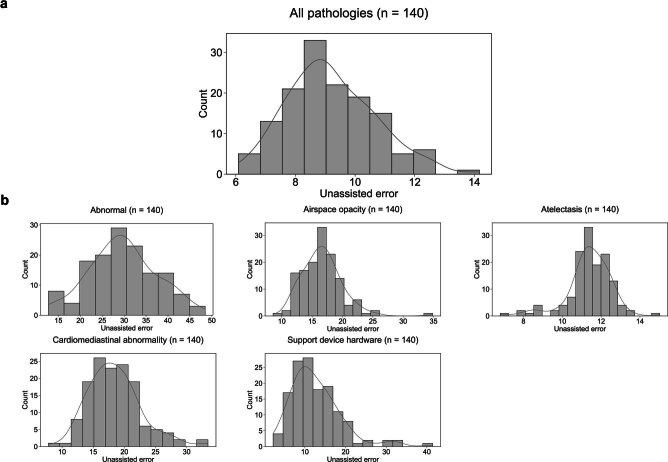
Individual heterogeneity in unassisted error. **a**, **b**, Individual heterogeneity in unassisted error of 140 radiologists as determined using the empirical Bayes method on (**a**) all pathologies aggregated and (**b**) high-prevalence pathology labels (pathology labels with greater than 10% prevalence). The curve is the kernel density estimate (KDE).

Our findings revealed substantial heterogeneity in the treatment effects of AI assistance among different radiologists (Extended Data Fig. 1 and Supplementary Table 1). When measuring AI’s treatment effect as the improvement in absolute error across all pathologies, we observed a range of treatment effects from −1.295 to 1.440 (interquartile range (IQR), 0.797). Notably, for high-prevalence pathology labels (pathology labels with prevalence greater than 10% in the dataset), the largest range of treatment effects extended from −8.914 to 5.563 (IQR, 3.245) for detecting whether chest X-rays are abnormal. In comparison, when examining the distribution of radiologists’ unassisted error (Extended Data Fig. 2), we observed an average range of unassisted error from 6.083 to 14.175 (IQR, 1.951) across pathologies. The significant heterogeneity in treatment effects indicates that the impact of treatment effects ranging from −1.295 to 1.440 (IQR, 0.797) could substantially influence the absolute performance and relative performance of radiologists compared to their peers. Furthermore, the heterogeneity in treatment effects on high-prevalence pathology labels remained substantial when compared to radiologists’ unassisted error.

**Extended Data Fig. 3:**
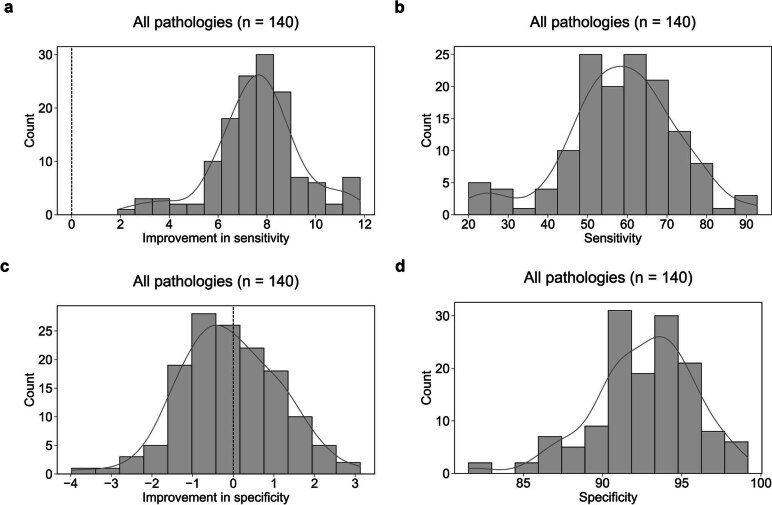
Individual heterogeneity in treatment effects on sensitivity, sensitivities, treatment effects on specificity, and specificities. **a-b**, Individual heterogeneity in (**a**) improvement in sensitivities, (**b**) sensitivity, (**c**) improvement in specificity, and (**d**) specificity of 140 radiologists as determined using the empirical Bayes method on all pathologies aggregated. The curve is the kernel density estimate (KDE).

Additionally, we found substantial heterogeneity in treatment effects on sensitivity and specificity. The range of treatment effects on radiologists’ sensitivity and specificity averaged from 1.9% to 11.8% (IQR, 1.9%) and from −4.0% to 3.1% (IQR, 1.6%), respectively, across all pathologies (Extended Data Fig. 3). In comparison, the range of unassisted sensitivities spanned from 20.0% to 92.7% (IQR, 15.3%), whereas the range of unassisted specificities ranged from 81.5% to 99.2% (IQR, 4.0%). These findings indicate substantial heterogeneity in treatment effects on sensitivity and specificity, which aligns with observations regarding absolute error.

### Experience-based characteristics as predictors

We studied whether experience-based radiologist characteristics could function as potential predictors of treatment effect. Specifically, we examined three characteristics: years of experience (explored in previous work^24–28^), subspecialty in thoracic radiology (explored in previous work^24^) and experience with AI tools (an understudied characteristic that approximates the ability to use AI). These characteristics were collected through a post-experiment survey completed by 136 radiologists.

**Fig. 1:**
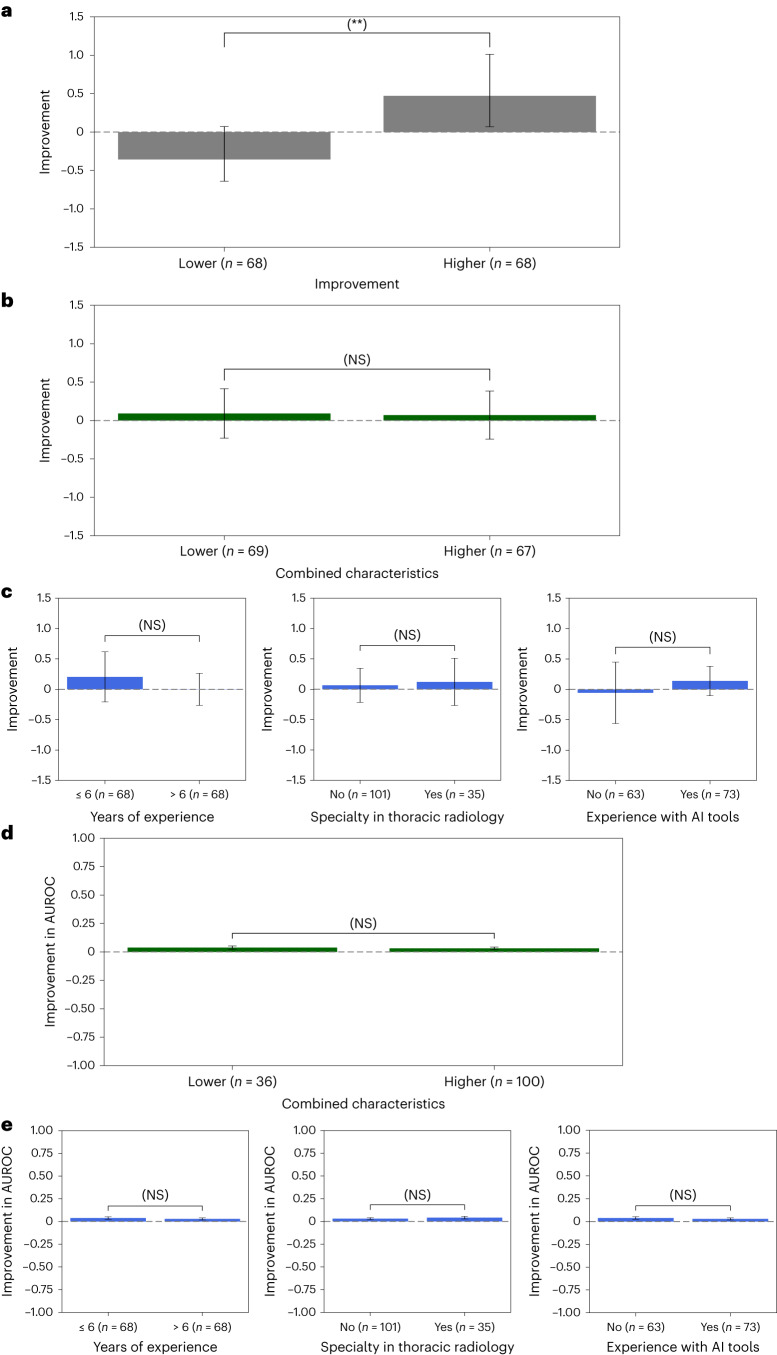
Experience-based radiologist characteristics as predictors of treatment effect on all pathologies aggregated. **a**, Heterogeneous treatment effects of subgroups of radiologists on all pathologies aggregated. The treatment effects were shrunk toward the mean using the empirical Bayes method. Statistically significant heterogeneity was observed between subgroups (*P* = 3.50 × 10^−34^), where radiologists with a higher than median treatment effect had a significantly higher treatment effect of 0.472 (95% CI: 0.403 to 0.541) than those with a treatment effect lower than or equal to the median of −0.357 (95% CI: −0.429 to 0.284). A two-sided, unpaired *t*-test between the two subgroups of treatment effects was conducted. Testing for all pathologies aggregated did not constitute multiple hypothesis testing. The error bars show 95% CIs. **b**, Treatment effects on all pathologies aggregated of subgroups of radiologists based on combined characteristics of years of experience, subspecialty in thoracic radiology and experience with AI tools. No statistically significant difference was observed between the lower predicted treatment effect subgroup, 0.091 (95% CI: −0.231 to 0.413), and the higher predicted treatment effect subgroup, 0.070 (95% CI: −0.243 to 0.383) (*P* > 0.05). The Wald test was used to test regression coefficients that estimate treatment effects against the null hypothesis of joint equality among treatment effects of different subgroups. Details of the statistical models are available in the Methods. There are 136 radiologists with available survey data on the three characteristics. The error bars show 95% CIs. NS indicates no statistical significance (*P* > 0.05). **c**, Same subfigures as in **a** and **b** based on years of experience (left), subspecialty in thoracic radiology (middle) and experience with AI tools (right), respectively. The same statistical test as in **b** was used. **d**, Same subfigure as in **b** for AUROC on all pathologies aggregated. The same statistical test as in **b** was used. **e**, Same subfigures as in **d** based on years of experience (left), subspecialty in thoracic radiology (middle) and experience with AI tools (right), respectively. The same statistical test as in **b** was used.

**Extended Data Fig. 4:**
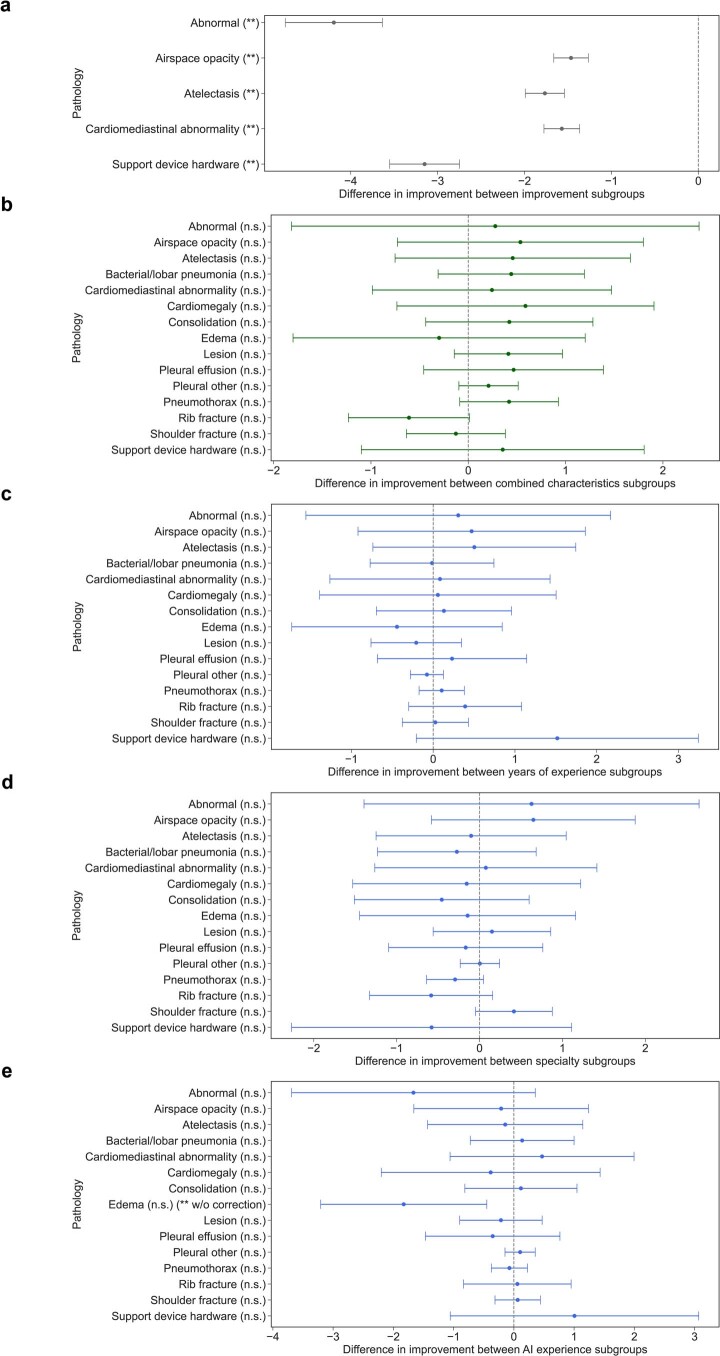
Conventional radiologist characteristics as indicators for treatment effect on individual pathologies. **a**, Difference in treatment effects of subgroups of radiologists on high-prevalence pathology labels (pathology labels with greater than 10% prevalence). The difference is computed between lower and higher improvement subgroups. The error bars show 95% confidence intervals. There are statistically significant differences between subgroups on high-prevalence pathology labels (abnormal B-H adjusted P = 1.66e-29, airspace opacity B-H adjusted P = 7.20e-29, atelectasis B-H adjusted P = 1.10e-30, cardiomediastinal abnormality B-H adjusted P = 4.85e-30, support device hardware B-H adjusted P = 1.57e-30; B-H adjusted P < 0.001). A two-sided, unpaired t-test between the two subgroups of treatment effects was conducted. The difference is -4.194 (95% CI: -4.753 to -3.636) for abnormal, -1.465 (95% CI: -1.664 to -1.266) for airspace opacity, -1.766 (95% CI: -1.991 to -1.541) for atelectasis, -1.571 (95% CI: -1.777 to -1.365) for cardiomediastinal abnormality, and -3.150 (95% CI: -3.552 to -2.748) for support device hardware. 136 radiologists with available survey data are used. **b**, Difference in treatment effects of subgroups of radiologists based on combined characteristics of years of experience, subspecialty in thoracic radiology and experience with AI tools on held-out test sets of radiologists. The difference is computed between lower and higher predicted improvement subgroups. The error bars show 95% confidence intervals. n.s. indicates no statistical significance (B-H adjusted P > 0.05). The Wald test was used to test regression coefficients that estimate treatment effects against the null hypothesis of joint equality among treatment effects of different subgroups. Details of the statistical models are available in Methods. There are 136 radiologists with available survey data on the three characteristics. **c-e**, Difference in treatment effects of subgroups of radiologists based on (**c**) years of experience, (**d**) subspecialty in thoracic radiology, and (**e**) experience with AI tools on 15 individual pathologies. The difference is computed between (**c**) subgroups of fewer versus more years of experience, (**d**) subgroups without versus with subspecialty in thoracic radiology, and (**e**) subgroups without versus with experience using AI tools. The error bars show 95% confidence intervals. n.s. indicates no statistical significance (B-H adjusted P > 0.05). The same statistical test as in **b** was used. There are 136 radiologists with available survey data.

To establish a benchmark, we divided the same 136 radiologists into binary subgroups using an oracle predictor and median treatment effect as the cutoff. We computed the subgroup treatment effects on all pathologies aggregated (Fig. 1a) and high-prevalence pathology labels (Extended Data Fig. 4a) while shrinking the individual radiologist treatment effects using the empirical Bayes method^30^ to ameliorate overestimation of heterogeneity due to measurement error in radiologist performance. We observed a statistically significant difference of −0.828 (232%) between the two subgroups on all pathologies aggregated (*P* < 0.001; Supplementary Table 2) and on each high-prevalence pathology label (Benjamini–Hochberg-adjusted *P* < 0.001), indicating that radiologists with a higher than median treatment effect had a significantly higher treatment effect than those with a treatment effect lower than or equal to the median. This finding suggests a significant heterogeneity between radiologists and shows the extent of heterogeneity that an ideal predictor would have been able to discern.

To understand the predictive power of experience-based radiologist characteristics together, we built a combined characteristics linear regression model that used binary variables for years of experience (whether the radiologist had less than or equal to the median 6 years of experience), subspecialty in thoracic radiology (whether the radiologist specialized in thoracic radiology) and experience with AI tools (whether the radiologist had experience with AI tools) as independent variables and an intercept term to predict the mean treatment effect of each radiologist. We found that the combined characteristics model was a poor predictor of treatment effect on all pathologies aggregated (Fig. 1b) and individual pathologies (Extended Data Fig. 4b). No statistically significant difference was observed in treatment effect between subgroups on all pathologies aggregated (*P* > 0.05; Supplementary Table 3) or on individual pathologies (*P* > 0.05, Benjamini–Hochberg-adjusted *P* > 0.05).

To assess the impact of these characteristics as individual predictors on treatment effects, we divided the radiologists into binary subgroups based on the median value of each predictor. Subsequently, we conducted tests to identify any significant differences in treatment effects between the subgroups created based on the predictor values. When used individually, each of the experience-based characteristics was found to be a poor predictor of treatment effect: no statistically significant difference was observed in treatment effect between subgroups on all pathologies aggregated (*P* > 0.05; Fig. 1c and Supplementary Tables 4–6). Except for the AI experience predictor on edema (*P* = 0.009, Benjamini–Hochberg-adjusted *P* > 0.05), there was also no statistically significant difference between subgroups split based on individual characteristics for any pathology (*P* > 0.05, Benjamini–Hochberg-adjusted *P* > 0.05; Extended Data Fig. 4c).

**Extended Data Fig. 5:**
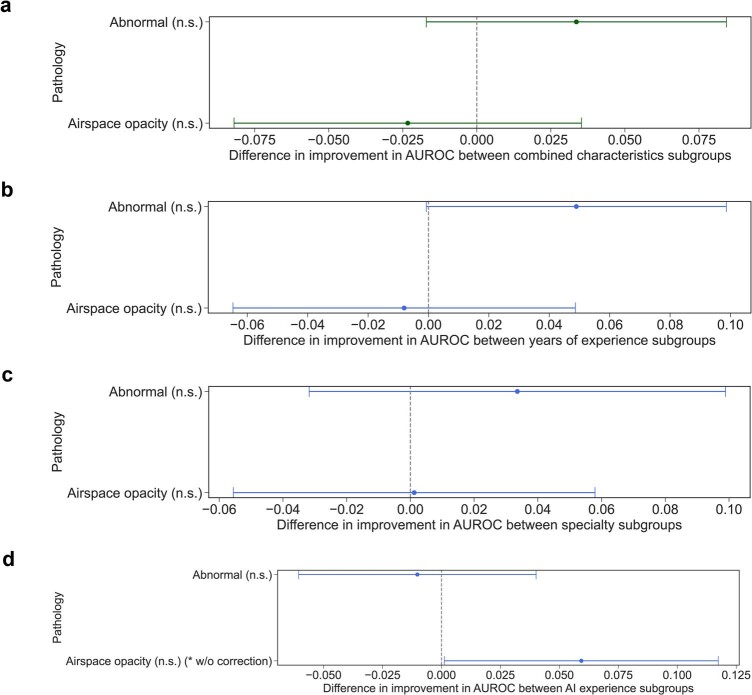
Conventional radiologist characteristics as indicators for treatment effect on AUROC on individual pathologies. **a**, Difference in treatment effects on AUROC of subgroups of radiologists based on combined characteristics of years of experience, subspecialty in thoracic radiology and experience with AI tools on held-out test sets of radiologists. The difference is computed between lower and higher predicted improvement subgroups. The error bars show 95% confidence intervals. n.s. indicates no statistical significance (B-H adjusted P > 0.05). The difference is 0.034 (95% CI: -0.017 to 0.842) for abnormal and -0.023 (95% CI: -0.082 to 0.035) for airspace opacity. The Wald test was used to test regression coefficients that estimate treatment effects against the null hypothesis of joint equality among treatment effects of different subgroups. Details of the statistical models are available in Methods. 136 radiologists with available survey data are used. **b-d**, Difference in treatment effects of subgroups of radiologists based on (**b**) years of experience, (**c**) subspecialty in thoracic radiology, and (**d**) experience with AI tools on 2 individual pathologies on which the AUROC analysis could be computed. The difference is computed between (**b**) subgroups of fewer versus more years of experience, (**c**) subgroups without versus with subspecialty in thoracic radiology, and (**d**) subgroups without versus with experience using AI tools. The error bars show 95% confidence intervals. n.s. indicates no statistical significance (B-H adjusted P > 0.05). The same statistical test as in **a** was used. There are 136 radiologists with available survey data.

In addition to absolute error and calibration performance, we conducted the same analyses for AUROC as the metric and discrimination performance. We found that the combined characteristics model was again a poor predictor of treatment effect on AUROC on all pathologies aggregated (Fig. 1d) and individual pathologies (Extended Data Fig. 5a). No statistically significant difference was observed in treatment effect between subgroups on all pathologies aggregated (*P* > 0.05; Supplementary Table 27) or on individual pathologies (*P* > 0.05, Benjamini–Hochberg-adjusted *P* > 0.05). Each of the individual experience-based characteristics was also found to be a poor predictor of treatment effect on AUROC: no statistically significant difference was observed in treatment effect between subgroups on all pathologies aggregated (*P* > 0.05; Fig. 1e and Supplementary Tables 28–30). Except for the AI experience predictor on airspace opacity (*P* = 0.045, Benjamini–Hochberg-adjusted *P* > 0.05), there was also no statistically significant difference between subgroups split based on individual characteristics for any pathology (*P* > 0.05, Benjamini–Hochberg-adjusted *P* > 0.05; Extended Data Fig. 5b).

### Unassisted performance as a predictor of treatment effect

In addition to experience-based radiologist characteristics, we investigated whether the diagnostic skill of radiologists, as measured by their unassisted error on the specific dataset and task, could serve as a viable predictor of treatment effect. We constructed a linear regression model, where the independent variable was the unassisted error and the dependent variable was the treatment effect, accounting for attenuation bias. We employed a split sampling approach, using distinct sets of patient cases to calculate the unassisted error and treatment effect.

**Fig. 2:**
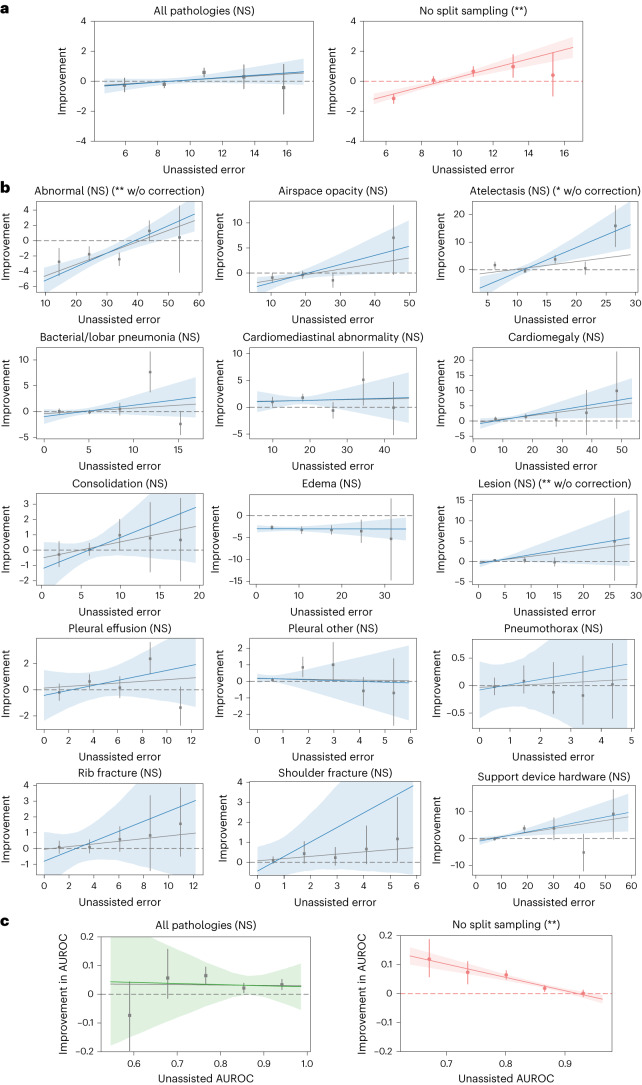
Unassisted error as a predictor of treatment effect. **a**, Unassisted error is a poor predictor of treatment effect on all pathologies aggregated (left). Without split sampling, there is a hallucinated association between treatment effect and unassisted error (right). The binscatter plots contain five evenly spaced bins containing 5,190 data points in total. The gray regression line is fitted on the raw data. The five bins are presented as −0.254 (95% CI: −0.701 to 0.211), −0.205 (95% CI: −0.431 to 0.017), 0.590 (95% CI: 0.291 to 0.878), 0.301 (95% CI: −0.487 to 1.093) and −0.419 (95% CI: −2.178 to 1.125) (left) and −1.148 (95% CI: −1.473 to −0.842), 0.078 (95% CI: −0.165 to 0.313), 0.661 (95% CI: 0.327 to 0.971), 0.979 (95% CI: 0.269 to 1.772) and 0.409 (95% CI: −0.982 to 1.919) (right). The blue dotted regression line is the final regression output after adjusting for attenuation bias. The translucent band around the blue regression line represents the 95% CI. * and ** indicate statistically significant difference from zero at a significance level of 0.05 and 0.01, respectively. NS indicates no statistical significance (*P* > 0.05). **b**, Unassisted error is a poor predictor of treatment effect on each individual pathology. The binscatter plots are designed in the same way as those in **a**. The significance of the slope coefficients is determined through the Benjamini–Hochberg procedure, respectively, to correct for multiple hypothesis testing (15 individual pathologies) at a false discovery rate of 0.05 (*) and 0.01 (**). NS indicates no statistical significance (Benjamini–Hochberg-adjusted *P* > 0.05). **c**, Same subfigures as in **a** for AUROC on all pathologies aggregated. Unassisted AUROC is a poor predictor of treatment effect on AUROC on all pathologies aggregated (left). Without split sampling, there is a hallucinated association between treatment effect on AUROC and unassisted AUROC (right). The five bins are presented as −0.073 (95% CI: −0.196 to 0.044), 0.057 (95% CI: −0.014 to 0.157), 0.065 (95% CI: 0.036 to 0.095), 0.022 (95% CI: 0.006 to 0.038) and 0.033 (95% CI: 0.016 to 0.052) (left) and 0.119 (95% CI: 0.058 to 0.187), 0.074 (95% CI: 0.034 to 0.111), 0.065 (95% CI: 0.050 to 0.079), 0.018 (95% CI: 0.007 to 0.028) and 0.001 (95% CI: −0.011 to 0.012) (right). w/o, without.

We observed that the regression coefficient on unassisted error was positive but not statistically significant on all pathologies aggregated (*P* > 0.05; Fig. 2a, left, and Supplementary Table 7). This finding suggests that unassisted error is an inadequate predictor of treatment effect. Among the individual pathologies, the regression coefficient was significant on abnormal (*P* = 0.005), lesion (*P* = 0.003) and atelectasis (*P* = 0.016) without correcting for multiple hypothesis testing. However, the regression coefficient was not significant on all individual pathologies after correction (Fig. 2b). This suggests that unassisted error also poorly predicts treatment effect at the individual pathology level.

We repeated the same analysis for AUROC and found that the regression coefficient on unassisted AUROC was negative and not statistically significant on all pathologies aggregated (*P* > 0.05; Fig. 2c, left, and Supplementary Table 31). This suggests that unassisted AUROC is also a poor predictor of treatment effect in terms of discrimination performance.

### Preventing reversion to the mean using split sampling

We found that the use of split sampling was crucial in our analysis. This method ensured that unassisted error and treatment effect were calculated using separate sets of patient cases, preventing the spurious correlation caused by reversion to the mean^31^—the statistical phenomenon where subsamples that deviate significantly from the mean are more likely to converge toward the mean in subsequent subsamples. In the context of this study, if a radiologist produces a lower-than-average quality diagnostic assessment on an unassisted case by chance, this same radiologist is very likely to produce a better diagnostic assessment on the same case when assisted, resulting in a positive treatment effect; conversely, if a radiologist produces a good diagnosis on a case unassisted by chance, the radiologist is likely to produce a worse diagnosis on the same case assisted, resulting in a negative treatment effect. This phenomenon, therefore, falsely creates a positive correlation between unassisted error and treatment effect (unassisted error minus assisted error). Split sampling prevents reversion to the mean by using disjoint patient cases to compute unassisted error and treatment effect for each data point in the linear regression model.

To demonstrate the importance of split sampling, we constructed a naive model that involved using all available patient cases to compute the unassisted error (independent variable) and treatment effect (dependent variable). When we applied the naive model, we observed a substantial hallucinated correlation between unassisted error and treatment effect (Fig. 2a, right). On all pathologies aggregated, the hallucinated regression coefficient was 0.357 (adjusted for attenuation bias resulting from measurement error in radiologist performance) and 0.309 (unadjusted), both statistically significant (*P* < 0.001; Supplementary Table 8). Similar hallucinated correlations were observed across individual pathologies (Benjamini–Hochberg-adjusted *P* < 0.05). These findings underscore the necessity of split sampling to mitigate the effects of reversion to the mean.

With AUROC, we observed negative hallucinated correlations between unassisted AUROC and treatment effect on AUROC on all pathologies aggregated (*P* < 0.001; Fig. 2c, right, and Supplementary Table 32) and individual pathologies (Benjamini–Hochberg-adjusted *P* < 0.001). Because AUROC is an aggregate metric over a set of patient cases, the effects of reversion to the mean on AUROC cannot be untangled at the case level. However, the dramatic hallucination of correlations again emphasizes the necessity of split sampling.

### Higher-performing radiologists are still higher performing

Considering the inadequate predictive power of unassisted error on treatment effect, we hypothesized that the relative performance of radiologists with and without AI assistance would remain largely consistent. To test this hypothesis, we constructed a linear regression model that regresses from unassisted error, the independent variable, and an intercept term to assisted error, the dependent variable. We adjusted for attenuation bias on the independent variable. To again avoid reversion to the mean, we adopted a split sampling approach in which we used separate sets of patient cases to compute unassisted error and assisted error for each radiologist.

**Fig. 3:**
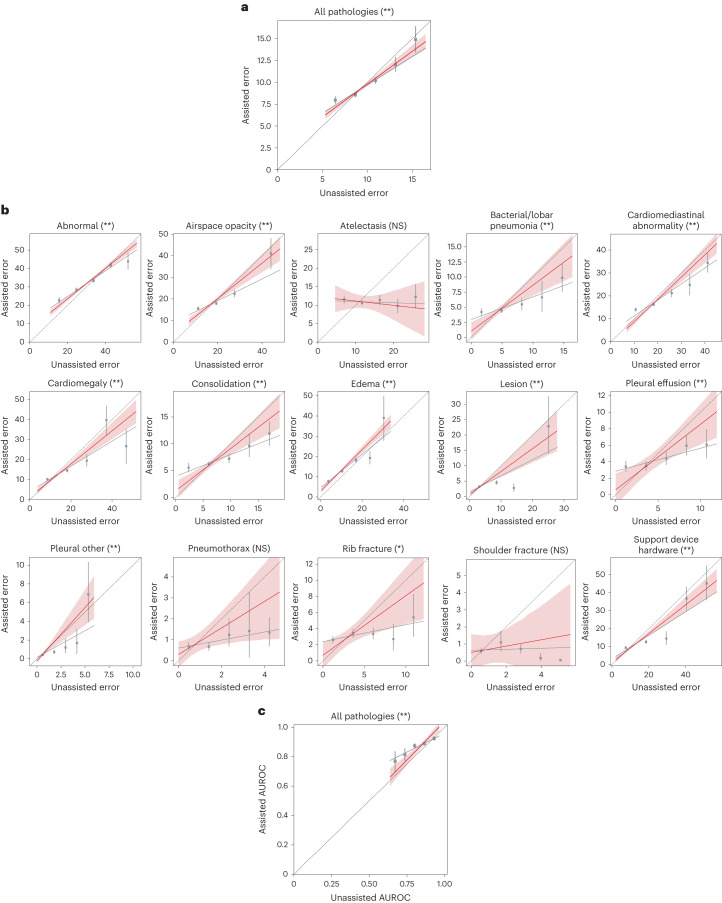
Expected performance of radiologists after receiving AI assistance. **a**, Higher-performing radiologists are still higher performing after receiving AI assistance on all pathologies aggregated. The binscatter plots contain five evenly spaced bins containing 5,190 data points in total. The gray regression line is fitted on the raw data. The five bins are presented as 7.950 (95% CI: 7.579 to 8.339), 8.569 (95% CI: 8.342 to 8.827), 10.197 (95% CI: 9.870 to 10.531), 12.013 (95% CI: 11.245 to 12.857) and 14.870 (95% CI: 13.418 to 16.358). The red dotted regression line is the final regression output after adjusting for attenuation bias. The translucent band around the red regression line represents the 95% CI. * and ** indicate statistically significant difference from zero at a significance level of 0.05 and 0.01, respectively. **b**, Higher-performing radiologists are still higher performing after receiving AI assistance on each individual pathology except for atelectasis, pneumothorax and shoulder fracture, where the regression coefficients for the slope are not statistically significantly different from zero. The binscatter plots are designed in the same way as those in **a**. The five bins for abnormal are presented as 22.712 (95% CI: 21.050 to 24.323), 28.470 (95% CI: 27.411 to 29.528), 33.359 (95% CI: 32.298 to 34.497), 41.729 (95% CI: 40.406 to 43.045) and 43.904 (95% CI: 39.488 to 48.428). The four bins for airspace opacity are presented as 15.590 (95% CI: 14.809 to 16.401), 17.940 (95% CI: 17.198 to 18.718), 22.414 (95% CI: 20.828 to 24.185) and 41.076 (95% CI: 33.894 to 48.061). The five bins for atelectasis are presented as 11.550 (95% CI: 10.558 to 12.505), 10.638 (95% CI: 10.030 to 11.221), 11.366 (95% CI: 10.070 to 12.691), 9.777 (95% CI: 7.909 to 11.676) and 12.262 (95% CI: 9.144 to 15.681). The five bins for bacterial/lobar pneumonia are presented as 4.191 (95% CI: 3.645 to 4.745), 4.443 (95% CI: 4.069 to 4.842), 5.474 (95% CI: 4.499 to 6.499), 6.619 (95% CI: 4.202 to 9.317) and 9.861 (95% CI: 7.620 to 12.169). The five bins for cardiomediastinal abnormality are presented as 14.061 (95% CI: 13.295 to 14.817), 16.268 (95% CI: 15.609 to 16.867), 21.241 (95% CI: 19.556 to 22.881), 24.820 (95% CI: 20.349 to 29.464) and 34.358 (95% CI: 30.263 to 38.553). The five bins for cardiomegaly are presented as 10.169 (95% CI: 9.615 to 10.727), 14.611 (95% CI: 13.737 to 15.516), 19.483 (95% CI: 16.558 to 22.551), 39.736 (95% CI: 32.091 to 46.737) and 26.732 (95% CI: 17.879 to 35.100). The five bins for consolidation are presented as 5.553 (95% CI: 4.727 to 6.387), 6.230 (95% CI: 5.826 to 6.604), 7.219 (95% CI: 6.447 to 8.093), 9.631 (95% CI: 7.575 to 11.707) and 11.915 (95% CI: 9.683 to 14.186). The five bins for edema are presented as 7.761 (95% CI: 7.127 to 8.419), 12.777 (95% CI: 11.909 to 13.616), 18.254 (95% CI: 16.937 to 19.523), 19.268 (95% CI: 16.246 to 22.671) and 39.059 (95% CI: 27.886 to 49.837). The four bins for lesion are presented as 3.185 (95% CI: 2.973 to 3.386), 4.507 (95% CI: 3.856 to 5.170), 2.738 (95% CI: 1.476 to 4.203) and 22.835 (95% CI: 13.974 to 32.555). The five bins for pleural effusion are presented as 3.404 (95% CI: 2.794 to 4.077), 3.473 (95% CI: 2.950 to 4.013), 4.401 (95% CI: 3.644 to 5.173), 5.940 (95% CI: 4.794 to 7.119) and 6.073 (95% CI: 4.431 to 7.915). The five bins for pleural other are presented as 0.410 (95% CI: 0.348 to 0.473), 0.729 (95% CI: 0.529 to 0.938), 1.202 (95% CI: 0.626 to 2.120), 1.668 (95% CI: 0.549 to 3.153) and 6.883 (95% CI: 3.940 to 10.317). The five bins for pneumothorax are presented as 0.689 (95% CI: 0.534 to 0.880), 0.675 (95% CI: 0.493 to 0.891), 1.235 (95% CI: 0.697 to 2.004), 1.425 (95% CI: 0.166 to 3.263) and 1.345 (95% CI: 0.712 to 2.059). The five bins for rib fracture are presented as 2.614 (95% CI: 2.246 to 3.019), 3.405 (95% CI: 2.902 to 3.942), 3.357 (95% CI: 2.699 to 4.112), 2.707 (95% CI: 1.309 to 4.579) and 5.386 (95% CI: 3.032 to 8.266). The five bins for shoulder fracture are presented as 0.594 (95% CI: 0.434 to 0.768), 1.093 (95% CI: 0.582 to 1.765), 0.712 (95% CI: 0.477 to 0.968), 0.171 (95% CI: 0.012 to 0.462) and 0.050 (95% CI: 0.000 to 0.118). The five bins for support device hardware are presented as 9.389 (95% CI: 8.799 to 10.016), 12.652 (95% CI: 11.702 to 13.586), 14.477 (95% CI: 11.126 to 18.418), 36.781 (95% CI: 30.346 to 43.310) and 45.214 (95% CI: 35.945 to 54.983). The significance of the slope coefficients is determined through the Benjamini–Hochberg procedure, respectively, to account for multiple hypothesis testing (15 individual pathologies) at a false discovery rate of 0.05 (*) and 0.01 (**). NS indicates no statistical significance (Benjamini–Hochberg-adjusted *P* > 0.05). **c**, Same subfigure as in **a** for AUROC on all pathologies aggregated. Higher-performing radiologists as measured by AUROC are still higher performing after receiving AI assistance on all pathologies aggregated. The five bins are presented as 0.770 (95% CI: 0.697 to 0.834), 0.814 (95% CI: 0.775 to 0.855), 0.874 (95% CI: 0.859 to 0.888), 0.889 (95% CI: 0.877 to 0.900) and 0.924 (95% CI: 0.911 to 0.936).

The results revealed that the regression coefficient on unassisted error was significantly different from zero when considering all pathologies aggregated (*P* < 0.001; Fig. 3a and Supplementary Table 9). Similarly, the regression coefficient was significant on most individual pathologies (Benjamini–Hochberg-adjusted *P* < 0.05), except for atelectasis, pneumothorax and shoulder fracture (Benjamini–Hochberg-adjusted *P* > 0.05; Fig. 3b). We similarly constructed a linear regression model regressing from unassisted AUROC and an intercept term to assisted AUROC. The regression coefficient on unassisted AUROC was again significant on all pathologies aggregated (*P* < 0.001; Fig. 3c and Supplementary Table 33), whereas the coefficient was insignificant on abnormal (*P* > 0.05). Together, these findings indicate that unassisted error serves as a strong predictor of assisted error in most cases.

### AI error as a predictor of treatment effect

**Fig. 4:**
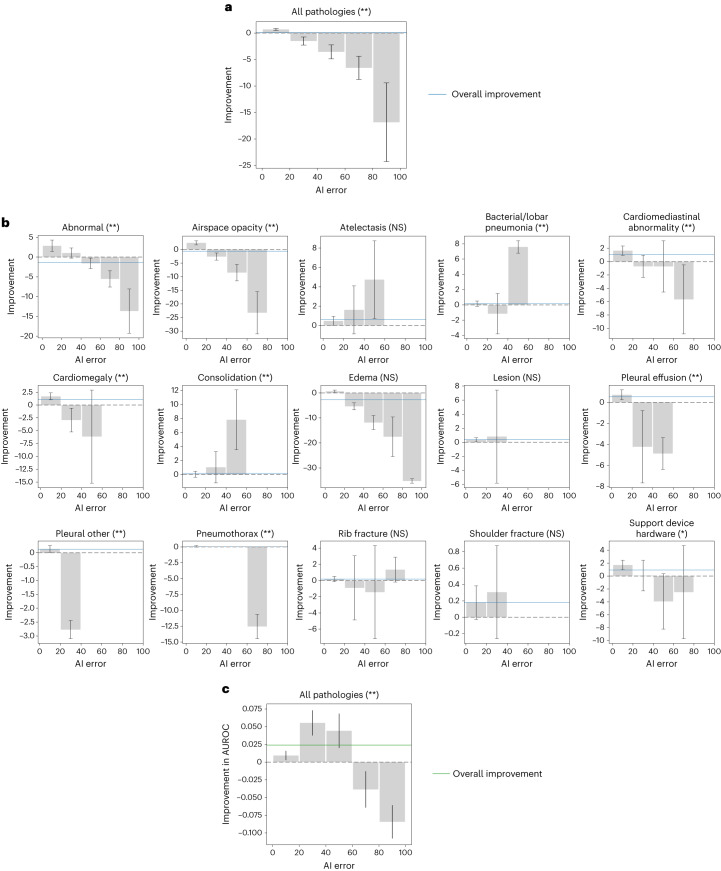
AI error as a predictor of treatment effect. **a**, AI has greater treatment effects on radiologists when the AI assistance has lower error on all pathologies aggregated. The five bins are presented as 0.679 (95% CI: 0.492 to 0.865), −1.509 (95% CI: −2.267 to −0.750), −3.556 (95% CI: −4.878 to −2.235), −6.569 (95% CI: −8.764 to −4.374) and −16.845 (95% CI: −24.288 to −9.403). The error bars show 95% CIs. The blue lines show the overall treatment effect across AI error. * and ** indicate statistically significant difference among subgroups of different AI error through a joint equality test at a significance level of 0.05 and 0.01, respectively. **b**, AI has greater treatment effects on radiologists when the AI assistance has lower error on abnormal, airspace opacity, cardiomediastinal abnormality, cardiomegaly, pleural effusion, pleural other, pneumothorax and support device hardware. The reverse trend holds for bacterial/lobar pneumonia and consolidation. The bar plots are designed in the same way as those in **a**. The significance of the subgroup joint test is determined through the Benjamini–Hochberg procedure, respectively, to correct for multiple hypothesis testing (15 individual pathologies) at a false discovery rate of 0.05 (*) and 0.01 (**). NS indicates no statistical significance (Benjamini–Hochberg-adjusted *P* > 0.05). **c**, AI has greater treatment effects on AUROC on radiologists when the AI assistance has an absolute error in the range [20, 100], whereas the trend is unclear with absolute error in the range [0, 20].

We investigated whether higher-quality AI assistance led to better treatment effects on average across radiologists and cases. We computed the treatment effects of AI when the absolute error of AI-predicted probabilities fell into five separate ranges, and we tested for heterogeneity among AI error ranges by testing the joint null hypothesis of equal treatment effects across bins. We found that different AI error ranges resulted in statistically significant differences in treatment effect on all pathologies aggregated (*P* < 0.001; Fig. 4a and Supplementary Table 10). More accurate AI predictions led to higher treatment effects: AI assistance with absolute error under 20 resulted in a treatment effect of 0.679 (95% confidence interval (CI): 0.492 to 0.865, *n* = 176,130; Supplementary Table 11), whereas AI assistance with absolute error above 80 resulted in a treatment effect of −16.845 (95% CI: −24.288 to −9.403, *n* = 371). No singular trend was observed across individual pathologies. On abnormal, airspace opacity, bacterial/lobar pneumonia, cardiomediastinal abnormality, cardiomegaly, consolidation, pleural effusion, pleural other, pneumothorax and support device hardware, different AI ranges resulted in statistically significant differences in treatment effect (Benjamini–Hochberg-adjusted *P* < 0.05 for support device hardware, Benjamini–Hochberg-adjusted *P* < 0.01 for all others). More accurate AI predictions led to better treatment effects on abnormal, airspace opacity, cardiomediastinal abnormality, cardiomegaly, pleural effusion, pleural other, pneumothorax and support device hardware; the reverse trend held for bacterial/lobar pneumonia and consolidation, whereas the trend was unclear for the remaining pathologies (Fig. 4b).

We conducted the same analysis using AUROC, where we similarly separated predictions into five bins based on the absolute error of the AI-predicted probabilities based on binary ground truth labels and computed the treatment effect on AUROC over all predictions in each bin. We found that different AI error ranges again resulted in statistically significant differences in treatment effect on AUROC on all pathologies aggregated (*P* < 0.001; Fig. 4c and Supplementary Table 34). The overall trend was unclear: more accurate AI predictions led to higher treatment effects on AUROC when the AI assistance had an absolute error in the range [20, 100], but treatment effects in the range [0, 20] were smaller than those in the range [20, 60]. For individual pathologies, the AUROC analysis was numerically feasible only for airspace opacity, and the trend was unclear.

These findings suggest that AI error could be a predictor of treatment effect, but the statistical significance and direction of the relationship could differ across pathologies and metrics.

### AI that underestimates probabilities leads to better effect

We subsequently examined the impact of the direction of AI error on the resulting treatment effect. We computed the treatment effects of AI across 10 different ranges of signed error, which represents the difference between AI-predicted probabilities and the corresponding ground truth probabilities. Heterogeneity among these AI error ranges was tested using a joint equality hypothesis.

**Fig. 5:**
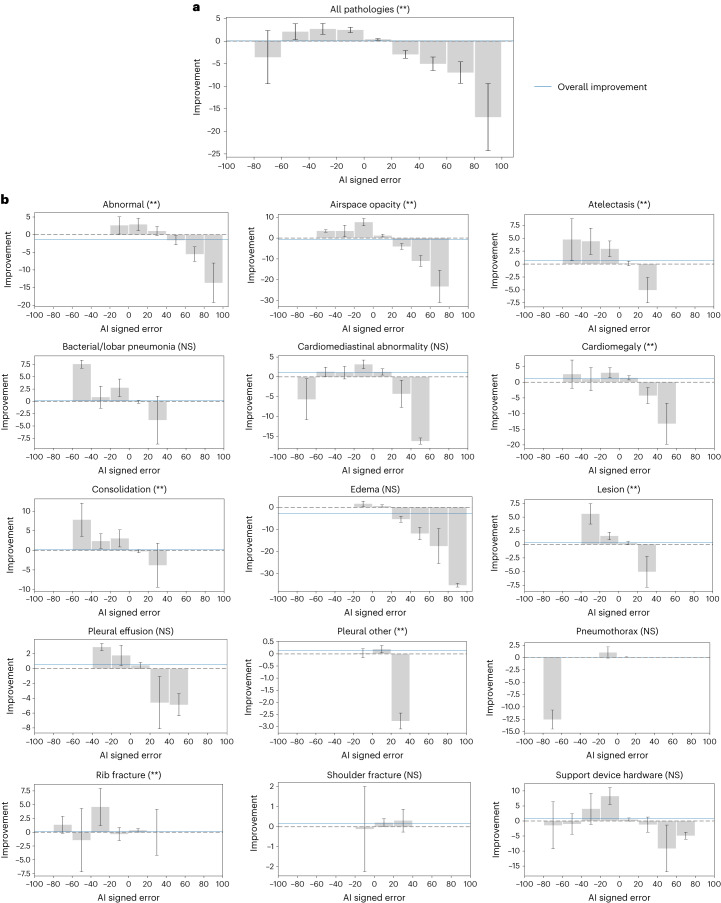
AI signed error as a predictor of treatment effect. **a**, AI has greater treatment effects on radiologists when the AI assistance underestimates probabilities, rather than overestimates probabilities, given the same absolute error on all pathologies aggregated. The nine bins are presented as −3.560 (95% CI: −9.472 to 2.353), 2.089 (95% CI: 0.334 to 3.844), 2.698 (95% CI: 1.497 to 3.899), 2.483 (95% CI: 1.897 to 3.070), 0.398 (95% CI: 0.235 to 0.561), −2.933 (95% CI: −3.787 to −2.079), −5.018 (95% CI: −6.516 to −3.519), −6.968 (95% CI: −9.346 to −4.591) and −16.845 (95% CI: −24.288 to −9.403). The error bars show 95% CIs. The blue bars show the overall treatment effect across AI error. * and ** indicate statistically significant difference among subgroups of different AI signed error through a joint equality test at a significance level of 0.05 and 0.01, respectively. **b**, AI has greater treatment effects on radiologists when the AI assistance underestimates probabilities, rather than overestimates probabilities, given the same absolute error on airspace opacity, atelectasis, cardiomegaly, consolidation and lesion. The bar plots are designed in the same way as those in **a**. The significance of the subgroup joint test is determined through the Benjamini–Hochberg procedure, respectively, to correct for multiple hypothesis testing (15 individual pathologies) at a false discovery rate of 0.05 (*) and 0.01 (**). NS indicates no statistical significance (Benjamini–Hochberg-adjusted *P* > 0.05).

We found that different ranges of AI signed error resulted in statistically significant differences in treatment effect on all pathologies aggregated (*P* < 0.001; Supplementary Tables 12 and 13). We observed that AI predictions with negative errors, indicating underestimation of probabilities by the AI, led to better treatment effects compared to predictions with the same magnitude of positive errors, indicating overestimation of probabilities by the AI (Fig. 5a).

No singular trend was observed across individual pathologies. For eight pathologies (abnormal, airspace opacity, atelectasis, cardiomegaly, consolidation, lesion, pleural other and rib fracture), different AI error ranges showed statistically significant differences in treatment effect (Benjamini–Hochberg-adjusted *P* < 0.01). Among these pathologies, AI predictions that underestimated probabilities led to better treatment effects on airspace opacity, atelectasis, cardiomegaly, consolidation and lesion, whereas the trend was unclear for the remaining pathologies (Fig. 5b).

The same analysis could not be repeated for AUROC, because there were not both cases with the pathology present and cases with the pathology not present in each non-empty bin on all pathologies aggregated or any individual pathology, causing the AUROC to be undefined for most bins.

### Alternative measures of performance

We found consistent results as the ones introduced in earlier sections using alternative measures of performance. Specifically, in addition to using (1) absolute error and signed error with continuous ground truth probabilities and (2) AUROC as the metric for radiologist or AI performance, we conducted the proposed analyses using absolute error and signed error with binary ground truth labels, which were computed by thresholding the continuous ground truth probabilities at 50. Results are shown in Supplementary Tables 14–26.

Under binary ground truth labels, the relationships between experienced-based characteristics and treatment effect found earlier under continuous ground truth probabilities held for all pathologies aggregated and individual pathologies (Supplementary Tables 16–19). The relationships between unassisted error and treatment effect held except for lesion, where it was not statistically significant under continuous ground truth probabilities but was significant under binary ones (Supplementary Table 20). The relationships between unassisted error and treatment effect without split sampling held except for edema, where it was statistically significant at a significance level of 0.05 under continuous ground truth probabilities but was significant at 0.01 under binary ground truth probabilities (Supplementary Table 21). The relationships between unassisted error and assisted error held except for rib fracture, where it was statistically significant at a significance level of 0.05 under continuous ground truth probabilities but was significant at 0.01 under binary ground truth probabilities (Supplementary Table 22). The relationships between AI error and treatment effect held for all pathologies aggregated (Supplementary Tables 23 and 25). The relationships on individual pathologies also varied as they did with continuous ground truth probabilities and did not show a singular trend.

The overall consistencies of the results presented show the general applicability of the findings to different ways of measuring radiologist and AI performance.

## Discussion

In this study, we investigated the heterogeneous treatment effects of AI assistance on radiologists for chest X-ray diagnosis. Our findings, based on a large-scale sample of 140 radiologists, highlight the existence of radiologist heterogeneity in treatment effects, which has substantial implications for both absolute and relative performance. These results underscore the inadequacy of a one-size-fits-all approach to AI assistance and emphasize the importance of individualized strategies to maximize benefits and minimize potential harms. Understanding who benefits from AI assistance and who is negatively impacted is crucial for effectively targeting AI assistance.

We found that experience-based radiologist characteristics, including years of experience, subspecialty in thoracic radiology and experience with AI tools, did not serve as reliable predictors of treatment effect, in terms of both calibration performance and discrimination performance. These findings challenge the associations between experience-based radiologist characteristics and the treatment effect of AI assistance reported in previous research^24–28^. The observed variability could be attributed to our larger and more diverse sample size, encompassing 140 radiologists with varying skill levels, experiences and preferences. Additionally, our study’s inclusion of a wide range of diagnostic tasks enables a robust examination of the complex factors influencing the treatment effect. Furthermore, the performance characteristics and quality of the specific AI system may play an important role, highlighting the need for developers to consider these factors when deploying AI assistance. To optimize the implementation of AI assistance, a comprehensive assessment of multiple factors, including the clinical task, patient population and AI system, is essential.

Similarly, direct measures of diagnostic skill, such as unassisted error, showed limited predictive power for treatment effects. This finding again holds for both calibration performance and discrimination performance. Surprisingly, radiologists who initially performed poorly without AI assistance did not necessarily benefit more or experience more harm from AI assistance compared to higher-performing counterparts. We demonstrate that proper use of statistical methods, such as split sampling, is crucial to avoid spurious associations between unassisted error and treatment effect and ensures reliable conclusions about the predictive power of unassisted error. Future research should consider cognitive abilities, adaptability to new technologies and decision-making processes as potential predictors. Developing accurate predictive models to identify radiologists who are more likely to benefit from AI assistance holds promise for future investigations. Without reliable predictors, it is necessary to measure radiologists’ response to AI assistance under realistic simulations of deployment settings before deciding whether to provide AI assistance to different radiologists. For example, it may be necessary to directly measure a radiologist’s treatment effect from the assistive AI system on an experimental dataset that is representative of the target patient population.

In addition to investigating the radiologist characteristics that can impact AI’s treatment effect, we showed that higher-quality AI assistance leads to better treatment effects in terms of calibration performance measured by absolute error, whereas the trend was unclear in terms of discrimination performance measured by AUROC. Our results indicate that AI predictions with smaller errors lead to better treatment effects on all pathologies aggregated, highlighting the importance of developing more accurate AI models for assistance. Conversely, AI predictions with large errors tend to lead to negative treatment effects, suggesting that radiologists struggle to consistently distinguish between accurate and inaccurate AI predictions and can be misled by inaccurate AI predictions. Moreover, we observed that, given the same absolute error, AI predictions that underestimate the ground truth probabilities can lead to better treatment effects than predictions that overestimate them on all pathologies aggregated. Apart from improving AI accuracy, it is valuable to help radiologists better identify inaccurate AI predictions. For example, assistive AI systems that provide explanations for their predictions^32^ or generate nuanced radiology reports^33–37^, rather than probabilities alone, may allow radiologists to potentially extract value from inaccurate AI predictions. In addition, we emphasize that these findings between AI accuracy and treatment effect are the result of many factors simultaneously at play, including the ground truth probability, the radiologist’s predicted probability and how radiologists interpret and use AI assistance, which can all be correlated with AI’s predicted probability. Therefore, these findings should not be extrapolated for defining the cognitive mechanism in which AI assistance helps or hurts radiologists. Further research with explicit control of the potential factors is necessary to understand that underlying mechanism^29^.

Our study has several limitations that should be acknowledged. First, the randomization of treatment conditions in the experiment, although necessary to eliminate confounding factors, prevented the analysis of temporal trends in radiologists’ response to AI assistance. We were unable to assess whether radiologists improved in incorporating AI predictions over time as they encountered more patient cases. Future research should aim to investigate these evolving dynamics between radiologists and AI. Second, the AI assistance available to radiologists contained only predicted probabilities and did not include additional explanations, such as localization of pathologies, which could help radiologists more accurately interpret and, therefore, make better use of the available AI predictions. Designers of AI systems should investigate the optimal types of explanations to present and the mode of presentation while staying cautious of the increased cognitive burden that this additional information can bring. Another limitation is the lack of exploration into the impact of task granularity. The AI model generated predictions for 15 individual pathologies, some of which were interconnected and represented different levels of detail. For instance, airspace opacity encompasses pathologies such as atelectasis, edema and consolidation. Understanding the relationships between higher-level and lower-level pathologies would be valuable in future studies. Furthermore, due to the simultaneous presentation of all 15 AI predictions, it was challenging to isolate the effect of AI assistance on individual pathologies. The influence of AI predictions on one pathology could potentially affect the radiologists’ response to AI predictions on other pathologies, especially when they are interrelated. Additionally, because we provided actual AI predictions on patient cases to radiologists, it was also difficult to eliminate the confounding factor of the patient case when studying the relationship between the accuracy of AI predictions and the radiologist’s treatment effect. Future work may control for the influence of the patient case by providing artificially set predictions to radiologists.

In conclusion, our study underscores the need for individualized approaches that are aware of clinician heterogeneity, high-quality AI models and comprehensive assessments of multiple factors to optimize the implementation of AI assistance in clinical medicine. Collaboration between clinicians and AI developers, focusing on personalized strategies and continuous improvement of AI models, will be essential for achieving the full potential of clinician–AI collaboration in healthcare.

## Methods

This research complied with all relevant ethical regulations. The study that produced the AI assistance dataset^29^ used in this study was determined by the Massachusetts Institute of Technology (MIT) Committee on the Use of Humans as Experimental Subjects to be exempt through exempt determination E-2953.

### Dataset specification

This study used 324 retrospective patient cases from Stanford University’s healthcare system containing chest X-rays and clinical histories, which include patients’ indication, vitals and labs. In this study, we analyzed data collected from a total of 140 radiologists participating in two experiment designs. The non-repeated-measure design included 107 radiologists in a non-repeated-measure setup (Supplementary Fig. 1). Each radiologist read 60 patient cases across four subsequences that each contained 15 cases. Each subsequence corresponded to one of four treatment conditions: with AI assistance and clinical histories, with AI assistance and without clinical history, without AI assistance and with clinical histories and without AI assistance and clinical histories. The four subsequences and associated treatment conditions were organized in a random order. The 60 patient cases were randomly selected and randomly assigned to one of the treatment conditions. This design included across-subject and within-subject variations in the treatment conditions; it did not allow within-case-subject comparisons because a case was encountered only once for a radiologist^38^. Order effects were mitigated by the randomization of treatment conditions. The repeated-measure design included 33 radiologists in a repeated-measure setup (Supplementary Fig. 2). Each radiologist read a total of 60 patient cases, each under each of the four treatment conditions and producing a total of 240 diagnoses. The radiologist completed the experiment in four sessions, and the radiologist read the same 60 randomly selected patient cases in each session under each of the various treatment arms. In each session, 15 cases were read in each treatment arm in batches of five cases. Treatments were randomly ordered. This resulted in the radiologist reading each patient case under a different treatment condition over the four sessions. There was a 2-week washout period^15,39,40^ between every session to minimize order effects of radiologists reading the same case multiple times. This design included across-subject and within-subject variations as well as across-case-radiologist and within-case-radiologist variations in treatment conditions. Order effects were mitigated by the randomization of treatment conditions. No enrichment was applied to the data collection process. We combined data from both experiment designs from the clinical history conditions. Further details about the data collection process are available in a separate study^29^, which focuses on establishing a Bayesian framework for defining optimal human–AI collaboration and characterizing actual radiologist behavior in incorporating AI assistance. The study was determined exempt by the MIT Committee on the Use of Humans as Experimental Subjects through exempt determination E-2953.

There are 15 pathologies with corresponding AI predictions: abnormal, airspace opacity, atelectasis, bacterial/lobar pneumonia, cardiomediastinal abnormality, cardiomegaly, consolidation, edema, lesion, pleural effusion, pleural other, pneumothorax, rib fracture, shoulder fracture and support device hardware. These pathologies, the interrelations among these pathologies and additional pathologies without AI predictions can be visualized in a hierarchical structure in Supplementary Fig. B.1. Radiologists were asked to familiarize themselves with the hierarchy before starting, had access to the figure throughout the experiment and had to provide predictions for pathologies following this hierarchy. This aimed to maximize clarity on the specific pathologies referenced in the experiment. When radiologists received AI assistance, they were simultaneously presented with the AI predictions for these 15 pathologies along with the patient’s chest X-ray and, if applicable, their clinical history. The AI predictions were presented in the form of prediction probabilities on a 0–100 scale. The AI predictions were generated by the CheXpert model^8^, which is a DenseNet121 (ref. ^41^)-based model for chest X-rays that has been shown to perform similarly to board-certified radiologists. The model generated a single prediction for fracture that was used as the AI prediction for both rib fracture and shoulder fracture. Authors of the CheXpert model^8^ decided on the 14 pathologies (with a single prediction for fracture) based on the prevalence of observations in radiology reports in the CheXpert dataset and clinical relevance, conforming to the Fleischner Society’s recommended glossary^42^ whenever applicable. Among the pathologies, they included ‘Pneumonia’ (corresponding to ‘bacterial/lobar pneumonia’) to indicate the diagnosis of primary infection and ‘No Finding’ (corresponding to ‘abnormal’) to indicate the absence of all pathologies. These pathologies were set in the creation of the CheXpert labeler^8^, which has been applied to generate labels for reports in the CheXpert dataset and MIMIC-CXR^43^, which are among the largest chest X-ray datasets publicly available.

The ground truth probabilities for a patient case were determined by averaging the continuous predicted probabilities of five board-certified radiologists from Mount Sinai Hospital with at least 10 years of experience and chest radiology as a subspecialty on a 0–100 scale. For instance, if the predicted probabilities of the five board-certified radiologists are 91, 92, 92, 100 and 100, respectively, the ground truth probability is 95. The prevalence of the pathologies based on a ground truth probability threshold of 50 of a pathology being present is shown in Supplementary Table 1.

The participating radiologists represent a diverse set of institutions recruited through two means. Their primary affiliations include large, medium and small clinical settings and non-clinical settings. Additionally, some radiologists are affiliated with an academic hospital, whereas others are not. Radiologists in the non-repeated-measure design were recruited from teleradiology companies. Radiologists in the repeated-measure design were recruited from the Vinmec health system in Vietnam. Details about the participating radiologists and recruitment process can be found in Supplementary Note | Participant recruitment and affiliation.

The experiment interface and instructions presented to participating radiologists can be found in Supplementary Note | Experiment interface and instructions. Before entering the experiment, radiologists were instructed to walk through the experiment instructions, the hierarchy of pathological findings, basic information and performance of the AI model, video demonstration of the experiment interface and examples, consent clauses, comprehension check questions, information on bonus payment that incentivizes effort and practice patient cases covering four treatment conditions and showing example AI predictions from the AI model used in the experiment.

Sex and gender statistics of the participating radiologists and patient cases are available in Supplementary Tables 39 and 40, respectively. Sex and gender were not considered in the original data collection procedures. Disaggregated information about sex and gender at the individual level was collected in the separate study and will be made available^29^.

### Empirical Bayes for individual heterogeneity

We used the empirical Bayes method^30^ to shrink the raw mean heterogeneous treatment effects and performance metrics of individual radiologists measured on the dataset toward the grand mean to ameliorate overestimating heterogeneity due to sampling error. The values include AI’s treatment effects on error, sensitivity and specificity and performance metrics on unassisted error, sensitivity and specificity.

Assume that ${t}_{r}$ is radiologist *r*’s true mean treatment effect from AI assistance or any metric of interest. We observe

1 $$\tilde{t}_{r}={t}_{r}+{{{\eta }}}_{r}$$

which differs from ${t}_{r}$ by ${{{\eta }}}_{r}$. We use a normal distribution as the prior distribution over the metric of interest. The mean of the prior distribution can be computed as

2 $$E\left[\tilde{t}_{r}\right]=E\left[{t}_{r}\right],$$

the mean of the observed mean metric of interest of radiologists. The variance of the prior distribution can be computed as

3 $$E\left[{\Big({t}_{r}-E\left[{t}_{r}\right]\Big)}^{2}\right]=E\left[{\left(\tilde{t}_{r}-E\left[\tilde{t}_{r}\right]\right)}^{2}\right]-E\left[{{{\eta }}}_{r}^{2}\right],$$

the variance of the observed mean metric of interest of radiologists minus the estimated $E\left[{{{\eta }}}_{r}^{2}\right]$. We can estimate $E\left[{{{\eta }}}_{r}^{2}\right]$ with

4 $$E\left[{{{\eta }}}_{r}^{2}\right]=E\left[{\left(\frac{1}{{N}_{r}}\mathop{\sum }\limits_{i}{t}_{{ir}}-E\left[{t}_{{ir}}\right]\right)}^{2}\right]=E\left[\frac{{\sum }_{i}{\left({t}_{{ir}}-E\left[{t}_{{ir}}\right]\right)}^{2}}{{N}_{r}}\right]=E\left[s.e.{\left(\tilde{t}_{r}\right)}^{2}\right].$$

Denote the estimated mean and variance of the prior distribution as ${{\rm{\mu }}}_{0}$ and ${{\rm{\sigma }}}_{0}^{2}$. We can compute the mean of the posterior distribution for radiologist $r$ as

5 $$\frac{{{\rm{\sigma }}}_{r}^{2}{{\rm{\mu }}}_{0}+{{\rm{\sigma }}}_{0}^{2}{{\rm{\mu }}}_{r}}{{{\rm{\sigma }}}_{0}^{2}+{{\rm{\sigma }}}_{r}^{2}}$$

where ${{\rm{\mu }}}_{r}=\widetilde{{t}}_{t}$ and ${{\rm{\sigma }}}_{r}=s.e.\left(\widetilde{{t}}_{r}\right)$; we can compute the variance of the posterior as

6 $$\frac{{{\rm{\sigma }}}_{0}^{2}{{\rm{\sigma }}}_{r}^{2}}{{{\rm{\sigma }}}_{0}^{2}+{{\rm{\sigma }}}_{r}^{2}}$$

where ${{\rm{\sigma }}}_{r}=s.e.\left(\widetilde{{t}}_{r}\right)$. The updated mean of the posterior distribution is the radiologist’s metric of interest after shrinkage.

For the analysis on treatment effects on absolute error, we focus on high-prevalence pathologies with prevalence greater than 10%, because radiologists’ baseline performance without AI assistance is generally highly accurate on low-prevalence pathologies, where they correctly predict that a pathology is not present, and, as a result, there is little variation in radiologists’ errors. This is especially true when computing each individual radiologist’s treatment effect. When there is zero variance in the performance of a radiologist under a treatment condition, the associated standard error estimate is zero, making it impossible to perform inference on this radiologist’s treatment effect.

### Combined characteristics model for splitting radiologists into subgroups

The combined characteristics model was fitted on a training set of half of the radiologists (*n* = 68) to predict treatment effects of the test set of the remaining half (*n* = 68). The treatment effect predictions on the test set were used as the combined characteristics score for splitting the test set radiologists into binary subgroups (based on whether a particular radiologist’s combined characteristics score was smaller than or equal to the median treatment effect of radiologists computed from all available reads). Then, the same procedure was repeated after flipping the training set and test set radiologists to split the other set of radiologists into binary subgroups. The experience-based characteristics of radiologists in the randomly split training set and test set were balanced: one set contained 27 radiologists with less than or equal to 6 years of experience and 41 radiologists with more than 6 years of experience, and the other set contained 41 and 27, respectively. One set contained 47 radiologists who did not specialize in thoracic radiology and 21 radiologists who did, and the other set contained 54 and 14 radiologists, respectively. One set contained 32 radiologists without experience with AI tools and 36 radiologists with experience, and the other set contained 31 and 37, respectively.

### Treatment effect models

To compute a radiologist’s observed mean treatment effect and the corresponding standard errors and the overall treatment effect of AI assistance across subgroups, we built a linear regression model with the following formulation using the statsmodels library: *error* ∼ *1* + *C(treatment)*. Here, error refers to the absolute error of a radiologist prediction; 1 refers to an intercept term; and treatment refers to a binary indicator of whether the prediction is made with or without AI assistance. This formulation allows us to compute the treatment effect of AI assistance for both non-repeated-measure and repeated-measure data.

### Subgroup-specific treatment effect models

For the analyses on experience-based radiologist characteristics and AI error, we computed the treatment effects of subgroups split based on the predictor of interest by building a linear regression model with the following formulation using the statsmodels library: *error* ∼ *1* + *C(subgroup)* + *C(treatment):C(subgroup)*. Here, error refers to the absolute error of a radiologist prediction; 1 refers to an intercept term; subgroup refers to an indicator of the subgroup that the radiologist is split into; and treatment refers to a binary indicator of whether the prediction is made with or without AI assistance. This formulation allows us to compute the subgroup-specific treatment effect of AI assistance for both non-repeated-measure data and repeated-measure data.

### Cluster-robust standard errors

To account for correlations of observations within patient cases and radiologists, we computed cluster-robust standard errors that are two-way clustered at the patient case and radiologist level for all inferences unless otherwise specified^44,45^. With the statsmodels library’s ordinary least squares (OLS) class, we used a clustered covariance estimator as the type of robust sandwich estimator and defined two-way groups based on identifiers of the patient cases and radiologists. The approach assumes that regression model errors are independent across clusters defined by the patient cases and radiologists and adjusts for correlations within clusters.

### Reversion to the mean

The reversion to the mean effect and the mechanism of split sampling in avoiding reversion to the mean are explained in the following derivation:

Suppose that ${u}_{i,r}^{* }$ and ${a}_{i,r}^{* }$ are the true unassisted and assisted diagnostic error of radiologist $r$ on patient case *i*. Suppose that we measure ${u}_{i,r}={u}_{i,r}^{* }+{e}_{i,r}^{u}$ and ${a}_{i,r}={a}_{i,r}^{* }+{e}_{i,r}^{a}$ where ${e}_{i,r}^{u}$ and ${e}_{i,r}^{a}$ are measurement errors. Assume that the measurement errors are independent of ${u}_{i,r}^{* }$ and ${a}_{i,r}^{* }$.

To study the relationship between unassisted error and treatment effect, we intend to build the following linear regression model:

7 $${u}_{r}^{* }-{a}_{r}^{* }={{\beta }}{u}_{r}^{* }+{e}_{r}^{* }$$

where the error is independent of the independent variable, and ${u}_{r}^{* }$ and ${a}_{r}^{* }$ are the mean unassisted and assisted performance of radiologist $r$. Here, the moment condition

8 $$E\left[{e}_{i,r}^{* }\times {u}_{i,r}^{* }\right]=0$$

is as desired. This univariate regression estimates the true value of ${{\beta }}$, which is defined as

9 $$\frac{{\rm{Cov}}({{\rm{u}}}_{{\rm{r}}}^{\ast }-{{\rm{a}}}_{{\rm{r}}}^{\ast },\,{{\rm{u}}}_{{\rm{r}}}^{\ast })}{{\rm{Var}}({{\rm{u}}}_{{\rm{r}}}^{\ast })}$$

However, because we have access only to noisy measurements ${u}_{r}$ and ${a}_{r}$, consider instead an approach that builds the model

10 $${u}_{r}-{a}_{r}={{\beta }}{u}_{r}+{e}_{r}$$

and assumes the moment condition

11 $$E\left[{e}_{r}\times {u}_{r}\right]=0.$$

This linear regression model using noisy measurements instead generates the following estimate of ${{\beta }}$:

12 $$\frac{{Cov}\left({u}_{r}-{a}_{r},{u}_{r}\right)}{{Var}\left({u}_{r}\right)}=\frac{{Cov}\left({u}_{r}^{* }-{a}_{r}^{* },{u}_{r}^{* }\right)+{Var}\left({e}_{r}^{u}\right)}{{Var}\left({u}_{r}^{* }\right)+{Var}\left({e}_{r}^{u}\right)}$$

which is incorrect because of the additional ${{V}}\,{{ar}}\left({{{e}}}_{{{r}}}^{{{u}}}\right)$ terms in the numerator and the denominator. The additional term in the denominator represents attenuation bias, which we address in detail in a later subsection. The term in the numerator represents the reversion to the mean issue, which we now discuss in further detail.

As the equation shows, the bias caused by reversion to the mean is positive. This term exists because the moment condition $E\left[{e}_{r}\times {u}_{r}\right]=0$, equation (11), is not valid at the true value of ${{\beta }}$ as shown in the following derivation:

$$\begin{array}{c}E\left[\left({u}_{r}-{a}_{r}-{{\beta }}{u}_{r}\right)\times {u}_{r}\right]=E\left[\left(\left(1-{{\beta }}\right){u}_{r}-{a}_{r}\right)\times {u}_{r}\right]\\ \begin{array}{c}=E\left[\left(\left(1-{{\beta }}\right)\left({u}_{r}^{* }+{e}_{r}^{u}\right)-\left({a}_{r}^{* }+{e}_{r}^{a}\right)\right)\times {u}_{r}\right]\\ \begin{array}{c}=E\left[\left(\left(\left(1-{{\beta }}\right){u}_{r}^{* }-{a}_{r}^{* }\right)+\left(1-{{\beta }}\right){e}_{r}^{u}-{e}_{r}^{a}\right)\times {u}_{r}\right]\\ \begin{array}{c}=E\left[\left({e}_{r}^{* }+\left(1-{{\beta }}\right){e}_{r}^{u}-{e}_{r}^{a}\right)\times {u}_{r}\right]\\ \begin{array}{c}=\left(1-{{\beta }}\right)E\left[{e}_{r}^{u}\times {u}_{r}\right]\\ =\left(1-{{\beta }}\right){Var}\left({e}_{r}^{u}\right)\ne 0.\end{array}\end{array}\end{array}\end{array}\end{array}$$

Split sampling solves this bias by using separate patient cases for computing unassisted error and treatment effect. A simple construction of split sampling is to use a separate case *i* for computing the treatment effect and using the remaining cases to compute unassisted error. With this construction, we obtain the following estimate of ${{\beta }}$:

13 $$\frac{{Cov}\left({u}_{i,r}-{a}_{i,r},{u}_{\ne i,r}\right)}{{Var}\left({u}_{\ne i,r}\right)}$$

where ${u}_{i,r}$ is the unassisted performance on case *i* for radiologist $r$, and ${u}_{\ne i,r}$ is the mean unassisted performance computed on all unassisted cases other than *i*. If the errors on each case used to compute ${u}_{r}^{* }$ and ${a}_{r}^{* }$ are independent, the estimate of ${{\beta }}$ is equal to

14 $$\frac{{Cov}\left({u}_{r}^{* }-{a}_{r}^{* },{u}_{r}^{* }\right)}{{Var}\left({u}_{\ne i,r}\right)}$$

The remaining discrepancy in the denominator again represents attenuation bias and is addressed in a later subsection.

### Data efficient split sampling construction

To study unassisted error as a predictor of treatment effect, we built a linear regression model with the following formulation using the statsmodels library: *treatment effect* ∼ *1* *+* *unassisted error*. We designed the following split sampling construction to maximize data efficiency when computing the independent and dependent variables in the linear regression.

Let *i* index a patient case and $r$ index a radiologist. Assume that a radiologist reads ${N}_{u}$ cases unassisted and ${N}_{a}$ cases assisted. Recall that the unassisted and assisted cases are disjoint for the non-repeated-measure data; they overlap exactly for the repeated-measure data.

For the non-repeated-measure design, we adopt the following construction:

15 $${u}_{i,r}-{a}_{r}={{\beta }}{x}_{\ne i,r}+{{\rm{\varepsilon }}}_{{u}_{i,r}}+{{\rm{\varepsilon }}}_{{a}_{r}}$$

where ${x}_{\ne i,r}=\frac{1}{{N}_{u}-1}{\sum }_{k\ne i}{u}_{k,r}$ and ${a}_{r}=\frac{1}{{N}_{a}}{\sum }_{k}{a}_{k,r}$. Here, ${x}_{\ne i,r}$ is the mean unassisted performance computed on all unassisted cases other than *i*; ${u}_{{i},{r}}$ is the unassisted performance on case *i* for radiologist $r$; and ${a}_{r}$ is the mean assisted performance on all assisted cases for radiologist $r$.

For the repeated-measure design, we adopt the following construction:

16 $${u}_{i,r}-{a}_{i,r}={{\beta }}{x}_{\ne i,r}+{{\rm{\varepsilon }}}_{{u}_{i,r}}+{{\rm{\varepsilon }}}_{{a}_{i,r}}$$

where ${x}_{\ne i,r}=\frac{1}{{N}_{u}-1}{\sum }_{k\ne i}{u}_{k,r}$. Here, ${x}_{\ne i,r}$ is the mean unassisted performance computed on all cases other than *i*; ${u}_{i,r}$ is the unassisted performance on case *i* for radiologist $r$; and ${a}_{i,r}$ is the assisted performance on case *i* for radiologist $r$.

To study unassisted error as a predictor of assisted error, we built a linear regression model with the following formulation using the statsmodels library: *assisted error* ∼ *1* *+* *unassisted error*. We designed the following split sampling construction that maximizes data efficiency when computing the independent and dependent variables in the linear regression.

For the non-repeated-measure design, we adopt the following construction:

17 $${a}_{i,r}={{\beta }}{x}_{r}+{{\rm{\varepsilon }}}_{i,r}$$

where ${x}_{r}=\frac{1}{{N}_{u}}{\sum }_{k}\,{x}_{k,r}$. Here, ${x}_{r}$ is the mean unassisted performance computed on all unassisted cases, and ${a}_{i,r}$ is the assisted performance on case *i* for radiologist $r$.

For the repeated-measure design, we adopt the following construction:

18 $${a}_{i,r}={{\beta }}{x}_{\ne i,r}+{{\rm{\varepsilon }}}_{i,r}$$

where ${x}_{\ne i,r}=\frac{1}{{N}_{u}-1}{\sum }_{k\ne i}{u}_{k,r}$. Here, ${x}_{\ne i,r}$ is the mean unassisted performance computed on all unassisted cases other than *i* and ${a}_{i,r}$ is the assisted performance on case *i* for radiologist $r$.

The constructions above again emphasize the necessity for split sampling. Without split sampling, the mean unassisted performance, which is the independent variable of the linear regression, will be correlated with the error terms due to overlapping patient cases, leading to a bias in the regression.

### Adjustment for attenuation bias

We adjusted for attenuation bias for the split sampling linear regression formulations.

We want to estimate regressions of the form

19 $${Y}_{r}={{{\beta }}}_{0}+{{{\beta }}}_{1}E\left[{x}_{r}\right]+{{\rm{\varepsilon }}}_{r}$$

where ${Y}_{r}$ is an outcome for radiologist $r$ and $E\left[{x}_{r}\right]$ is radiologist $r$ʼs average unassisted performance. We observe

20 $$\widetilde{{x}}_{r}=\frac{1}{{N}_{r}}\mathop{\sum }\limits_{i}{x}_{{ir}}=E\left[{x}_{r}\right]+{{{\eta }}}_{r}$$

where ${{{\eta }}}_{r}=\frac{1}{{N}_{r}}\mathop{\sum }\limits_{i}{x}_{{ir}}-E\left[{x}_{r}\right]$ and $E\left[{{{\eta }}}_{r}{x}_{r}\right]=0$ and $E\left[{{{\eta }}}_{r}{{\rm{\varepsilon }}}_{r}\right]=0$, which are justified by independent and identically distributed (i.i.d.) sampling of cases and split sampling, respectively.

Using observations from the experiment, we estimate the following regression:

21 $${Y}_{r}={{\rm{\gamma }}}_{0}+{{\rm{\gamma }}}_{1}\tilde{x}_{r}+{{\rm{\varepsilon }}}_{r}$$

Recall that

22 $$\begin{array}{rcl}{{\hat{\rm{\gamma }}}_{1}}{\to }^{p}\frac{E\left[\left({x}_{r}+{{{\eta }}}_{r}-E\left[{x}_{r}\right]\right)\left({Y}_{r}-E\left[{Y}_{r}\right]\right)\right]}{E\left[{\left({x}_{r}+{{{\eta }}}_{r}-E\left[{x}_{r}\right]\right)}^{2}\right]} =\\ \frac{E\left[\left({x}_{r}-E\left[{x}_{r}\right]\right)\left({Y}_{r}-E\left[{Y}_{r}\right]\right)\right]}{E\left[{\left({x}_{r}-E\left[{x}_{r}\right]\right)}^{2}\right]+E\left[{{{\eta }}}_{r}^{2}\right]}={{{\beta }}}_{1}{\rm{\lambda }}\end{array}$$

where ${\rm{\lambda }}=\frac{E\left[{\left({x}_{r}-E\left[{x}_{r}\right]\right)}^{2}\right]}{E\left[{\left({x}_{r}-E\left[{x}_{r}\right]\right)}^{2}\right]+E\left[{{{\eta }}}_{r}^{2}\right]}$ and ${{{\beta }}}_{1}=\frac{E\left[\left({x}_{r}-E\left[{x}_{r}\right]\right)\left({Y}_{r}-E\left[{Y}_{r}\right]\right)\right]}{E\left[{\left({x}_{r}-E\left[{x}_{r}\right]\right)}^{2}\right]}$. We can estimate ${\rm{\lambda }}$ using a plug-in estimator for each term in the data: (1)

23 $$\begin{array}{rcl}E\left[{{{\eta }}}_{r}^{2}\right]=E\left[{\left(\frac{1}{{N}_{r}}\mathop{\sum }\limits_{i}{x}_{{ir}}-E\left[{x}_{{ir}}\right]\right)}^{2}\right]\\=E\left[\frac{{\sum }_{i}{\left({x}_{{ir}}-E\left[{x}_{{ir}}\right]\right)}^{2}}{{N}_{r}}\right]=E\left[s.e.{\left(\tilde{x}_{r}\right)}^{2}\right].\end{array}$$

This is the standard error of the mean estimator. (2)

24 $$E\left[{\left({x}_{r}-E\left[{x}_{r}\right]\right)}^{2}\right]=E\left[{\left(\tilde{x}_{r}-E\left[\tilde{x}_{r}\right]\right)}^{2}\right]-E\left[{{{\eta }}}_{r}^{2}\right],$$

which can be estimated by taking the difference between the variance of the observed $\widetilde{{x}}_{r}$’s and the estimated $E\left[{{{\eta }}}_{r}^{2}\right]$. The denominator of ${\rm{\lambda }}$ is effectively $E\left[{\left(\tilde{x}_{r}-E\left[\tilde{x}_{r}\right]\right)}^{2}\right]$.

Finally, with $\hat{{\rm{\lambda }}}$, we can estimate ${{{\beta }}}_{1}$ using the estimator

25 $${{{\hat{\beta }}}_{1}}={{\hat{\rm{\gamma }}}_{1}}/{\hat{\rm{\lambda }}}.$$

For inference, notice that $\sqrt{n}\left({{\hat{\rm{\gamma }}}_{1}}-{{\rm{\gamma }}}_{1}\right){\to }^{d}N\left(0,{{\rm{\sigma }}}_{{\rm{\gamma }}}^{2}\right)$ and $\hat{{\rm{\lambda }}}{\to }^{p}\,{\rm{\lambda }}$. By Slutsky’s theorem, we know that

26 $$\sqrt{n}\frac{\left(\hat{{\rm{\gamma }}}-{\rm{\gamma }}\right)}{\hat{{\rm{\lambda }}}}{\to }^{d}N\left(0,\frac{{{\rm{\sigma }}}_{{\rm{\gamma }}}^{2}}{{{\rm{\lambda }}}^{2}}\right).$$

Therefore, we divide the standard errors of ${{\hat{\rm{\gamma }}}_{1}}$ by $\hat{{\rm{\lambda }}}$ to obtain the standard errors of ${{{\hat{\beta }}}_{1}}$.

This concludes the adjustment for attenuation bias for the slope term.

### Statistical testing

To determine the amount of heterogeneity between subgroups of radiologists receiving lower versus higher treatment effects, we ran an unpaired *t*-test between the two subgroups of treatment effects computed using the empirical Bayes method. We used the Wald test to test regression coefficients against the null hypothesis of joint equality among treatment effects of different subgroups to determine if there is a statistically significant difference among subgroups split based on the predictor of interest. We also used the Wald test to test regression coefficients against the null hypothesis of zero to determine in a continuous analysis if the independent variable, namely unassisted error, is a predictor of the dependent variable, namely treatment effect or assisted error. We used the Benjamini–Hochberg procedure to correct for multiple hypothesis testing over 15 individual pathologies. For the analysis on treatment effect on AUROC between subgroups determined by AI error (Supplementary Table 34), we conducted an *F*-test to determine whether there is a statistically significant difference between treatment effects on AUROC in different bins. Specifically, we used the number of reads that fall into each bin as the group size. We used the grand mean AUROC and group AUROCs along with group sizes to compute the sum of squares between; we used the estimated standard error of each group AUROC along with the group size to compute the sum of squares within (error).

### Reporting summary

Further information on research design is available in the Nature Portfolio Reporting Summary linked to this article.

## Online content

Any methods, additional references, Nature Portfolio reporting summaries, source data, extended data, supplementary information, acknowledgements, peer review information; details of author contributions and competing interests; and statements of data and code availability are available at 10.1038/s41591-024-02850-w.

### Supplementary information

Supplementary Information Supplementary Tables 1–40, Supplementary Figs. 1 and 2, Supplementary Notes ‘Statistical modeling for AUROC analysis’ | ‘Participant recruitment and affiliation’ (contains Supplementary Tables A.1–4) | ‘Experiment interface and instructions’ (contains Supplementary Figs. B.1–7) and Supplementary References

Reporting Summary

Supplementary Video Experiment instructions video presented to participating radiologists

## Data Availability

The 324 patient cases from Stanford University’s healthcare system were used under licensing. They are available at https://stanfordaimi.azurewebsites.net/datasets/5194008e-61cf-4083-9896-3d4bd8bf8b0b, conditioned on a Stanford University data research use agreement. The AI predictions used in the experiment were generated by the CheXpert model trained on the CheXpert dataset^8^, which is publicly available. The clinician–AI collaboration dataset is available at https://osf.io/z7apq/ upon request for access at the Open Science Framework dataset page.

## Code availability

Code for the analysis is available at 10.5281/zenodo.10467492 (ref. ^46^). Data analysis was conducted using Python 3.9.7, libraries statsmodels 0.13.5 and scipy 1.10.1; and R 4.1.3 and libraries MRMCaov 0.3.0 and auctestr 1.0.0.
