# Supplementary material for: Heterogeneity and predictors of the effects of AI assistance on radiologists
Source: Nat Med. 2024 Mar 19;30(3):837–49. doi: 10.1038/s41591-024-02850-w (PMC10957478; doi:10.1038/s41591-024-02850-w)
Supplement: Supplementary file 1 — Supplementary Tables 1–40, Supplementary Figs. 1 and 2, Supplementary Notes ‘Statistical modeling for AUROC analysis’ | ‘Participant recruitment and affiliation’ (contains Supplementary Tables A.1–4) | ‘Experiment interface and instructions’ (contains Supplementary Figs. B.1–7) and Supplementary References [file 41591_2024_2850_MOESM1_ESM.pdf]

nature medicine

Article

https://doi.org/10.1038/s41591-024-02850-w

Heterogeneity and predictors of the effects of AI assistance on radiologists

In the format provided by the authors and unedited

Supplementary information

Supplementary Table 1 | Ranges of heterogeneous unassisted errors and treatment effects on unassisted error of radiologists. The ranges of heterogeneous unassisted errors and treatment effects on unassisted error of 140 radiologists on individual pathologies as computed using the empirical Bayes method. Uncomputable results due to dataset size and statistical restrictions are marked by U (additional explanation for the causes is available in the Methods section). Non-applicable results are marked by N/A.

	Prevalence at 50 probability threshold	Range of unassisted errors	Range of treatment effects on unassisted error
All pathologies aggregated	N/A	6.083 – 14.175, IQR 1.951	-1.295 – 1.440, IQR 0.797
Abnormal	19.44%	12.705 – 48.486, IQR 9.737	-8.914 – 5.563, IQR 3.245
Airspace opacity	16.05%	8.568 – 34.866, IQR 3.888	-3.204 – 2.043, IQR 1.230
Atelectasis	11.11%	6.798 – 15.022, IQR 1.145	-2.794 – 4.510, IQR 1.412
Bacterial/lobar pneumonia	1.23%	0.014 – 8.471, IQR 1.553	-0.454 – 0.675, IQR 0.238
Cardiomediastinal abnormality	13.27%	7.791 – 33.421, IQR 5.102	-1.125 – 4.050, IQR 1.247
Cardiomegaly	4.32%	4.145 – 30.272, IQR 5.570	-2.818 – 6.230, IQR 2.390
Consolidation	3.40%	0.603 – 11.093, IQR 2.195	U
Edema	1.85%	0.387 – 22.336, IQR 5.556	-6.837 – 1.285, IQR 2.134
Lesion	0.00%	0.345 – 7.733, IQR 1.881	-1.048 – 0.920, IQR 0.442
Pleural effusion	3.70%	0.000 – 6.885, IQR 1.439	U
Pleural other	0.00%	0.000 – 1.849, IQR 0.554	U
Pneumothorax	0.31%	0.000 – 1.204, IQR 0.647	U
Rib fracture	0.93%	0.000 – 4.201, IQR 1.254	U
Shoulder fracture	0.00%	0.000 – 1.555, IQR 0.749	U
Support device hardware	17.59%	2.186 – 41.434, IQR 6.638	-3.545 – 6.113, IQR 2.797

Supplementary Table 2 | Heterogeneous treatment effects of radiologists in binary subgroups. The treatment effects, 95% confidence intervals and p-values of 136 radiologists that have survey data on individual pathologies in binary subgroups split based on treatment effect. A two-sided, unpaired t-test between the two subgroups of treatment effects was conducted. The Benjamini-Hochberg procedure was used to correct for multiple hypothesis testing over 15 individual pathologies. The difference between subgroups shows the extent of heterogeneity an ideal predictor of treatment effect would have been able to discern. Uncomputable results due to dataset size and statistical restrictions

are marked by U (additional explanation for the causes is available in the Methods section). Non-applicable results are marked by N/A.

	Lower subgroup	Higher subgroup	P-value for testing against subgroup equality	Benjamini-Hochberg adjusted p-value
All pathologies aggregated	-0.357 (95% CI: -0.429 – -0.284)	0.472 (95% CI: 0.403 – 0.541)	3.50e-34, < 0.001	N/A
Abnormal	-3.683 (95% CI: -4.132 – -3.234)	0.511 (95% CI: 0.164 – 0.858)	7.76e-30, < 0.001	1.66e-29, < 0.001
Airspace opacity	-1.360 (95% CI: -1.506 – -1.215)	0.105 (95% CI: -0.036 – 0.245)	3.84e-29, < 0.001	7.20e-29, < 0.001
Atelectasis	-0.193 (95% CI: -0.349 – -0.038)	1.572 (95% CI: 1.404 – 1.741)	2.21e-31, < 0.001	1.10e-30, < 0.001
Bacterial/lobar pneumonia	-0.047 (95% CI: -0.078 – -0.016)	0.263 (95% CI: 0.231 – 0.294)	3.84e-28, < 0.001	6.40e-28, < 0.001
Cardiomediastinal abnormality	0.579 (95% CI: 0.422 – 0.735)	2.150 (95% CI: 2.011 – 2.288)	1.94e-30, < 0.001	4.85e-30, < 0.001
Cardiomegaly	-0.359 (95% CI: -0.606 – -0.113)	2.468 (95% CI: 2.193 – 2.742)	3.82e-31, < 0.001	1.43e-30, < 0.001
Consolidation	0.235 (95% CI: 0.233 – 0.238)	0.245 (95% CI: 0.244 – 0.246)	1.47e-10, < 0.001	2.21e-10, < 0.001
Edema	-4.231 (95% CI: -4.474 – -3.988)	-1.578 (95% CI: -1.814 – -1.341)	5.60e-32, < 0.001	8.40e-31, < 0.001
Lesion	-0.063 (95% CI: -0.118 – -0.008)	0.445 (95% CI: 0.408 – 0.482)	4.15e-31, < 0.001	1.25e-30, < 0.001
Pleural effusion	U	U	U	U
Pleural other	U	U	U	U
Pneumothorax	U	U	U	U
Rib fracture	U	U	U	U
Shoulder fracture	U	U	U	U
Support device hardware	-0.452 (95% CI: -0.727 – -0.176)	2.698 (95% CI: 2.396 – 3.000)	2.09e-31, < 0.001	1.57e-30, < 0.001

Supplementary Table 3 | Treatment effects of subgroups split based on combined characteristics. The treatment effects, 95% confidence intervals and p-values of radiologists in binary subgroups split based on combined characteristics of years of experience, subspecialty in thoracic radiology and experience with AI tools. The Wald test was used to test regression coefficients that estimate treatment effects against the null hypothesis of joint equality among treatment effects of different subgroups. Details of the statistical models are available in Methods. The Benjamini-Hochberg

procedure was used to correct for multiple hypothesis testing over 15 individual pathologies. Non-applicable results are marked by N/A.

	Lower subgroup	Higher subgroup	P-value for testing against subgroup equality	Benjamini-Hochberg adjusted p-value
All pathologies aggregated	0.091 (95% CI: -0.231 – 0.413)	0.070 (95% CI: -0.243 – 0.383)	0.917	N/A
Abnormal	-1.198 (95% CI: -2.360 – -0.037)	-1.476 (95% CI: -3.345 – 0.394)	0.795	0.795
Airspace opacity	-0.348 (95% CI: -1.258 – 0.562)	-0.884 (95% CI: -2.187 – 0.420)	0.406	0.677
Atelectasis	1.058 (95% CI: 0.003 – 2.113)	0.601 (95% CI: -0.143 – 1.346)	0.459	0.689
Bacterial/lobar pneumonia	0.343 (95% CI: -0.144 – 0.829)	-0.099 (95% CI: -0.695 – 0.498)	0.249	0.748
Cardiomediastinal abnormality	1.320 (95% CI: 0.243 – 2.397)	1.077 (95% CI: 0.284 – 1.870)	0.698	0.748
Cardiomegaly	1.424 (95% CI: 0.314 – 2.534)	0.836 (95% CI: -0.068 – 1.741)	0.383	0.719
Consolidation	0.390 (95% CI: -0.150 – 0.929)	-0.032 (95% CI: -0.755 – 0.691)	0.336	0.720
Edema	-2.718 (95% CI: -3.686 – -1.751)	-2.419 (95% CI: -3.835 – -1.002)	0.696	0.803
Lesion	0.548 (95% CI: 0.143 – 0.953)	0.136 (95% CI: -0.312 – 0.584)	0.146	0.731
Pleural effusion	0.762 (95% CI: 0.057 – 1.466)	0.296 (95% CI: -0.365 – 0.957)	0.323	0.807
Pleural other	0.307 (95% CI: 0.031 – 0.582)	0.099 (95% CI: -0.043 – 0.240)	0.180	0.676
Pneumothorax	0.259 (95% CI: 0.050 – 0.468)	-0.161 (95% CI: -0.516 – 0.195)	0.105	0.787
Rib fracture	-0.295 (95% CI: -0.868 – 0.279)	0.314 (95% CI: -0.134 – 0.762)	0.055	0.829
Shoulder fracture	0.066 (95% CI: -0.298 – 0.429)	0.193 (95% CI: -0.090 – 0.476)	0.623	0.850
Support device hardware	1.124 (95% CI: 0.008 – 2.239)	0.769 (95% CI: -0.340 – 1.878)	0.633	0.791

Supplementary Table 4 | Treatment effects of subgroups split based on years of experience. The treatment effects, 95% confidence intervals and p-values of radiologists in binary subgroups split based on years of experience. The Wald test was used to test regression coefficients that estimate

treatment effects against the null hypothesis of joint equality among treatment effects of different subgroups. Details of the statistical models are available in Methods. The Benjamini-Hochberg procedure was used to correct for multiple hypothesis testing over 15 individual pathologies. Non-applicable results are marked by N/A.

	Subgroup of less than or equal to 6 years of experience	Subgroup of more than 6 years of experience	P-value for testing against subgroup equality	Benjamini-Hochberg adjusted p-value
All pathologies aggregated	0.204 (95% CI: -0.209 – 0.616)	-0.002 (95% CI: -0.266 – 0.263)	0.381	N/A
Abnormal	-1.084 (95% CI: -2.651 – 0.483)	-1.390 (95% CI: -2.621 – -0.160)	0.747	1.121
Airspace opacity	-0.274 (95% CI: -1.643 – 1.094)	-0.745 (95% CI: -1.657 - 0.168)	0.507	0.951
Atelectasis	1.014 (95% CI: -0.070 – 2.098)	0.511 (95% CI: -0.215 – 1.237)	0.427	2.134
Bacterial/lobar pneumonia	0.170 (95% CI: -0.449 – 0.789)	0.184 (95% CI: -0.294 – 0.662)	0.971	0.971
Cardiomediastinal abnormality	1.214 (95% CI: 0.034 – 2.394)	1.131 (95% CI: 0.367 – 1.894)	0.904	1.043
Cardiomegaly	1.153 (95% CI: -0.194 – 2.500)	1.095 (95% CI: 0.315 – 1.875)	0.938	1.005
Consolidation	0.312 (95% CI: -0.407 – 1.030)	0.180 (95% CI: -0.347 – 0.708)	0.756	1.030
Edema	-2.903 (95% CI: -4.145 – -1.660)	-2.460 (95% CI: -3.431 – -1.488)	0.500	1.072
Lesion	0.244 (95% CI: -0.261 – 0.750)	0.452 (95% CI: 0.099 – 0.805)	0.462	1.155
Pleural effusion	0.633 (95% CI: -0.202 – 1.468)	0.402 (95% CI: -0.130 – 0.934)	0.620	1.033
Pleural other	0.077 (95% CI: -0.058 – 0.212)	0.153 (95% CI: -0.026 – 0.333)	0.457	1.714
Pneumothorax	0.103 (95% CI: -0.070 – 0.276)	-0.001 (95% CI: -0.222 – 0.220)	0.461	1.384
Rib fracture	0.388 (95% CI: -0.213 – 0.989)	-0.003 (95% CI: -0.470 – 0.465)	0.267	2.002
Shoulder fracture	0.183 (95% CI: -0.130 – 0.496)	0.157 (95% CI: -0.129 – 0.442)	0.898	1.123
Support device hardware	1.828 (95% CI: 0.391 – 3.264)	0.309 (95% CI: -0.678 – 1.297)	0.085	1.268

Supplementary Table 5 | Treatment effects of subgroups split based on subspecialty in thoracic radiology. The treatment effects, 95% confidence intervals and p-values of radiologists in binary subgroups split based on subspecialty in thoracic radiology. The Wald test was used to test regression coefficients that estimate treatment effects against the null hypothesis of joint equality among treatment effects of different subgroups. Details of the statistical models are available in Methods. The Benjamini-Hochberg procedure was used to correct for multiple hypothesis testing over 15 individual pathologies. Non-applicable results are marked by N/A.

	Subgroup of radiologists that do not specialize in thoracic radiology	Subgroup of radiologists that specialize in thoracic radiology	P-value for testing against subgroup equality	Benjamini-Hochberg adjusted p-value
All pathologies aggregated	0.066 (95% CI: -0.216 – 0.347)	0.121 (95% CI: -0.269 – 0.511)	0.808	N/A
Abnormal	-1.102 (95% CI: -2.265 – 0.061)	-1.729 (95% CI: -3.529 – 0.071)	0.543	1.164
Airspace opacity	-0.385 (95% CI: -1.373 – 0.603)	-1.034 (95% CI: -2.195 – 0.127)	0.301	1.127
Atelectasis	0.685 (95% CI: -0.050 – 1.420)	0.787 (95% CI: -0.240 – 1.813)	0.862	0.995
Bacterial/lobar pneumonia	0.106 (95% CI: -0.339 – 0.551)	0.380 (95% CI: -0.455 – 1.214)	0.575	1.078
Cardiomediastinal abnormality	1.184 (95% CI: 0.399 – 1.969)	1.108 (95% CI: -0.066 – 2.283)	0.912	0.977
Cardiomegaly	1.077 (95% CI: 0.239 – 1.915)	1.232 (95% CI: -0.055 – 2.519)	0.825	1.125
Consolidation	0.112 (95% CI: -0.415 – 0.639)	0.568 (95% CI: -0.342 – 1.478)	0.397	1.190
Edema	-2.674 (95% CI: -3.676 – -1.673)	-2.530 (95% CI: -3.723 – -1.338)	0.829	1.036
Lesion	0.409 (95% CI: 0.035 – 0.782)	0.259 (95% CI: -0.353 – 0.870)	0.679	1.132
Pleural effusion	0.450 (95% CI: -0.088 – 0.989)	0.617 (95% CI: -0.258 – 1.492)	0.725	1.088
Pleural other	0.124 (95% CI: -0.032 – 0.279)	0.120 (95% CI: -0.076 – 0.316)	0.973	0.973
Pneumothorax	-0.038 (95% CI: -0.243 – 0.168)	0.258 (95% CI: 0.026 – 0.489)	0.093	0.698
Rib fracture	-0.001 (95% CI: -0.462 – 0.459)	0.583 (95% CI: -0.058 – 1.223)	0.123	0.615
Shoulder fracture	0.277 (95% CI: -0.025 – 0.579)	-0.138 (95% CI: -0.428 – 0.152)	0.079	1.189
Support device hardware	0.762 (95% CI: -0.254 – 1.779)	1.341 (95% CI: -0.056 – 2.738)	0.502	1.255

Supplementary Table 6 | Treatment effects of subgroups split based on experience with AI tools. The treatment effects, 95% confidence intervals and p-values of radiologists in binary subgroups split based on experience with AI tools. The Wald test was used to test regression coefficients that estimate treatment effects against the null hypothesis of joint equality among treatment effects of different subgroups. Details of the statistical models are available in Methods. The Benjamini-Hochberg procedure was used to correct for multiple hypothesis testing over 15 individual pathologies. Non-applicable results are marked by N/A.

	Subgroup of radiologists that do not have experience with AI tools	Subgroup of radiologists that have experience with AI tools	P-value for testing against subgroup equality	Benjamini-Hochberg adjusted p-value
All pathologies aggregated	-0.057 (95% CI: -0.562 – 0.449)	0.138 (95% CI: -0.106 – 0.382)	0.472	N/A
Abnormal	-2.442 (95% CI: -4.300 – -0.584)	-0.775 (95% CI: -1.876 – 0.326)	0.106	0.796
Airspace opacity	-0.706 (95% CI: -2.220 – 0.809)	-0.495 (95% CI: -1.375 – 0.386)	0.775	0.969
Atelectasis	0.611 (95% CI: -0.585 – 1.806)	0.754 (95% CI: 0.056 – 1.453)	0.827	0.886
Bacterial/lobar pneumonia	0.276 (95% CI: -0.489 – 1.040)	0.138 (95% CI: -0.299 – 0.574)	0.754	1.028
Cardiomediastinal abnormality	1.494 (95% CI: 0.108 – 2.879)	1.025 (95% CI: 0.287 – 1.764)	0.548	1.174
Cardiomegaly	0.848 (95% CI: -0.875 – 2.570)	1.232 (95% CI: 0.482 – 1.982)	0.678	1.131
Consolidation	0.315 (95% CI: -0.533 – 1.163)	0.198 (95% CI: -0.302 – 0.698)	0.805	0.929
Edema	-3.923 (95% CI: -5.345 – -2.502)	-2.095 (95% CI: -2.982 – -1.208)	0.009	0.140
Lesion	0.218 (95% CI: -0.417 – 0.853)	0.433 (95% CI: 0.091 – 0.774)	0.539	1.347
Pleural effusion	0.247 (95% CI: -0.796 – 1.289)	0.598 (95% CI: 0.086 – 1.110)	0.537	1.611
Pleural other	0.196 (95% CI: -0.011 – 0.403)	0.092 (95% CI: -0.065 – 0.249)	0.416	1.561
Pneumothorax	-0.011 (95% CI: -0.228 – 0.206)	0.062 (95% CI: -0.135 - 0.260)	0.631	1.183
Rib fracture	0.194 (95% CI: -0.610 – 0.998)	0.136 (95% CI: -0.305 – 0.577)	0.899	0.899
Shoulder fracture	0.212 (95% CI: -0.158 – 0.582)	0.148 (95% CI: -0.084 – 0.381)	0.740	1.111

Support device hardware	1.624 (95% CI: -0.410 – 3.658)	0.618 (95% CI: -0.116 – 1.351)	0.339	1.694

Supplementary Table 7 | Regression results for treatment effect vs. unassisted error. The regression coefficients, adjustment for attenuation bias and p-values of the analysis on the relationship between treatment effect and unassisted error. The Wald test was used to test regression coefficients against the null hypothesis of zero to determine in a continuous analysis if the independent variable is a predictor of the dependent variable. Details of the statistical models are available in Methods. The Benjamini-Hochberg procedure was used to correct for multiple hypothesis testing over 15 individual pathologies. Non-applicable results are marked by N/A.

	Unassisted error coefficient before adjustment for attenuation bias	Intercept coefficient before adjustment for attenuation bias	Unassisted error coefficient after adjustment for attenuation bias	P-value for testing unassisted error coefficient against 0	Benjamini-Hochberg adjusted p-value
All pathologies aggregated	0.066 (95% CI: -0.083 – 0.215)	-0.572 (95% CI: -1.923 – 0.780)	0.077 (95% CI: -0.097 – 0.251)	0.385	N/A
Abnormal	0.149 (95% CI: 0.045 – 0.253)	-6.088 (95% CI: -9.487 – -2.689)	0.179 (95% CI: 0.054 – 0.304)	0.005	0.038
Airspace opacity	0.111 (95% CI: -0.020 – 0.243)	-2.549 (95% CI: -4.891 – -0.208)	0.182 (95% CI: -0.033 – 0.397)	0.097	0.363
Atelectasis	0.272 (95% CI: 0.051 – 0.492)	-2.495 (95% CI: -4.961 – -0.029)	0.901 (95% CI: 0.170 – 1.633)	0.016	0.079
Bacterial/lobar pneumonia	0.110 (95% CI: -0.037 – 0.257)	-0.410 (95% CI: -1.186 – 0.366)	0.220 (95% CI: -0.074 – 0.515)	0.143	0.357
Cardiomediastinal abnormality	0.013 (95% CI: -0.162 – 0.188)	1.034 (95% CI: -2.010 – 4.077)	0.019 (95% CI: -0.230 – 0.267)	0.882	0.945
Cardiomegaly	0.122 (95% CI: -0.122 – 0.366)	-0.619 (95% CI: -3.741 – 2.503)	0.164 (95% CI: -0.164 – 0.492)	0.327	0.545
Consolidation	0.106 (95% CI: -0.046 – 0.258)	-0.521 (95% CI: -1.628 – 0.586)	0.206 (95% CI: -0.090 – 0.502)	0.173	0.370
Edema	-0.001 (95% CI: -0.123 – 0.121)	-3.010 (95% CI: -4.225 – -1.796)	-0.001 (95% CI: -0.153 – 0.150)	0.986	0.986
Lesion	0.157 (95% CI: 0.052 – 0.262)	-0.291 (95% CI: -0.754 – 0.172)	0.220 (95% CI: 0.073 – 0.368)	0.003	0.052
Pleural effusion	0.064 (95% CI: -0.156 – 0.285)	0.129 (95% CI: -0.959 – 1.217)	0.191 (95% CI: -0.464 – 0.847)	0.567	0.773
Pleural other	-0.023 (95% CI: -0.232 – 0.185)	0.173 (95% CI: 0.014 – 0.331)	-0.048 (95% CI: -0.477 – 0.382)	0.827	0.954
Pneumothorax	0.030 (95% CI: -0.184 – 0.243)	-0.033 (95% CI: -0.354 – 0.287)	0.096 (95% CI: -0.594 – 0.786)	0.784	0.981
Rib fracture	0.085 (95% CI:	-0.049 (95% CI:	0.317 (95% CI:	0.359	0.539

	-0.096 – 0.266)	-0.696 – 0.598)	-0.361 – 0.994)		
Shoulder fracture	0.112 (95% CI: -0.071 – 0.294)	0.075 (95% CI: -0.190 – 0.340)	0.727 (95% CI: -0.461 – 1.914)	0.230	0.432
Support device hardware	0.149 (95% CI: -0.049 – 0.348)	-0.732 (95% CI: -3.058 – 1.595)	0.185 (95% CI: -0.061 – 0.431)	0.141	0.422

Supplementary Table 8 | Regression results for treatment effect vs. unassisted error without split sampling. The regression coefficients, adjustment for attenuation bias and p-values of the hypothetical analysis on the relationship between treatment effect and unassisted error without split sampling. The Wald test was used to test regression coefficients against the null hypothesis of zero to determine in a continuous analysis if the independent variable is a predictor of the dependent variable. Details of the statistical models are available in Methods. The Benjamini-Hochberg procedure was used to correct for multiple hypothesis testing over 15 individual pathologies. Non-applicable results are marked by N/A.

	Unassisted error coefficient before adjustment for attenuation bias	Intercept coefficient before adjustment for attenuation bias	Unassisted error coefficient after adjustment for attenuation bias	P-value for testing unassisted error coefficient against 0	Benjamini-Hochberg adjusted p-value
All pathologies aggregated	0.309 (95% CI: 0.155 – 0.463)	-2.831 (95% CI: -4.203 – -1.459)	0.357 (95% CI: 0.179 – 0.536)	8.46e-5, < 0.001	N/A
Abnormal	0.285 (95% CI: 0.184 – 0.386)	-10.299 (95% CI: -13.568 – -7.031)	0.340 (95% CI: 0.219 – 0.460)	3.53e-8, < 0.001	5.89e-8, < 0.001
Airspace opacity	0.452 (95% CI: 0.316 – 0.589)	-8.339 (95% CI: -10.688 – -5.990)	0.732 (95% CI: 0.511 – 0.953)	8.67e-11, < 0.001	2.17e-10, < 0.001
Atelectasis	0.927 (95% CI: 0.761 – 1.092)	-10.087 (95% CI: -11.951 – -8.223)	3.145 (95% CI: 2.584 – 3.706)	4.15e-28, < 0.001	6.23e-27, < 0.001
Bacterial/lobar pneumonia	0.567 (95% CI: 0.440 – 0.694)	-2.665 (95% CI: -3.311 – -2.018)	1.132 (95% CI: 0.879 – 1.386)	2.12e-18, < 0.001	1.06e-17, < 0.001
Cardiomediastinal abnormality	0.276 (95% CI: 0.126 – 0.426)	-3.847 (95% CI: -6.360 – -1.335)	0.384 (95% CI: 0.175 – 0.593)	3.18e-4, < 0.001	4.34e-4, < 0.001
Cardiomegaly	0.347 (95% CI: 0.127 – 0.566)	-3.768 (95% CI: -6.529 – -1.008)	0.457 (95% CI: 0.167 – 0.746)	0.002	0.002
Consolidation	0.552 (95% CI: 0.420 – 0.685)	-3.633 (95% CI: -4.545 – -2.721)	1.063 (95% CI: 0.808 – 1.318)	3.30e-16, < 0.001	1.24e-15, < 0.001
Edema	0.149 (95% CI: 0.029 – 0.269)	-4.352 (95% CI: -5.565 – -3.139)	0.185 (95% CI: 0.036 – 0.334)	0.015	0.016
Lesion	0.450 (95% CI: 0.207 – 0.693)	-1.405 (95% CI: -2.272 – -0.537)	0.641 (95% CI: 0.295 – 0.987)	2.85e-4, < 0.001	4.27e-4, < 0.001
Pleural effusion	0.624 (95% CI: 0.415 – 0.833)	-2.408 (95% CI: -3.315 – -1.502)	1.803 (95% CI: 1.200 – 2.407)	4.54e-9, < 0.001	8.51e-9, < 0.001

Pleural other	0.391 (95% CI: 0.004 – 0.779)	-0.129 (95% CI: -0.335 – 0.078)	0.797 (95% CI: 0.008 – 1.585)	0.048	0.048
Pneumothorax	0.678 (95% CI: 0.458 – 0.899)	-0.513 (95% CI: -0.793 – -0.233)	2.259 (95% CI: 1.524 – 2.994)	1.70e-9, < 0.001	3.65e-9, < 0.001
Rib fracture	0.652 (95% CI: 0.469 – 0.834)	-1.929 (95% CI: -2.491 – -1.367)	2.163 (95% CI: 1.558 – 2.769)	2.48e-12, < 0.001	7.45e-12, < 0.001
Shoulder fracture	0.888 (95% CI: 0.710 – 1.067)	-0.563 (95% CI: -0.752 – -0.375)	5.600 (95% CI: 4.474 – 6.725)	1.84e-22, < 0.001	1.38e-21, < 0.001
Support device hardware	0.328 (95% CI: 0.126 – 0.530)	-2.999 (95% CI: -5.289 – -0.709)	0.401 (95% CI: 0.154 – 0.648)	0.001	0.002

Supplementary Table 9 | Regression results for assisted error vs. unassisted error. The regression coefficients, adjustment for attenuation bias and p-values of the analysis on the relationship between assisted error and unassisted error. The Wald test was used to test regression coefficients against the null hypothesis of zero to determine in a continuous analysis if the independent variable is a predictor of the dependent variable. Details of the statistical models are available in Methods. The Benjamini-Hochberg procedure was used to correct for multiple hypothesis testing over 15 individual pathologies. Non-applicable results are marked by N/A.

	Unassisted error coefficient before adjustment for attenuation bias	Intercept coefficient before adjustment for attenuation bias	Unassisted error coefficient after adjustment for attenuation bias	P-value for testing unassisted error coefficient against 0	Benjamini-Hochberg adjusted p-value
All pathologies aggregated	0.647 (95% CI: 0.492 – 0.802)	3.243 (95% CI: 1.864 – 4.623)	0.749 (95% CI: 0.570 – 0.929)	3.05e-16, < 0.001	N/A
Abnormal	0.691 (95% CI: 0.590 – 0.792)	11.046 (95% CI: 7.775 – 14.318)	0.824 (95% CI: 0.703 – 0.945)	9.28e-41,< 0.001	1.39e-39, < 0.001
Airspace opacity	0.505 (95% CI: 0.361 – 0.649)	9.065 (95% CI: 6.609 – 11.521)	0.821 (95% CI: 0.586 – 1.055)	7.02-e12, < 0.001	2.63e-11, < 0.001
Atelectasis	-0.034 (95% CI: -0.190 – 0.122)	11.333 (95% CI: 9.558 – 13.107)	-0.117 (95% CI: -0.651 – 0.418)	0.668	0.716
Bacterial/lobar pneumonia	0.377 (95% CI: 0.245 – 0.510)	2.938 (95% CI: 2.279 – 3.598)	0.764 (95% CI: 0.495 – 1.032)	2.40e-8, < 0.001	4.51e-8, < 0.001
Cardiomediastinal abnormality	0.696 (95% CI: 0.546 – 0.845)	4.379 (95% CI: 1.885 – 6.873)	0.969 (95% CI: 0.761 – 1.177)	6.87e-20, < 0.001	3.43e-19, < 0.001
Cardiomegaly	0.626 (95% CI: 0.409 – 0.844)	4.148 (95% CI: 1.424 – 6.872)	0.826 (95% CI: 0.539 – 1.112)	1.60e-8, < 0.001	3.42e-8, < 0.001
Consolidation	0.404 (95% CI: 0.272 – 0.536)	3.937 (95% CI: 3.038 – 4.836)	0.788 (95% CI: 0.531 – 1.045)	1.80e-9, < 0.001	4.50e-09, < 0.001
Edema	0.823 (95% CI: 0.700 – 0.946)	4.608 (95% CI: 3.378 – 5.838)	1.024 (95% CI: 0.871 – 1.177)	2.48e-39, < 0.001	1.86e-38, < 0.001
Lesion	0.522 (95% CI:	1.511 (95% CI: 0.569	0.743 (95% CI: 0.366	1.11e-4, < 0.001	1.85e-4, < 0.001

	0.257 – 0.786)	– 2.453)	– 1.120)		
Pleural effusion	0.279 (95% CI: 0.083 – 0.474)	2.850 (95% CI: 1.985 – 3.715)	0.793 (95% CI: 0.236 – 1.350)	0.005	0.007
Pleural other	0.568 (95% CI: 0.207 – 0.930)	0.158 (95% CI: -0.027 – 0.343)	1.143 (95% CI: 0.416 – 1.871)	0.002	0.003
Pneumothorax	0.192 (95% CI: -0.008 – 0.391)	0.609 (95% CI: 0.331 – 0.888)	0.634 (95% CI: -0.025 – 1.293)	0.059	0.068
Rib fracture	0.209 (95% CI: 0.034 – 0.384)	2.390 (95% CI: 1.822 – 2.959)	0.737 (95% CI: 0.120 – 1.353)	0.019	0.024
Shoulder fracture	0.029 (95% CI: -0.122 – 0.181)	0.631 (95% CI: 0.441 – 0.821)	0.185 (95% CI: -0.773 – 1.143)	0.705	0.705
Support device hardware	0.654 (95% CI: 0.450 – 0.859)	3.221 (95% CI: 0.904 – 5.538)	0.801 (95% CI: 0.550 – 1.052)	3.83e-10, < 0.001	1.15e-9, < 0.001

Supplementary Table 10 | Treatment effects of absolute AI error ranges and overall treatment effect across ranges. The treatment effects, 95% confidence intervals and p-values of different absolute AI error ranges and the statistics for the overall treatment effect across ranges. The Wald test was used to test regression coefficients that estimate treatment effects against the null hypothesis of joint equality among treatment effects of different subgroups. Details of the statistical models are available in Methods. The Benjamini-Hochberg procedure was used to correct for multiple hypothesis testing over 15 individual pathologies. Non-applicable results are marked by N/A.

	AI absolute error in [0, 20]	AI absolute error in (20, 40]	AI absolute error in (40, 60]	AI absolute error in (60, 80]	AI absolute error in (80, 100]	P-value for testing against joint equality	Benjamini-Hochberg adjusted p-value	Across AI absolute error ranges
All pathologies aggregated	0.679 (95% CI: 0.492 – 0.865)	-1.509 (95% CI: -2.267 – -0.750)	-3.556 (95% CI: -4.878 – -2.235)	-6.569 (95% CI: -8.764 – -4.374)	-16.845 (95% CI: -24.288 – -9.403)	3.44e-19, < 0.001	N/A	0.053 (95% CI: -0.181 – 0.286)
Abnormal	2.843 (95% CI: 1.401 – 4.286)	1.023 (95% CI: -0.275 – 2.320)	-1.590 (95% CI: -2.905 – -0.274)	-5.527 (95% CI: -7.593 – -3.461)	-13.663 (95% CI: -19.261 – -8.066)	1.01e-13, < 0.001	3.03e-13, < 0.001	-1.314 (95% CI: -2.290 – -0.337)
Airspace opacity	2.462 (95% CI: 1.706 – 3.218)	-2.585 (95% CI: -3.907 – -1.262)	-8.480 (95% CI: -11.508 – -5.452)	-23.237 (95% CI: -31.001 – -15.474)	N/A	1.33e-23, < 0.001	4.993-23, < 0.001	-0.614 (95% CI: -1.466 – 0.238)
Atelectasis	0.471 (95% CI: -0.001 – 0.942)	1.618 (95% CI: -0.862 – 4.097)	4.736 (95% CI: 0.717 – 8.755)	N/A	N/A	0.084	0.115	0.630 (95% CI: 0.010 – 1.251)
Bacterial/lobar pneumonia	0.168 (95% CI: -0.171 – 0.507)	-1.151 (95% CI: -3.790 – 1.487)	7.584 (95% CI: 6.772 – 8.396)	N/A	N/A	0.00, < 0.001	0.00, < 0.001	0.158 (95% CI: -0.207 – 0.524)
Cardiomediastinal abnormality	1.638 (95% CI: 0.938 – 2.338)	-0.709 (95% CI: -2.364 – 0.946)	-0.721 (95% CI: -4.564 – 3.122)	-5.661 (95% CI: -10.831 – -0.491)	N/A	4.52e-4, < 0.001	8.48e-4, 0.001	1.096 (95% CI: 0.442 – 1.750)
Cardiomegaly	1.688 (95% CI: 1.006 – 2.370)	-2.921 (95% CI: -5.238 – -0.604)	-6.143 (95% CI: -15.214 – 2.927)	N/A	N/A	2.00e-4, < 0.001	4.29e-4, < 0.001	1.076 (95% CI: 0.357 – 1.796)
Consolidation	0.051 (95% CI: -0.379 – 0.481)	1.043 (95% CI: -1.187 – 3.272)	7.811 (95% CI: 3.546 – 12.076)	N/A	N/A	0.003	0.004	0.200 (95% CI: -0.228 – 0.628)
Edema	0.633 (95% CI: 0.104 – 1.163)	-5.360 (95% CI: -6.742 – -3.979)	-11.839 (95% CI: -14.635 – -9.042)	-17.518 (95% CI: -25.434 – -9.602)	-35.218 (95% CI: -36.072 – -34.363)	1.000	1.000	-2.777 (95% CI: -3.646 – -1.908)

Lesion	0.337 (95% CI: 0.046 – 0.629)	0.807 (95% CI: -5.819 – 7.433)	N/A	N/A	N/A	0.889	0.953	0.354 (95% CI: 0.044 – 0.664)
Pleural effusion	0.719 (95% CI: 0.248 – 1.190)	-4.228 (95% CI: -7.668 – -0.788)	-4.872 (95% CI: -6.382 – -3.363)	N/A	N/A	3.55e-9, < 0.001	8.88e-9, < 0.001	0.521 (95% CI: 0.040 – 1.002)
Pleural other	0.134 (95% CI: 0.011 – 0.257)	-2.771 (95% CI: -3.100 – -2.442)	N/A	N/A	N/A	6.28e-41, < 0.001	4.71e-40, < 0.001	0.128 (95% CI: 0.004 – 0.251)
Pneumothorax	0.076 (95% CI: -0.045 – 0.197)	N/A	N/A	-12.541 (95% CI: -14.466 – -10.616)	N/A	5.36e-39, < 0.001	2.68e-38, < 0.001	0.030 (95% CI: -0.128 – 0.188)
Rib fracture	0.184 (95% CI: -0.154 – 0.523)	-0.903 (95% CI: -4.860 – 3.055)	-1.438 (95% CI: -7.187 – 4.310)	1.343 (95% CI: -0.194 – 2.879)	N/A	0.335	0.418	0.180 (95% CI: -0.199 – 0.560)
Shoulder fracture	0.178 (95% CI: -0.027 – 0.384)	0.306 (95% CI: -0.262 – 0.875)	N/A	N/A	N/A	0.686	0.791	0.182 (95% CI: -0.017 – 0.381)
Support device hardware	1.707 (95% CI: 0.970 – 2.443)	0.071 (95% CI: -2.294 – 2.436)	-3.936 (95% CI: -8.228 – 0.355)	-2.505 (95% CI: -9.717 – 4.708)	N/A	0.024	0.036	0.940 (95% CI: 0.114 – 1.767)

Supplementary Table 11 | Sample sizes and percentages of absolute AI error ranges. The sample sizes and percentages of radiologist predictions that fall into different absolute AI error ranges for Supplementary Table 10 and Fig. 4.

	AI absolute error in [0, 20]	AI absolute error in (20, 40]	AI absolute error in (40, 60]	AI absolute error in (60, 80]	AI absolute error in (80, 100]
All pathologies aggregated	81.9% (n = 176,130)	11.1% (n = 23,827)	5.0% (n = 10,753)	1.9% (n = 4,019)	0.2% (n = 371)
Abnormal	13.9% (n = 2,000)	23.8% (n = 3,413)	38.4% (n = 5,501)	21.7% (n = 3,110)	2.2% (n = 316)
Airspace opacity	53.6% (n = 7,688)	36.5% (n = 5,227)	8.9% (n = 1,280)	1.0% (n = 145)	0.0% (n = 0)
Atelectasis	82.3% (n = 11,804)	12.9% (n = 1,851)	4.8% (n = 685)	0.0% (n = 0)	0.0% (n = 0)
Bacterial/lobar pneumonia	93.8% (n = 13,449)	5.8% (n = 832)	0.4% (n = 59)	0.0% (n = 0)	0.0% (n = 0)
Cardiomediastinal abnormality	79.7% (n = 11,429)	16.3% (n = 2,341)	3.3% (n = 470)	0.7% (n = 100)	0.0% (n = 0)
Cardiomegaly	87.7% (n = 12,583)	11.1% (n = 1,587)	1.2% (n = 170)	0.0% (n = 0)	0.0% (n = 0)
Consolidation	93.1% (n = 13,346)	5.9% (n = 852)	1.0% (n = 142)	0.0% (n = 0)	0.0% (n = 0)
Edema	60.9% (n = 8,732)	26.6% (n = 3,809)	10.6% (n = 1,516)	1.6% (n = 228)	0.4% (n = 55)
Lesion	98.8% (n = 14,168)	1.2% (n = 172)	0.0% (n = 0)	0.0% (n = 0)	0.0% (n = 0)
Pleural effusion	96.1% (n = 13,786)	3.6% (n = 510)	0.3% (n = 44)	0.0% (n = 0)	0.0% (n = 0)
Pleural other	99.8% (n = 14,310)	0.2% (n = 30)	0.0% (n = 0)	0.0% (n = 0)	0.0% (n = 0)
Pneumothorax	99.8% (n = 14,309)	0.0% (n = 0)	0.0% (n = 0)	0.2% (n = 31)	0.0% (n = 0)
Rib fracture	96.3% (n = 13,810)	2.9% (n = 415)	0.6% (n = 82)	0.2% (n = 33)	0.0% (n = 0)
Shoulder fracture	97.3% (n = 13,954)	2.7% (n = 386)	0.0% (n = 0)	0.0% (n = 0)	0.0% (n = 0)
Support device hardware	75.0% (n = 10,762)	16.8% (n = 2,402)	5.6% (n = 804)	2.6% (n = 372)	0.0% (n = 0)

Supplementary Table 12 | Treatment effects of signed AI error ranges and overall treatment effect across ranges. The treatment effects, 95% confidence intervals and p-values of different signed AI error ranges. The Wald test was used to test regression coefficients that estimate treatment effects against the null hypothesis of joint equality among treatment effects of different subgroups. Details of the statistical models are available in Methods. The Benjamini-Hochberg procedure was used to correct for multiple hypothesis testing over 15 individual pathologies. Non-applicable results are marked by N/A.

	AI signed error in [-100, -80]	AI signed error in (-80, -60]	AI signed error in (-60, -40]	AI signed error in (-40, -20]	AI signed error in (-20, 0]	AI signed error in [0, 20]	AI signed error in (20, 40]	*...... continued in the following table ......*
All pathologies aggregated	N/A	-3.560 (95% CI: -9.472 – 2.353)	2.089 (95% CI: 0.334 – 3.844)	2.698 (95% CI: 1.497 – 3.899)	2.483 (95% CI: 1.897 – 3.070)	0.398 (95% CI: 0.235 – 0.561)	-2.933 (95% CI: -3.787 – -2.079)	*...... continued in the following table ......*
Abnormal	N/A	N/A	N/A	N/A	2.621 (95% CI: 0.176 – 5.066)	2.884 (95% CI: 1.133 – 4.634)	1.023 (95% CI: -0.275 – 2.320)	*...... continued in the following table ......*
Airspace opacity	N/A	N/A	3.414 (95% CI: 2.804 – 4.024)	3.375 (95% CI: 0.562 – 6.188)	7.615 (95% CI: 5.836 – 9.394)	1.157 (95% CI: 0.511 – 1.803)	-4.037 (95% CI: -5.419 – -2.656)	*...... continued in the following table ......*
Atelectasis	N/A	N/A	4.736 (95% CI: 0.717 – 8.756)	4.394 (95% CI: 1.843 – 6.944)	2.934 (95% CI: 1.405 – 4.463)	0.122 (95% CI: -0.322 – 0.566)	-5.027 (95% CI: -7.465 – -2.589)	*...... continued in the following table ......*
Bacterial/lobar pneumonia	N/A	N/A	7.584 (95% CI: 6.772 – 8.396)	0.854 (95% CI: -1.381 – 3.089)	2.767 (95% CI: 0.978 – 4.556)	-0.082 (95% CI: -0.383 – 0.219)	-3.792 (95% CI: -8.663 – 1.080)	*...... continued in the following table ......*
Cardiomediastinal abnormality	N/A	-5.661 (95% CI: -10.832 – -0.489)	1.283 (95% CI: 0.102 – 2.463)	1.061 (95% CI: -0.527 – 2.650)	3.148 (95% CI: 2.054 – 4.242)	1.244 (95% CI: 0.451 – 2.037)	-4.275 (95% CI: -7.721 – -0.828)	*...... continued in the following table ......*
Cardiomegaly	N/A	N/A	2.568 (95% CI: -1.938 – 7.075)	0.987 (95% CI: -2.685 – 4.659)	3.018 (95% CI: 1.384 – 4.651)	1.390 (95% CI: 0.719 – 2.061)	-4.247 (95% CI: -6.785 – -1.709)	*...... continued in the following table ......*
Consolidation	N/A	N/A	7.811 (95% CI: 3.546 – 12.077)	2.363 (95% CI: 0.495 – 4.231)	3.050 (95% CI: 0.864 – 5.237)	-0.228 (95% CI: -0.627 – 0.171)	-3.788 (95% CI: -9.409 – 1.833)	*...... continued in the following table ......*
Edema	N/A	N/A	N/A	N/A	1.578 (95% CI: 0.362 – 2.795)	0.585 (95% CI: 0.043 – 1.126)	-5.360 (95% CI: -6.742 – -3.979)	*...... continued in the following table ......*
Lesion	N/A	N/A	N/A	5.599 (95% CI: 3.735 – 7.463)	1.535 (95% CI: 0.840 – 2.231)	0.215 (95% CI: -0.101 – 0.531)	-5.035 (95% CI: -7.866 – -2.203)	*...... continued in the following table ......*
Pleural effusion	N/A	N/A	N/A	2.906 (95% CI: 2.413 – 3.398)	1.775 (95% CI: 0.399 – 3.151)	0.433 (95% CI: 0.034 – 0.832)	-4.586 (95% CI: -8.118 – -1.053)	*...... continued in the following table ......*
Pleural other	N/A	N/A	N/A	N/A	0.030 (95% CI: -0.152 – 0.212)	0.198 (95% CI: 0.049 – 0.346)	-2.771 (95% CI: -3.100 – -2.442)	*...... continued in the following table ......*
Pneumothorax	N/A	-12.541 (95% CI: -14.466 – -10.616)	N/A	N/A	1.001 (95% CI: -0.156 – 2.158)	0.068 (95% CI: -0.056 – 0.192)	N/A	*...... continued in the following table ......*
Rib fracture	N/A	1.343 (95% CI: -0.194 – 2.880)	-1.438 (95% CI: -7.188 – 4.311)	4.581 (95% CI: 1.199 – 7.964)	-0.343 (95% CI: -1.516 – 0.831)	0.342 (95% CI: 0.020 – 0.664)	-0.017 (95% CI: -4.194 – 4.160)	*...... continued in the following table ......*
Shoulder fracture	N/A	N/A	N/A	N/A	-0.114 (95% CI: -2.240 – 2.012)	0.206 (95% CI: 0.010 – 0.403)	0.306 (95% CI: -0.262 – 0.875)	*...... continued in the following table ......*

Support device hardware	N/A	-1.427 (95% CI: -9.211 – 6.356)	-0.997 (95% CI: -4.363 – 2.370)	4.027 (95% CI: -1.088 – 9.141)	8.259 (95% CI: 5.443 – 11.074)	0.493 (95% CI: -0.034 – 1.020)	-1.190 (95% CI: -3.689 – 1.310)	*...... continued in the following table ......*

Cont'd

	...... continued	AI signed error in (40, 60]	AI signed error in (60, 80]	AI signed error in (80, 100]	P-value for testing against joint equality	Benjamini-Hochberg adjusted p-value
All pathologies aggregated	*...... continued ......*	-5.018 (95% CI: -6.516 – -3.519)	-6.968 (95% CI: -9.346 – -4.591)	-16.845 (95% CI: -24.288 – -9.403)	2.83e-30, < 0.001	N/A
Abnormal	*...... continued ......*	-1.590 (95% CI: -2.905 – -0.274)	-5.527 (95% CI: -7.593 – -3.461)	-13.663 (95% CI: -19.261 – -8.065)	5.89e-13, < 0.001	2.21e-12, < 0.001
Airspace opacity	*...... continued ......*	-10.983 (95% CI: -13.609 – -8.357)	-23.237 (95% CI: -31.003 – -15.472)	N/A	7.19e-37, < 0.001	5.39e-36, < 0.001
Atelectasis	*...... continued ......*	N/A	N/A	N/A	6.34e-8, < 0.001	1.90e-7, < 0.001
Bacterial/lobar pneumonia	*...... continued ......*	N/A	N/A	N/A	1.000	1.364
Cardiomediastinal abnormality	*...... continued ......*	-16.209 (95% CI: -16.991 – -15.427)	N/A	N/A	1.000	1.071
Cardiomegaly	*...... continued ......*	-13.220 (95% CI: -19.742 – -6.697)	N/A	N/A	1.09e-7, < 0.001	2.72e-7, < 0.001
Consolidation	*...... continued ......*	N/A	N/A	N/A	1.81e-4, < 0.001	3.39e-4, < 0.001
Edema	*...... continued ......*	-11.839 (95% CI: -14.635 – -9.042)	-17.518 (95% CI: -25.434 – -9.601)	-35.218 (95% CI: -36.072 – -34.363)	1.000	1.000
Lesion	*...... continued ......*	N/A	N/A	N/A	6.68e-14, < 0.001	3.34e-13, < 0.001
Pleural effusion	*...... continued ......*	-4.872 (95% CI: -6.382 – -3.363)	N/A	N/A	1.000	1.154
Pleural other	*...... continued ......*	N/A	N/A	N/A	1.05e-46, < 0.001	1.57e-45, < 0.001
Pneumothorax	*...... continued ......*	N/A	N/A	N/A	1.000	1.250
Rib fracture	*...... continued ......*	N/A	N/A	N/A	2.67e-5, < 0.001	5.72e-5, < 0.001
Shoulder fracture	*...... continued ......*	N/A	N/A	N/A	0.918	1.377
Support device hardware	*...... continued ......*	-9.127 (95% CI: -16.899 – -1.356)	-4.862 (95% CI: -6.033 – -3.692)	N/A	0.086	0.143

Supplementary Table 13 | Sample sizes and percentages of signed AI error ranges. The sample sizes and percentages of radiologist predictions that fall into different signed AI error ranges for Supplementary Table 12 and Fig. 5.

	AI signed error in [-100, -80]	AI signed error in (-80, -60]	AI signed error in (-60, -40]	AI signed error in (-40, -20]	AI signed error in (-20, 0]	AI signed error in [0, 20]	AI signed error in (20, 40]	AI signed error in (40, 60]	AI signed error in (60, 80]	AI signed error in (80, 100]
All pathologies aggregated	0.0% (n = 0)	0.2% (n = 483)	1.0% (n = 2,181)	3.0% (n = 6,556)	11.5% (n = 24,658)	70.4% (n = 151,472)	8.0% (n = 17,271)	4.0% (n = 8,572)	1.6% (n = 3,536)	0.2% (n = 371)
Abnormal	0.0% (n = 0)	0.0% (n = 0)	0.0% (n = 0)	0.0% (n = 0)	3.0% (n = 429)	11.0% (n = 1,571)	23.8% (n = 3,413)	38.4% (n = 5,501)	21.7% (n = 3,110)	2.2% (n = 316)
Airspace	0.0% (n = 0)	0.0% (n = 0)	1.5% (n =	7.9% (n =	10.6% (n =	43.0% (n =	28.5% (n =	7.4% (n =	1.0% (n =	0.0% (n = 0)

opacity			213)	1,136)	1,517)	6,171)	4,091)	1,067)	145)	
Atelectasis	0.0% (n = 0)	0.0% (n = 0)	4.8% (n = 685)	9.1% (n = 1,304)	11.1% (n = 1,594)	71.2% (n = 10,210)	3.8% (n = 547)	0.0% (n = 0)	0.0% (n = 0)	0.0% (n = 0)
Bacterial/lobar pneumonia	0.0% (n = 0)	0.0% (n = 0)	0.4% (n = 59)	4.0% (n = 568)	7.9% (n = 1,126)	85.9% (n = 12,323)	1.8% (n = 264)	0.0% (n = 0)	0.0% (n = 0)	0.0% (n = 0)
Cardiomediastinal abnormality	0.0% (n = 0)	0.7% (n = 100)	2.9% (n = 420)	10.8% (n = 1,552)	18.3% (n = 2, 622)	61.4% (n = 8,807)	5.5% (n = 789)	0.3% (n = 50)	0.0% (n = 0)	0.0% (n = 0)
Cardiomegaly	0.0% (n = 0)	0.0% (n = 0)	0.5% (n = 76)	2.8% (n = 396)	11.1% (n = 1,598)	76.6% (n = 10,985)	8.3% (n = 1,191)	0.7% (n = 94)	0.0% (n = 0)	0.0% (n = 0)
Consolidation	0.0% (n = 0)	0.0% (n = 0)	1.0% (n = 142)	5.0% (n = 717)	8.6% (n = 1,232)	84.5% (n = 12,114)	0.9% (n = 135)	0.0% (n = 0)	0.0% (n = 0)	0.0% (n = 0)
Edema	0.0% (n = 0)	0.0% (n = 0)	0.0% (n = 0)	0.0% (n = 0)	2.1% (n = 305)	58.8% (n = 8,427)	26.6% (n = 3,809)	10.6% (n = 1,516)	1.6% (n = 228)	0.4% (n = 55)
Lesion	0.0% (n = 0)	0.0% (n = 0)	0.0% (n = 0)	0.5% (n = 76)	11.0% (n = 1,584)	87.8% (n = 12,584)	0.7% (n = 96)	0.0% (n = 0)	0.0% (n = 0)	0.0% (n = 0)
Pleural effusion	0.0% (n = 0)	0.0% (n = 0)	0.0% (n = 0)	0.2% (n = 29)	23.1% (n = 3,309)	73.1% (n = 10,477)	3.4% (n = 481)	0.3% (n = 44)	0.0% (n = 0)	0.0% (n = 0)
Pleural other	0.0% (n = 0)	0.0% (n = 0)	0.0% (n = 0)	0.0% (n = 0)	37.9% (n = 5,434)	61.9% (n = 8,876)	0.2% (n = 30)	0.0% (n = 0)	0.0% (n = 0)	0.0% (n = 0)
Pneumothorax	0.0% (n = 0)	0.2% (n = 31)	0.0% (n = 0)	0.0% (n = 0)	1.8% (n = 254)	98.0% (n = 14,055)	0.0% (n = 0)	0.0% (n = 0)	0.0% (n = 0)	0.0% (n = 0)
Rib fracture	0.0% (n = 0)	0.2% (n = 33)	0.6% (n = 82)	0.5% (n = 71)	10.5% (n = 1,511)	85.8% (n = 12,299)	2.4% (n = 344)	0.0% (n = 0)	0.0% (n = 0)	0.0% (n = 0)
Shoulder fracture	0.0% (n = 0)	0.0% (n = 0)	0.0% (n = 0)	0.0% (n = 0)	3.1% (n = 446)	94.2% (n = 13,508)	2.7% (n = 386)	0.0% (n = 0)	0.0% (n = 0)	0.0% (n = 0)
Support device hardware	0.0% (n = 0)	2.2% (n = 319)	3.5% (n = 504)	4.9% (n = 707)	11.8% (n = 1,697)	63.2% (n = 9,065)	11.8% (n = 1,695)	2.1% (n = 300)	0.4% (n = 53)	0.0% (n = 0)

Supplementary Table 14 | Ranges and interquartile ranges of heterogeneous unassisted errors and treatment effects on unassisted error of radiologists under binary ground truth labels. The ranges of heterogeneous unassisted errors and treatment effects on unassisted error of 140 radiologists on individual pathologies as computed using the empirical Bayes method. Uncomputable results due to dataset size and statistical restrictions are marked by U (additional explanation for the causes is available in the Methods section). Non-applicable results are marked by N/A.

	Prevalence at 50 probability threshold	Range and interquartile range (IQR) of unassisted errors	Range and interquartile range (IQR) of treatment effects on unassisted error
All pathologies aggregated	N/A	6.207 – 16.040, IQR 2.467	-1.874 – 1.141, IQR 0.808
Abnormal	19.44%	14.507 – 59.228, IQR 9.080	-7.701 – 6.143, IQR 3.887
Airspace opacity	16.05%	7.605 – 45.348, IQR 5.712	-5.189 – 0.422, IQR 1.250
Atelectasis	11.11%	0.563 – 19.081, IQR 2.444	-2.697 – 1.053, IQR 0.816

Bacterial/lobar pneumonia	1.23%	0.000 – 9.429, IQR 3.079	U
Cardiomediastinal abnormality	13.27%	9.623 – 38.563, IQR 6.413	-1.397 – 3.863, IQR 0.804
Cardiomegaly	4.32%	0.000 – 37.310, IQR 8.919	U
Consolidation	3.40%	0.000 – 12.568, IQR 3.063	U
Edema	1.85%	0.000 – 25.397, IQR 6.620	U
Lesion	0.00%	0.000 – 7.885, IQR 2.792	-3.312 – 1.225, IQR 0.707
Pleural effusion	3.70%	0.000 – 7.848, IQR 1.846	U
Pleural other	0.00%	0.000 – 1.910, IQR 0.725	U
Pneumothorax	0.31%	0.000 – 1.251, IQR 0.665	U
Rib fracture	0.93%	0.000 – 3.637, IQR 2.837	U
Shoulder fracture	0.00%	0.000 – 1.617, IQR 0.649	U
Support device hardware	17.59%	1.163 – 41.607, IQR 8.110	-4.031 – 5.123, IQR 2.550

Supplementary Table 15 | Heterogeneous treatment effects of radiologists in binary subgroups under binary ground truth labels. The treatment effects, 95% confidence intervals and p-values of 136 radiologists that have survey data on individual pathologies in binary subgroups split based on treatment effect. A two-sided, unpaired t-test between the two subgroups of treatment effects was conducted. The Benjamini-Hochberg procedure was used to correct for multiple hypothesis testing over 15 individual pathologies. The difference between subgroups shows the extent of heterogeneity an ideal predictor of treatment effect would have been able to discern. Uncomputable results due to dataset size and statistical restrictions are marked by U (additional explanation for the causes is available in the Methods section). Non-applicable results are marked by N/A.

	Lower subgroup	Higher subgroup	P-value for testing against subgroup equality	Benjamini-Hochberg adjusted p-value
All pathologies aggregated	-0.615 (95% CI: -0.697 – -0.533)	0.266 (95% CI: 0.200 – 0.331)	1.23e-34, < 0.001	N/A
Abnormal	-3.555 (95% CI: -4.000 – -3.109)	1.293 (95% CI: 0.872 – 1.714)	2.31e-32, < 0.001	1.73e-31, < 0.001
Airspace opacity	-3.017 (95% CI: -3.169 – -2.865)	-1.407 (95% CI: -1.567 – -1.246)	2.43e-29, < 0.001	1.21e-28, < 0.001
Atelectasis	-0.823 (95% CI: -0.924 – -0.722)	0.133 (95% CI: 0.043 – 0.223)	2.15e-28, < 0.001	8.06e-28, < 0.001
Bacterial/lobar pneumonia	U	U	U	U
Cardiomediastinal	0.908 (95% CI: 0.764 –	2.027 (95% CI: 1.935 –	1.26e-25, < 0.001	3.78e-25, < 0.001

abnormality	1.052)	2.120)		
Cardiomegaly	U	U	U	U
Consolidation	U	U	U	U
Edema	U	U	U	U
Lesion	-0.363 (95% CI: -0.474 – -0.252)	0.436 (95% CI: 0.372 – 0.501)	4.00e-24, < 0.001	1.10e-23, < 0.001
Pleural effusion	U	U	U	U
Pleural other	U	U	U	U
Pneumothorax	U	U	U	U
Rib fracture	U	U	U	U
Shoulder fracture	U	U	U	U
Support device hardware	-0.263 (95% CI: -0.514 – -0.012)	2.668 (95% CI: 2.398 – 2.938)	1.42e-32, < 0.001	2.13e-31, < 0.001

Supplementary Table 16 | Treatment effects of subgroups split based on combined characteristics under binary ground truth labels. The treatment effects, 95% confidence intervals and p-values of radiologists in binary subgroups split based on combined characteristics of years of experience, subspecialty in thoracic radiology and experience with AI tools. The Wald test was used to test regression coefficients that estimate treatment effects against the null hypothesis of joint equality among treatment effects of different subgroups. Details of the statistical models are available in Methods. The Benjamini-Hochberg procedure was used to correct for multiple hypothesis testing over 15 individual pathologies. Non-applicable results are marked by N/A.

	Lower subgroup	Higher subgroup	P-value for testing against subgroup equality	Benjamini-Hochberg adjusted p-value
All pathologies aggregated	-0.170 (95% CI: -0.498 – 0.159)	-0.050 (95% CI: -0.484 – 0.384)	0.638	N/A
Abnormal	-1.216 (95% CI: -2.791 – 0.360)	-0.261 (95% CI: -1.879 – 1.356)	0.360	1.079
Airspace opacity	-1.828 (95% CI: -2.951 – -0.706)	-1.861 (95% CI: -3.927 – 0.204)	0.975	1.045
Atelectasis	-0.083 (95% CI: -1.360 – 1.193)	-0.341 (95% CI: -1.346 – 0.665)	0.748	1.020
Bacterial/lobar pneumonia	0.035 (95% CI: -0.489 – 0.559)	0.269 (95% CI: -0.554 – 1.093)	0.593	0.989
Cardiomediastinal abnormality	0.825 (95% CI: -0.231 – 1.882)	1.549 (95% CI: -0.024 – 3.123)	0.447	0.957

Cardiomegaly	0.989 (95% CI: -0.965 – 2.943)	0.840 (95% CI: -0.115 – 1.796)	0.888	1.024
Consolidation	-0.003 (95% CI: -0.791 – 0.784)	-0.320 (95% CI: -0.921 – 0.281)	0.467	0.877
Edema	-3.312 (95% CI: -4.477 – -2.146)	-2.965 (95% CI: -4.104 – -1.825)	0.605	0.907
Lesion	0.396 (95% CI: -0.008 – 0.799)	0.090 (95% CI: -0.509 – 0.689)	0.394	0.986
Pleural effusion	0.815 (95% CI: 0.184 – 1.445)	-0.012 (95% CI: -0.842 – 0.818)	0.088	1.321
Pleural other	0.267 (95% CI: -0.024 – 0.559)	0.092 (95% CI: -0.054 – 0.237)	0.282	1.059
Pneumothorax	0.218 (95% CI: -0.032 – 0.469)	-0.156 (95% CI: -0.573 – 0.260)	0.225	1.126
Rib fracture	-0.256 (95% CI: -0.955 – 0.442)	0.400 (95% CI: -0.067 – 0.867)	0.096	0.721
Shoulder fracture	0.181 (95% CI: -0.016 – 0.379)	0.147 (95% CI: -0.116 – 0.410)	0.792	0.990
Support device hardware	0.983 (95% CI: -0.431 – 2.396)	0.994 (95% CI: -0.040 – 2.028)	0.989	0.989

Supplementary Table 17 | Treatment effects of subgroups split based on years of experience under binary ground truth labels. The treatment effects, 95% confidence intervals and p-values of radiologists in binary subgroups split based on years of experience. The Wald test was used to test regression coefficients that estimate treatment effects against the null hypothesis of joint equality among treatment effects of different subgroups. Details of the statistical models are available in Methods. The Benjamini-Hochberg procedure was used to correct for multiple hypothesis testing over 15 individual pathologies. Non-applicable results are marked by N/A.

	Subgroup of less than or equal to 6 years of experience	Subgroup of more than 6 years of experience	P-value for testing against subgroup equality	Benjamini-Hochberg adjusted p-value
All pathologies aggregated	0.023 (95% CI: -0.442 – 0.489)	-0.229 (95% CI: -0.537 – 0.080)	0.340	N/A
Abnormal	0.147 (95% CI: -1.712 – 2.005)	-1.487 (95% CI: -2.948 – -0.025)	0.137	0.685
Airspace opacity	-1.669 (95% CI: -3.394 – 0.057)	-1.950 (95% CI: -3.132 – -0.767)	0.761	0.878
Atelectasis	0.096 (95% CI: -1.248 – 1.439)	-0.511 (95% CI: -1.434 – 0.411)	0.429	0.715
Bacterial/lobar pneumonia	0.008 (95% CI: -0.632 – 0.649)	0.131 (95% CI: -0.450 – 0.712)	0.747	0.933

Cardiomediastinal abnormality	1.589 (95% CI: 0.098 – 3.080)	0.804 (95% CI: -0.230 – 1.839)	0.366	0.686
Cardiomegaly	0.313 (95% CI: -1.399 – 2.026)	1.278 (95% CI: 0.266 – 2.290)	0.309	0.774
Consolidation	-0.008 (95% CI: -0.863 – 0.846)	-0.250 (95% CI: -0.882 – 0.382)	0.612	0.835
Edema	-3.623 (95% CI: -4.953 – -2.293)	-2.846 (95% CI: -3.921 – -1.771)	0.292	0.876
Lesion	0.042 (95% CI: -0.480 – 0.565)	0.457 (95% CI: 0.027 – 0.887)	0.215	0.807
Pleural effusion	0.416 (95% CI: -0.507 – 1.338)	0.401 (95% CI: -0.204 – 1.006)	0.977	0.977
Pleural other	0.048 (95% CI: -0.101 – 0.197)	0.155 (95% CI: -0.025 – 0.335)	0.318	0.680
Pneumothorax	0.083 (95% CI: -0.121 – 0.288)	-0.016 (95% CI: -0.254 – 0.221)	0.517	0.776
Rib fracture	0.598 (95% CI: 0.009 – 1.187)	0.028 (95% CI: -0.487 – 0.543)	0.118	0.887
Shoulder fracture	0.190 (95% CI: -0.107 – 0.487)	0.138 (95% CI: -0.155 – 0.430)	0.805	0.863
Support device hardware	2.120 (95% CI: 0.589 – 3.652)	0.240 (95% CI: -0.863 – 1.344)	0.054	0.807

Supplementary Table 18 | Treatment effects of subgroups split based on subspecialty in thoracic radiology under binary ground truth labels. The treatment effects, 95% confidence intervals and p-values of radiologists in binary subgroups split based on subspecialty in thoracic radiology. The Wald test was used to test regression coefficients that estimate treatment effects against the null hypothesis of joint equality among treatment effects of different subgroups. Details of the statistical models are available in Methods. The Benjamini-Hochberg procedure was used to correct for multiple hypothesis testing over 15 individual pathologies. Non-applicable results are marked by N/A.

	Subgroup of radiologists that do not specialize in thoracic radiology	Subgroup of radiologists that specialize in thoracic radiology	P-value for testing against subgroup equality	Benjamini-Hochberg adjusted p-value
All pathologies aggregated	-0.131 (95% CI: -0.457 – 0.194)	-0.118 (95% CI: -0.559 – 0.322)	0.959	N/A
Abnormal	-0.294 (95% CI: -1.643 – 1.055)	-2.337 (95% CI: -4.713 – 0.040)	0.122	1.825
Airspace opacity	-1.789 (95% CI: -3.057 – -0.521)	-1.973 (95% CI: -3.552 – -0.393)	0.838	1.396
Atelectasis	-0.383 (95% CI: -1.304 – 0.539)	0.046 (95% CI: -1.313 – 1.406)	0.568	1.419

Bacterial/lobar pneumonia	0.099 (95% CI: -0.482 – 0.680)	0.035 (95% CI: -0.861 – 0.930)	0.906	1.133
Cardiomediastinal abnormality	1.158 (95% CI: 0.149 – 2.168)	1.005 (95% CI: -0.609 – 2.618)	0.865	1.297
Cardiomegaly	0.769 (95% CI: -0.308 – 1.845)	1.239 (95% CI: -0.359 – 2.837)	0.588	1.261
Consolidation	-0.170 (95% CI: -0.836 – 0.497)	-0.110 (95% CI: -1.127 – 0.907)	0.922	1.064
Edema	-3.150 (95% CI: -4.218 – -2.082)	-3.172 (95% CI: -4.562 – -1.782)	0.977	0.977
Lesion	0.250 (95% CI: -0.150 – 0.650)	0.407 (95% CI: -0.260 – 1.074)	0.690	1.293
Pleural effusion	0.388 (95% CI: -0.200 – 0.977)	0.458 (95% CI: -0.577 – 1.493)	0.899	1.225
Pleural other	0.111 (95% CI: -0.047 – 0.269)	0.116 (95% CI: -0.091 – 0.323)	0.967	1.036
Pneumothorax	-0.049 (95% CI: -0.280 – 0.183)	0.224 (95% CI: -0.027 – 0.474)	0.160	1.199
Rib fracture	0.097 (95% CI: -0.390 – 0.583)	0.697 (95% CI: -0.118 – 1.513)	0.215	0.805
Shoulder fracture	0.241 (95% CI: -0.061 – 0.543)	-0.071 (95% CI: -0.366 – 0.224)	0.210	1.052
Support device hardware	0.749 (95% CI: -0.330 – 1.829)	1.662 (95% CI: 0.062 – 3.262)	0.350	1.050

Supplementary Table 19 | Treatment effects of subgroups split based on experience with AI tools under binary ground truth labels. The treatment effects, 95% confidence intervals and p-values of radiologists in binary subgroups split based on experience with AI tools. The Wald test was used to test regression coefficients that estimate treatment effects against the null hypothesis of joint equality among treatment effects of different subgroups. Details of the statistical models are available in Methods. The Benjamini-Hochberg procedure was used to correct for multiple hypothesis testing over 15 individual pathologies. Non-applicable results are marked by N/A.

	Subgroup of radiologists that do not have experience with AI tools	Subgroup of radiologists that have experience with AI tools	P-value for testing against subgroup equality	Benjamini-Hochberg adjusted p-value
All pathologies aggregated	-0.257 (95% CI: -0.841 – 0.327)	-0.074 (95% CI: -0.362 – 0.214)	0.563	N/A
Abnormal	-1.790 (95% CI: -4.088 – 0.508)	-0.433 (95% CI: -1.717 – 0.851)	0.266	0.999
Airspace opacity	-2.858 (95% CI: -4.672 – -1.044)	-1.409 (95% CI: -2.558 – -0.259)	0.116	0.580

Atelectasis	-0.490 (95% CI: -2.092 – 1.112)	-0.176 (95% CI: -1.045 – 0.692)	0.717	0.768
Bacterial/lobar pneumonia	0.342 (95% CI: -0.483 – 1.167)	-0.027 (95% CI: -0.562 – 0.507)	0.418	0.783
Cardiomediastinal abnormality	2.312 (95% CI: 0.531 – 4.093)	0.615 (95% CI: -0.360 – 1.591)	0.089	0.669
Cardiomegaly	0.932 (95% CI: -1.200 – 3.064)	0.877 (95% CI: -0.126 – 1.880)	0.962	0.962
Consolidation	-0.574 (95% CI: -1.759 – 0.612)	0.023 (95% CI: -0.551 – 0.597)	0.353	1.058
Edema	-4.601 (95% CI: -6.132 – -3.070)	-2.549 (95% CI: -3.497 – -1.600)	0.007	0.098
Lesion	0.054 (95% CI: -0.625 – 0.734)	0.391 (95% CI: -0.010 – 0.792)	0.404	0.865
Pleural effusion	0.225 (95% CI: -0.930 – 1.379)	0.483 (95% CI: -0.078 – 1.045)	0.673	0.842
Pleural other	0.186 (95% CI: -0.033 – 0.405)	0.081 (95% CI: -0.078 – 0.240)	0.430	0.717
Pneumothorax	-0.052 (95% CI: -0.333 – 0.229)	0.055 (95% CI: -0.153 – 0.263)	0.548	0.747
Rib fracture	0.523 (95% CI: -0.541 – 1.588)	0.143 (95% CI: -0.320 – 0.606)	0.540	0.811
Shoulder fracture	0.209 (95% CI: -0.144 – 0.563)	0.137 (95% CI: -0.097 – 0.371)	0.714	0.824
Support device hardware	1.722 (95% CI: -0.512 – 3.955)	0.684 (95% CI: -0.091 – 1.458)	0.372	0.930

Supplementary Table 20 | Regression results for treatment effect vs. unassisted error under binary ground truth labels. The regression coefficients, adjustment for attenuation bias and p-values of the analysis on the relationship between treatment effect and unassisted error. The Wald test was used to test regression coefficients against the null hypothesis of zero to determine in a continuous analysis if the independent variable is a predictor of the dependent variable. Details of the statistical models are available in Methods. The Benjamini-Hochberg procedure was used to correct for multiple hypothesis testing over 15 individual pathologies. Underscore indicates a difference in the status of statistical significance from that of the same pathology under continuous ground truth probabilities. Non-applicable results are marked by N/A.

	Unassisted error coefficient before adjustment for attenuation bias	Intercept coefficient before adjustment for attenuation bias	Unassisted error coefficient after adjustment for attenuation bias	P-value for testing unassisted error coefficient against 0	Benjamini-Hochberg adjusted p-value
All pathologies	0.034 (95% CI:	-0.556 (95% CI:	0.040 (95% CI:	0.601	N/A

aggregated	-0.094 – 0.163)	-1.953 – 0.841)	-0.111 – 0.191)		
Abnormal	0.151 (95% CI: 0.025 – 0.277)	-7.352 (95% CI: -12.919 – -1.785)	0.191 (95% CI: 0.032 – 0.351)	0.019	0.141
Airspace opacity	0.092 (95% CI: -0.034 – 0.218)	-4.049 (95% CI: -7.055 – -1.043)	0.150 (95% CI: -0.055 – 0.355)	0.152	0.379
Atelectasis	0.203 (95% CI: 0.011 – 0.394)	-2.976 (95% CI: -5.471 – -0.481)	0.549 (95% CI: 0.029 – 1.069)	0.038	0.192
Bacterial/lobar pneumonia	0.026 (95% CI: -0.121 – 0.173)	-0.105 (95% CI: -0.837 – 0.627)	0.045 (95% CI: -0.211 – 0.302)	0.730	0.912
Cardiomediastinal abnormality	-0.045 (95% CI: -0.242 – 0.152)	2.302 (95% CI: -2.153 – 6.757)	-0.070 (95% CI: -0.380 – 0.239)	0.656	0.894
Cardiomegaly	0.146 (95% CI: -0.028 – 0.320)	-1.524 (95% CI: -4.189 – 1.142)	0.194 (95% CI: -0.037 – 0.426)	0.100	0.374
Consolidation	0.073 (95% CI: -0.063 – 0.209)	-0.768 (95% CI: -1.816 – 0.279)	0.142 (95% CI: -0.121 – 0.404)	0.290	0.622
Edema	-0.008 (95% CI: -0.122 – 0.106)	-3.518 (95% CI: -4.807 – -2.228)	-0.010 (95% CI: -0.155 – 0.135)	0.890	0.890
Lesion	0.166 (95% CI: 0.072 – 0.261)	-0.285 (95% CI: -0.711 – 0.142)	0.224 (95% CI: 0.096 – 0.352)	5.82e-4, < 0.001	0.009
Pleural effusion	0.060 (95% CI: -0.137 – 0.258)	0.040 (95% CI: -1.056 – 1.136)	0.159 (95% CI: -0.363 – 0.681)	0.550	0.825
Pleural other	-0.015 (95% CI: -0.219 – 0.189)	0.158 (95% CI: 0.002 – 0.314)	-0.029 (95% CI: -0.432 – 0.373)	0.886	0.950
Pneumothorax	0.087 (95% CI: -0.098 – 0.272)	-0.085 (95% CI: -0.392 – 0.222)	0.293 (95% CI: -0.331 – 0.918)	0.357	0.595
Rib fracture	0.027 (95% CI: -0.127 – 0.180)	0.332 (95% CI: -0.246 – 0.909)	0.102 (95% CI: -0.486 – 0.690)	0.734	0.847
Shoulder fracture	0.097 (95% CI: -0.091 – 0.284)	0.104 (95% CI: -0.147 – 0.354)	0.523 (95% CI: -0.490 – 1.537)	0.311	0.584
Support device hardware	0.124 (95% CI: -0.039 – 0.287)	-0.465 (95% CI: -2.574 – 1.644)	0.157 (95% CI: -0.050 – 0.364)	0.137	0.410

Supplementary Table 21 | Regression results for treatment effect vs. unassisted error without split sampling under binary ground truth labels. The regression coefficients, adjustment for attenuation bias and p-values of the hypothetical analysis on the relationship between treatment effect and unassisted error without split sampling. The Wald test was used to test regression coefficients against the null hypothesis of zero to determine in a continuous analysis if the independent variable is a predictor of the dependent variable. Details of the statistical models are available in Methods. The Benjamini-Hochberg procedure was used to correct for multiple hypothesis testing over 15 individual pathologies. Underscore indicates a difference in the status of statistical significance from that of the same pathology under continuous ground truth probabilities.

	Unassisted error coefficient before adjustment for attenuation bias	Intercept coefficient before adjustment for attenuation bias	Unassisted error coefficient after adjustment for attenuation bias	P-value for testing unassisted error coefficient against 0	Benjamini-Hochberg adjusted p-value
All pathologies aggregated	0.256 (95% CI: 0.131 – 0.380)	-2.948 (95% CI: -4.270 – -1.626)	0.298 (95% CI: 0.153 – 0.443)	5.58e-5, < 0.001	N/A
Abnormal	0.323 (95% CI: 0.213 – 0.433)	-14.517 (95% CI: -19.351 – -9.682)	0.404 (95% CI: 0.266 – 0.542)	8.63e-9, < 0.001	1.44e-8, < 0.001
Airspace opacity	0.414 (95% CI: 0.288 – 0.541)	-10.834 (95% CI: -13.722 – -7.945)	0.664 (95% CI: 0.461 – 0.867)	1.50e-10, < 0.001	3.75e-10, < 0.001
Atelectasis	0.772 (95% CI: 0.624 – 0.920)	-10.274 (95% CI: -12.107 – -8.441)	2.105 (95% CI: 1.701 – 2.509)	1.77e-24, < 0.001	2.66e-23, < 0.001
Bacterial/lobar pneumonia	0.397 (95% CI: 0.270 – 0.524)	-1.625 (95% CI: -2.196 – -1.054)	0.690 (95% CI: 0.469 – 0.911)	9.80e-10, < 0.001	2.10e-9, < 0.001
Cardiomediastinal abnormality	0.278 (95% CI: 0.129 – 0.428)	-5.171 (95% CI: -8.501 – -1.841)	0.428 (95% CI: 0.198 – 0.658)	2.66e-4, < 0.001	3.07e-4, < 0.001
Cardiomegaly	0.356 (95% CI: 0.197 – 0.514)	-4.947 (95% CI: -7.301 – -2.594)	0.463 (95% CI: 0.257 – 0.670)	1.12e-5, < 0.001	1.53e-5, < 0.001
Consolidation	0.505 (95% CI: 0.380 – 0.629)	-3.839 (95% CI: -4.715 – -2.962)	0.967 (95% CI: 0.728 – 1.205)	1.96e-15, < 0.001	9.80e-15, < 0.001
Edema	0.155 (95% CI: 0.047 – 0.262)	-5.090 (95% CI: -6.341 – -3.839)	0.197 (95% CI: 0.060 – 0.334)	0.005	0.005
Lesion	0.423 (95% CI: 0.252 – 0.593)	-1.061 (95% CI: -1.587 – -0.535)	0.575 (95% CI: 0.343 – 0.807)	1.20e-6, < 0.001	1.79e-6, < 0.001
Pleural effusion	0.569 (95% CI: 0.378 – 0.759)	-2.348 (95% CI: -3.242 – -1.453)	1.459 (95% CI: 0.970 – 1.947)	4.78e-9, < 0.001	8.96e-9, < 0.001
Pleural other	0.383 (95% CI: 0.006 – 0.760)	-0.125 (95% CI: -0.319 – 0.069)	0.750 (95% CI: 0.013 – 1.487)	0.046	0.046
Pneumothorax	0.758 (95% CI: 0.549 – 0.967)	-0.535 (95% CI: -0.800 – -0.269)	2.594 (95% CI: 1.878 – 3.310)	1.21e-12, < 0.001	3.63e-12, < 0.001
Rib fracture	0.617 (95% CI: 0.450 – 0.784)	-1.263 (95% CI: -1.678 – -0.847)	2.162 (95% CI: 1.576 – 2.748)	4.69e-13, < 0.001	1.76e-12, < 0.001
Shoulder fracture	0.843 (95% CI: 0.664 – 1.023)	-0.387 (95% CI: -0.566 – -0.209)	4.445 (95% CI: 3.498 – 5.391)	3.35e-20, < 0.001	2.52e-19, < 0.001
Support device hardware	0.322 (95% CI: 0.154 – 0.491)	-3.153 (95% CI: -5.218 – -1.087)	0.404 (95% CI: 0.193 – 0.616)	1.80e-4, < 0.001	2.25e-4, < 0.001

Supplementary Table 22 | Regression results for assisted error vs. unassisted error under binary ground truth labels. The regression coefficients, adjustment for attenuation bias and p-values of the analysis on the relationship between assisted error and unassisted error. The Wald test was used to test regression coefficients against the null hypothesis of zero to determine in a continuous analysis if the independent variable is a predictor of the dependent variable. Details of the statistical models are

available in Methods. The Benjamini-Hochberg procedure was used to correct for multiple hypothesis testing over 15 individual pathologies. Underscore indicates a difference in the status of statistical significance from that of the same pathology under continuous ground truth probabilities. Non-applicable results are marked by N/A.

	Unassisted error coefficient before adjustment for attenuation bias	Intercept coefficient before adjustment for attenuation bias	Unassisted error coefficient after adjustment for attenuation bias	P-value for testing unassisted error coefficient against 0	Benjamini-Hochberg adjusted p-value
All pathologies aggregated	0.695 (95% CI: 0.572 – 0.819)	3.476 (95% CI: 2.166 – 4.787)	0.811 (95% CI: 0.667 – 0.956)	3.34e-28, < 0.001	N/A
Abnormal	0.633 (95% CI: 0.526 – 0.740)	16.364 (95% CI: 11.634 – 21.094)	0.793 (95% CI: 0.659 – 0.927)	5.93e-31, < 0.001	4.45e-30, < 0.001
Airspace opacity	0.536 (95% CI: 0.406 – 0.666)	11.885 (95% CI: 8.967 – 14.802)	0.860 (95% CI: 0.651 – 1.068)	5.90e-16, < 0.001	1.77e-15, < 0.001
Atelectasis	0.107 (95% CI: -0.029 – 0.243)	11.824 (95% CI: 10.103 – 13.545)	0.293 (95% CI: -0.080 – 0.665)	0.124	0.143
Bacterial/lobar pneumonia	0.554 (95% CI: 0.427 – 0.681)	1.827 (95% CI: 1.258 – 2.396)	0.971 (95% CI: 0.748 – 1.194)	1.29e-17, < 0.001	4.83e-17, < 0.001
Cardiomediastinal abnormality	0.671 (95% CI: 0.525 – 0.817)	6.334 (95% CI: 3.073 – 9.594)	1.032 (95% CI: 0.807 – 1.256)	1.99e-19, < 0.001	9.95e-19, < 0.001
Cardiomegaly	0.610 (95% CI: 0.453 – 0.767)	5.510 (95% CI: 3.188 – 7.831)	0.796 (95% CI: 0.591 – 1.001)	2.40e-14, < 0.001	5.99e-14, < 0.001
Consolidation	0.438 (95% CI: 0.313 – 0.562)	4.248 (95% CI: 3.374 – 5.121)	0.848 (95% CI: 0.607 – 1.089)	5.42e-12, < 0.001	1.02e-11, < 0.001
Edema	0.814 (95% CI: 0.704 – 0.923)	5.394 (95% CI: 4.129 – 6.660)	1.037 (95% CI: 0.897 – 1.176)	6.01e-48, < 0.001	9.01e-47, < 0.001
Lesion	0.559 (95% CI: 0.376 – 0.742)	1.117 (95% CI: 0.560 – 1.673)	0.761 (95% CI: 0.512 – 1.009)	2.03e-9, < 0.001	3.38e-9, < 0.001
Pleural effusion	0.324 (95% CI: 0.142 – 0.506)	2.850 (95% CI: 1.979 – 3.722)	0.820 (95% CI: 0.359 – 1.280)	4.84e-4, < 0.001	0.001
Pleural other	0.576 (95% CI: 0.224 – 0.929)	0.154 (95% CI: -0.022 – 0.329)	1.117 (95% CI: 0.433 – 1.800)	0.001	0.002
Pneumothorax	0.140 (95% CI: -0.053 – 0.333)	0.603 (95% CI: 0.332 – 0.874)	0.477 (95% CI: -0.181 – 1.136)	0.155	0.166
Rib fracture	0.252 (95% CI: 0.102 – 0.402)	1.618 (95% CI: 1.180 – 2.055)	0.917 (95% CI: 0.370 – 1.463)	0.001	0.001
Shoulder fracture	0.075 (95% CI: -0.077 – 0.228)	0.441 (95% CI: 0.257 – 0.625)	0.396 (95% CI: -0.405 – 1.196)	0.333	0.333
Support device hardware	0.655 (95% CI: 0.482 – 0.827)	3.467 (95% CI: 1.365 – 5.569)	0.822 (95% CI: 0.605 – 1.038)	9.61e-14, < 0.001	2.06e-13, < 0.001

Supplementary Table 23 | Treatment effects of absolute AI error ranges and overall treatment effect across ranges under binary ground truth labels. The treatment effects, 95% confidence intervals and p-values of different absolute AI error ranges and the statistics for the overall treatment effect across ranges. The Wald test was used to test regression coefficients that estimate treatment effects against the null hypothesis of joint equality among treatment effects of different subgroups. Details of the statistical models are available in Methods. The Benjamini-Hochberg procedure was used to correct for multiple hypothesis testing over 15 individual pathologies. Underscore indicates a difference in the status of statistical significance and/or treatment effect trend from that of the same pathology under continuous ground truth probabilities. Non-applicable results are marked by N/A.

	AI absolute error in [0, 20]	AI absolute error in (20, 40]	AI absolute error in (40, 60]	AI absolute error in (60, 80]	AI absolute error in (80, 100]	P-value for testing against joint equality	Benjamini-Hochberg adjusted p-value	Across AI absolute error ranges
All pathologies aggregated	0.532 (95% CI: 0.345 – 0.719)	-1.355 (95% CI: -2.341 – -0.368)	-2.901 (95% CI: -4.586 – -1.215)	-3.632 (95% CI: -5.373 – -1.891)	-6.469 (95% CI: -8.690 – -4.247)	1.49e-12, < 0.001	N/A	-0.163 (95% CI: -0.432 – 0.107)
Abnormal	4.520 (95% CI: 2.516 – 6.523)	5.757 (95% CI: 2.407 – 9.107)	2.247 (95% CI: 0.776 – 3.717)	-3.225 (95% CI: -5.098 – -1.352)	-7.560 (95% CI: -9.767 – -5.354)	6.85e-19, < 0.001	3.43e-18, < 0.001	-0.829 (95% CI: -2.010 – 0.353)
Airspace opacity	1.417 (95% CI: 0.727 – 2.107)	-2.754 (95% CI: -4.379 – -1.128)	-5.142 (95% CI: -8.292 – -1.993)	-5.324 (95% CI: -10.622 – -0.027)	-12.337 (95% CI: -28.810 – 4.136)	8.21e-9, < 0.001	2.46e-8, < 0.001	-1.851 (95% CI: -2.915 – -0.787)
Atelectasis	0.168 (95% CI: -0.278 – 0.613)	-6.318 (95% CI: -8.424 – -4.213)	4.431 (95% CI: -2.860 – 11.722)	5.482 (95% CI: 1.212 – 9.751)	-0.896 (95% CI: -7.192 – 5.400)	4.24e-8, < 0.001	1.06e-7, < 0.001	-0.375 (95% CI: -1.175 – 0.425)
Bacterial/lobar pneumonia	0.026 (95% CI: -0.346 – 0.398)	-5.942 (95% CI: -10.609 – -1.274)	-3.102 (95% CI: -22.223 – 16.018)	0.333 (95% CI: -1.199 – 1.865)	6.029 (95% CI: 4.964 – 7.094)	0.110	0.150	0.000 (95% CI: -0.477 – 0.476)
Cardiomediastinal abnormality	1.553 (95% CI: 0.565 – 2.540)	1.442 (95% CI: -0.923 – 3.807)	-2.633 (95% CI: -7.288 – 2.022)	-2.602 (95% CI: -6.226 – 1.023)	-4.112 (95% CI: -7.313 – -0.911)	0.002	0.004	0.964 (95% CI: 0.081 – 1.847)
Cardiomegaly	1.727 (95% CI: 0.925 – 2.529)	0.465 (95% CI: -2.056 – 2.987)	-3.409 (95% CI: -7.919 – 1.102)	-9.473 (95% CI: -15.900 – -3.047)	3.279 (95% CI: 1.365 – 5.193)	0.002	0.004	0.870 (95% CI: -0.066 – 1.807)
Consolidation	-0.227 (95% CI: -0.678 – 0.224)	-4.731 (95% CI: -8.701 – -0.762)	5.060 (95% CI: -12.502 – 22.623)	8.549 (95% CI: 2.133 – 14.965)	-3.571 (95% CI: -10.427 – 3.285)	0.024	0.040	-0.218 (95% CI: -0.765 – 0.329)
Edema	0.550 (95% CI: 0.022 – 1.077)	-5.366 (95% CI: -6.799 – -3.932)	-11.247 (95% CI: -14.185 – -8.309)	-16.804 (95% CI: -22.584 – -11.024)	-35.218 (95% CI: -36.073 – -34.364)	1.000	1.000	-3.300 (95% CI: -4.233 – -2.366)
Lesion	0.330 (95% CI: -0.007 – 0.666)	-5.354 (95% CI: -7.004 – -3.704)	N/A	N/A	N/A	2.67e-10, < 0.001	1.00e-9, < 0.001	0.252 (95% CI: -0.082 – 0.586)
Pleural effusion	0.409 (95% CI: 0.070 – 0.748)	4.990 (95% CI: -0.447 – 10.428)	-2.917 (95% CI: -10.522 – 4.688)	-23.019 (95% CI: -24.172 – -21.866)	N/A	1.000	1.071	0.447 (95% CI: -0.102 – 0.996)
Pleural other	0.125 (95% CI: -0.002 – 0.253)	-2.771 (95% CI: -3.099 – -2.442)	N/A	N/A	N/A	9.86e-43, < 0.001	1.48e-41, < 0.001	0.119 (95% CI: -0.008 – 0.247)
Pneumothorax	0.068 (95% CI: -0.060 – 0.196)	N/A	N/A	N/A	-14.365 (95% CI: -16.617 – -12.112)	1.14e-39, < 0.001	8.54e-39, < 0.001	0.013 (95% CI: -0.164 – 0.191)
Rib fracture	0.195 (95% CI: -0.135 – 0.525)	1.259 (95% CI: -2.886 – 5.405)	N/A	N/A	-3.273 (95% CI: -15.357 – 8.812)	0.750	0.865	0.301 (95% CI: -0.100 – 0.703)
Shoulder fracture	0.164 (95% CI: -0.036 – 0.364)	0.658 (95% CI: -0.196 – 1.511)	N/A	N/A	N/A	0.276	0.345	0.178 (95% CI: -0.015 – 0.372)
Support device hardware	1.671 (95% CI: 0.889 – 2.452)	1.325 (95% CI: -1.090 – 3.740)	-3.758 (95% CI: -9.571 – 2.055)	-3.754 (95% CI: -8.756 – 1.248)	-1.450 (95% CI: -9.145 – 6.245)	0.080	0.120	0.989 (95% CI: 0.112 – 1.865)

Supplementary Table 24 | Sample sizes and percentages of absolute AI error ranges under binary ground truth labels. The sample sizes and percentages of radiologist predictions that fall into different absolute AI error ranges for Supplementary Table 23.

	AI absolute error in [0, 20]	AI absolute error in (20, 40]	AI absolute error in (40, 60]	AI absolute error in (60, 80]	AI absolute error in (80, 100]
All pathologies aggregated	78.7% (n = 169,219)	9.8% (n = 21,020)	5.4% (n = 11,643)	3.9% (n = 8,464)	2.2% (n = 4,754)
Abnormal	16.6% (n = 2,376)	7.2% (n = 1,036)	23.6% (n = 3,380)	31.8% (n = 4,566)	20.8 (n = 2,982)
Airspace opacity	39.4% (n = 5,645)	34.4% (n = 4,936)	19.1% (n = 2,734)	5.8% (n = 837)	1.3% (n = 188)
Atelectasis	76.7% (n = 10,995)	10.5% (n = 1,507)	5.1% (n = 730)	5.1% (n = 728)	2.6% (n = 380)
Bacterial/lobar pneumonia	96.1% (n = 13,778)	2.1% (n = 294)	1.2% (n = 168)	0.3% (n = 41)	0.4% (n = 59)
Cardiomediastinal abnormality	69.4% (n = 9,954)	16.1% (n = 2,305)	6.1% (n = 872)	6.1% (n = 880)	2.3% (n = 329)
Cardiomegaly	75.5% (n = 10,825)	16.1% (n = 2,312)	5.2% (n = 748)	2.9% (n = 421)	0.2% (n = 34)
Consolidation	94.8% (n = 13,592)	1.9% (n = 274)	1.2% (n = 168)	0.4% (n = 61)	1.7% (n = 245)
Edema	57.0% (n = 8,174)	26.3% (n = 3,766)	13.6% (n = 1,953)	2.7% (n = 392)	0.4% (n = 55)
Lesion	98.6% (n = 14,140)	1.4% (n = 200)	0.0% (n = 0)	0.0% (n = 0)	0.0% (n = 0)
Pleural effusion	91.7% (n = 13,146)	6.2% (n = 888)	1.8% (n = 259)	0.3% (n = 47)	0.0% (n = 0)
Pleural other	99.8% (n = 14,310)	0.2% (n = 30)	0.0% (n = 0)	0.0% (n = 0)	0.0% (n = 0)
Pneumothorax	99.8% (n = 14,309)	0.0% (n = 0)	0.0% (n = 0)	0.0% (n = 0)	0.2% (n = 31)
Rib fracture	96.3% (n = 13,805)	2.9% (n = 420)	0.0% (n = 0)	0.0% (n = 0)	0.8% (n = 115)
Shoulder fracture	97.1% (n = 13,920)	2.9% (n = 420)	0.0% (n = 0)	0.0% (n = 0)	0.0% (n = 0)
Support device hardware	71.5% (n = 10,250)	18.4% (n = 2,632)	4.4% (n = 631)	3.4% (n = 491)	2.3% (n = 336)

Supplementary Table 25 | Treatment effects of signed AI error ranges and overall treatment effect across ranges under binary ground truth labels. The treatment effects, 95% confidence intervals and p-values of different signed AI error ranges. The Wald test was used to test regression coefficients that estimate treatment effects against the null hypothesis of joint equality among treatment effects of different subgroups. Details of the statistical models are available in Methods. The Benjamini-Hochberg procedure was used to correct for multiple hypothesis testing over 15 individual pathologies. Underscore indicates a difference in the status of statistical significance and/or treatment effect trend from that of the same pathology under continuous ground truth probabilities. Non-applicable results are marked by N/A.

	AI signed error in [-100, -80]	AI signed error in (-80, -60]	AI signed error in (-60, -40]	AI signed error in (-40, -20]	AI signed error in (-20, 0]	AI signed error in [0, 20]	AI signed error in (20, 40]	*...... continued in the following table ......*
All pathologies aggregated	-2.792 (95% CI: -5.607 – 0.022)	1.900 (95% CI: -0.581 – 4.381)	8.087 (95% CI: 5.363 – 10.812)	9.574 (95% CI: 6.686 – 12.462)	2.102 (95% CI: 1.395 – 2.808)	0.410 (95% CI: 0.219 – 0.601)	-2.990 (95% CI: -3.936 – -2.043)	*...... continued in the following table ......*
<u>Abnormal</u>	N/A	-11.167 (95% CI: -12.817 – -9.516)	-1.786 (95% CI: -2.531 – -1.042)	6.029 (95% CI: -0.333 – 12.390)	4.802 (95% CI: 2.826 – 6.778)	N/A	4.851 (95% CI: 0.929 – 8.772)	*...... continued in the following*

								table ......
Airspace opacity	-2.777 (95% CI: -10.103 – 4.549)	1.828 (95% CI: -1.961 – 5.617)	8.434 (95% CI: 4.128 – 12.741)	9.558 (95% CI: 5.938 – 13.178)	18.340 (95% CI: 16.685 – 19.995)	1.336 (95% CI: 0.636 – 2.036)	-4.916 (95% CI: -6.387 – -3.444)	*...... continued in the following table ......*
Atelectasis	-0.896 (95% CI: -7.193 – 5.400)	5.322 (95% CI: 1.305 – 9.339)	13.204 (95% CI: 10.653 – 15.756)	N/A	N/A	0.168 (95% CI: -0.278 – 0.613)	-6.318 (95% CI: -8.425 – -4.212)	*...... continued in the following table ......*
Bacterial/lobar pneumonia	6.029 (95% CI: 4.964 – 7.095)	0.333 (95% CI: -1.199 – 1.865)	26.885 (95% CI: 25.710 – 28.060)	0.696 (95% CI: -0.170 – 1.562)	N/A	0.026 (95% CI: -0.346 – 0.399)	-6.818 (95% CI: -11.968 – -1.668)	*...... continued in the following table ......*
Cardiomediastinal abnormality	-4.481 (95% CI: -7.342 – -1.621)	0.872 (95% CI: -2.145 – 3.890)	3.548 (95% CI: -0.687 – 7.783)	11.264 (95% CI: 3.741 – 18.787)	13.821 (95% CI: 7.842 – 19.801)	1.410 (95% CI: 0.438 – 2.382)	-0.034 (95% CI: -2.283 – 2.214)	*...... continued in the following table ......*
Cardiomegaly	3.279 (95% CI: 1.364 – 5.193)	N/A (with data but uncomputable)	3.055 (95% CI: -1.752 – 7.861)	16.711 (95% CI: 11.512 – 21.909)	4.064 (95% CI: -0.610 – 8.738)	1.652 (95% CI: 0.854 – 2.449)	-0.309 (95% CI: -2.772 – 2.155)	*...... continued in the following table ......*
Consolidation	-3.571 (95% CI: -10.428 – 3.286)	8.549 (95% CI: 2.133 – 14.966)	21.240 (95% CI: 10.809 – 31.671)	-2.475 (95% CI: -3.158 – -1.793)	N/A	-0.227 (95% CI: -0.678 – 0.224)	-5.164 (95% CI: -9.970 – -0.358)	*...... continued in the following table ......*
Edema	N/A	N/A	6.677 (95% CI: -1.383 – 14.737)	6.579 (95% CI: 5.888 – 7.269)	3.153 (95% CI: -4.728 – 11.034)	0.497 (95% CI: -0.033 – 1.027)	-5.605 (95% CI: -7.048 – -4.161)	*...... continued in the following table ......*
Lesion	N/A	N/A	N/A	N/A	1.295 (95% CI: -0.225 – 2.814)	0.319 (95% CI: -0.026 – 0.663)	-5.354 (95% CI: -7.004 – -3.704)	*...... continued in the following table ......*
Pleural effusion	N/A	N/A	15.007 (95% CI: 13.776 – 16.239)	12.626 (95% CI: 7.180 – 18.072)	0.201 (95% CI: -0.145 – 0.547)	0.439 (95% CI: 0.029 – 0.848)	-1.870 (95% CI: -6.327 – 2.586)	*...... continued in the following table ......*
Pleural other	N/A	N/A	N/A	N/A	0.040 (95% CI: -0.132 – 0.213)	0.173 (95% CI: 0.014 – 0.331)	-2.771 (95% CI: -3.100 – -2.442)	*...... continued in the following table ......*
Pneumothorax	-14.365 (95% CI: -16.617 – -12.112)	N/A	N/A	N/A	0.279 (95% CI: -0.096 – 0.653)	0.067 (95% CI: -0.062 – 0.196)	N/A	*...... continued in the following table ......*
Rib fracture	-3.273 (95% CI: -15.358 – 8.813)	N/A	N/A	N/A	-0.323 (95% CI: -1.137 – 0.490)	0.201 (95% CI: -0.133 – 0.535)	1.259 (95% CI: -2.886 – 5.405)	*...... continued in the following table ......*
Shoulder fracture	N/A	N/A	N/A	N/A	0.104 (95% CI: -0.014 – 0.221)	0.164 (95% CI: -0.040 – 0.368)	0.658 (95% CI: -0.196 – 1.511)	*...... continued in the following table ......*
Support device hardware	-1.615 (95% CI: -7.976 – 4.745)	-4.000 (95% CI: -9.922 – 1.922)	2.554 (95% CI: -2.978 – 8.086)	9.471 (95% CI: 4.180 – 14.763)	13.967 (95% CI: 10.879 – 17.055)	0.724 (95% CI: 0.078 – 1.370)	-1.224 (95% CI: -3.533 – 1.084)	*...... continued in the following table ......*

Cont'd

	...... continued	AI signed error in (40, 60]	AI signed error in (60, 80]	AI signed error in (80, 100]	P-value for testing against joint equality	Benjamini-Hochberg adjusted p-value
All pathologies aggregated	*...... continued ......*	-5.920 (95% CI: -7.763 – -4.078)	-5.910 (95% CI: -8.022 – -3.798)	-8.535 (95% CI: -11.324 – -5.747)	2.29e-29, < 0.001	N/A
Abnormal	*...... continued ......*	2.381 (95% CI: 0.869 – 3.892)	-3.225 (95% CI: -5.098 – -1.351)	-7.560 (95% CI: -9.767 – -5.353)	5.00e-6, < 0.001	8.33e-6, < 0.001
Airspace opacity	*...... continued ......*	-10.677 (95% CI: -13.291 – -8.063)	-15.256 (95% CI: -21.614 – -8.898)	-35.322 (95% CI: -36.088 – -34.556)	1.000	1.071

Atelectasis	...... *continued*	-14.270 (95% CI: -23.291 – -5.250)	N/A	N/A	4.55e-40, < 0.001	2.27e-39, < 0.001
Bacterial/lobar pneumonia	...... *continued*	-14.506 (95% CI: -18.830 – -10.183)	N/A	N/A	< 0.001	< 0.001
Cardiomediastinal abnormality	...... *continued*	-8.834 (95% CI: -14.452 – -3.217)	-14.537 (95% CI: -15.065 – -14.009)	N/A	1.000	1.000
Cardiomegaly	...... *continued*	-7.069 (95% CI: -12.487 – -1.651)	-13.808 (95% CI: -18.497 – -9.119)	N/A	4.88e-6, < 0.001	9.14e-6, < 0.001
Consolidation	...... *continued*	-11.119 (95% CI: -16.400 – -5.838)	N/A	N/A	1.37e-7, < 0.001	2.94e-7, < 0.001
Edema	...... *continued*	-12.485 (95% CI: -15.202 – -9.769)	-16.804 (95% CI: -22.586 – -11.023)	-35.218 (95% CI: -36.073 – -34.364)	1.73e-147, < 0.001	2.588e-146, < 0.001
Lesion	...... *continued*	N/A	N/A	N/A	2.60e-14, < 0.001	6.49e-14, < 0.001
Pleural effusion	...... *continued*	-7.244 (95% CI: -7.794 – -6.693)	-23.019 (95% CI: -24.172 – -21.865)	N/A	1.000	1.154
Pleural other	...... *continued*	N/A	N/A	N/A	7.47e-60, < 0.001	5.60e-59, < 0.001
Pneumothorax	...... *continued*	N/A	N/A	N/A	1.87e-38, < 0.001	7.03e-38, < 0.001
Rib fracture	...... *continued*	N/A	N/A	N/A	0.457	0.571
Shoulder fracture	...... *continued*	N/A	N/A	N/A	0.402	0.548
Support device hardware	...... *continued*	-10.417 (95% CI: -18.311 – -2.523)	N/A (with data but uncomputable)	N/A	6.80e-15, < 0.001	2.04e-14, < 0.001

Supplementary Table 26 | Sample sizes and percentages of signed AI error ranges under binary ground truth labels. The sample sizes and percentages of radiologist predictions that fall into different signed AI error ranges for Supplementary Table 25.

	AI signed error in [-100, -80]	AI signed error in (-80, -60]	AI signed error in (-60, -40]	AI signed error in (-40, -20]	AI signed error in (-20, 0]	AI signed error in [0, 20]	AI signed error in (20, 40]	AI signed error in (40, 60]	AI signed error in (60, 80]	AI signed error in (80, 100]
All pathologies aggregated	0.8% (n = 1,767)	1.3% (n = 2,699)	1.3% (n = 2,709)	1.3% (n = 2,824)	5.7% (n = 12,214)	72.9% (n = 156,856)	8.4% (n = 18,043)	4.2% (n = 8,968)	2.8% (n = 5,928)	1.4% (n = 3,092)
Abnormal	0.0% (n = 0)	0.2% (n = 27)	0.3% (n = 50)	2.3% (n = 328)	16.2% (n = 2,323)	0.0% (n = 0)	5.3% (n = 761)	23.0% (n = 3,303)	31.8% (n = 4,566)	20.8% (n = 2,982)
Airspace opacity	0.9% (n = 133)	3.6% (n = 521)	6.3% (n = 899)	5.4% (n = 773)	0.2% (n = 34)	38.8% (n = 5,568)	28.3% (n = 4,060)	13.6% (n = 1,944)	2.5% (n = 353)	0.4% (n = 55)
Atelectasis	2.6% (n = 380)	5.4% (n = 773)	3.4% (n = 489)	0.0% (n = 0)	0.0% (n = 0)	76.7% (n = 10,995)	10.5% (n = 1,507)	1.4% (n = 196)	0.0% (n = 0)	0.0% (n = 0)
Bacterial/lobar pneumonia	0.4% (n = 59)	0.3% (n = 41)	0.3% (n = 41)	0.3% (n = 49)	0.0% (n = 0)	96.1% (n = 13,778)	1.7% (n = 245)	0.9% (n = 127)	0.0% (n = 0)	0.0% (n = 0)
Cardiomediastinal abnormality	2.7% (n = 382)	5.6% (n = 807)	2.0% (n = 293)	2.0% (n = 282)	0.8% (n = 110)	68.6% (n = 9,844)	13.9% (n = 1,987)	3.4% (n = 491)	1.0% (n = 144)	0.0% (n = 0)
Cardiomegaly	0.2% (n = 34)	0.6% (n = 81)	2.0% (n = 293)	0.6% (n = 86)	2.3% (n = 325)	73.2% (n = 10,500)	15.3% (n = 2,190)	3.4% (n = 491)	2.4% (n = 340)	0.0% (n = 0)
Consolidation	1.7% (n = 245)	0.4% (n = 61)	0.6% (n = 84)	0.3% (n = 49)	0.0% (n = 0)	94.8% (n = 13,592)	1.6% (n = 225)	0.6% (n = 84)	0.0% (n = 0)	0.0% (n = 0)

Edema	0.0% (n = 0)	0.0% (n = 0)	1.4% (n = 196)	0.3% (n = 43)	1.1% (n = 159)	55.9% (n = 8,015)	25.6% (n = 3,668)	12.6% (n = 1,812)	2.7% (n = 392)	0.4% (n = 55)
Lesion	0.0% (n = 0)	0.0% (n = 0)	0.0% (n = 0)	0.0% (n = 0)	1.3% (n = 193)	97.3% (n = 13,947)	1.4% (n = 200)	0.0% (n = 0)	0.0% (n = 0)	0.0% (n = 0)
Pleural effusion	0.0% (n = 0)	0.0% (n = 0)	0.4% (n = 54)	2.8% (n = 406)	18.7% (n = 2,681)	73.0% (n = 10,465)	3.4% (n = 482)	1.4% (n = 205)	0.3% (n = 47)	0.0% (n = 0)
Pleural other	0.0% (n = 0)	0.0% (n = 0)	0.0% (n = 0)	0.0% (n = 0)	34.5% (n = 4,953)	65.3% (n = 9,357)	0.2% (n = 30)	0.0% (n = 0)	0.0% (n = 0)	0.0% (n = 0)
Pneumothorax	0.2% (n = 31)	0.0% (n = 0)	0.0% (n = 0)	0.0% (n = 0)	0.6% (n = 89)	99.2% (n = 14,220)	0.0% (n = 0)	0.0% (n = 0)	0.0% (n = 0)	0.0% (n = 0)
Rib fracture	0.8% (n = 115)	0.0% (n = 0)	0.0% (n = 0)	0.0% (n = 0)	2.1% (n = 307)	94.1% (n = 13,498)	2.9% (n = 420)	0.0% (n = 0)	0.0% (n = 0)	0.0% (n = 0)
Shoulder fracture	0.0% (n = 0)	0.0% (n = 0)	0.0% (n = 0)	0.0% (n = 0)	2.1% (n = 307)	94.9% (n = 13,613)	2.9% (n = 420)	0.0% (n = 0)	0.0% (n = 0)	0.0% (n = 0)
Support device hardware	2.7% (n = 388)	2.7% (n = 388)	2.2% (n = 310)	5.6% (n = 808)	5.1% (n = 733)	66.0% (n = 9,464)	12.9% (n = 1,848)	2.2% (n = 315)	0.6% (n = 86)	0.0% (n = 0)

Supplementary Table 27 | Treatment effects on AUROC of subgroups split based on combined characteristics. The treatment effects, 95% confidence intervals and p-values of radiologists in binary subgroups split based on combined characteristics of years of experience, subspecialty in thoracic radiology and experience with AI tools. The Wald test was used to test regression coefficients that estimate treatment effects against the null hypothesis of joint equality among treatment effects of different subgroups. Details of the statistical models are available in Methods. The Benjamini-Hochberg procedure was used to correct for multiple hypothesis testing over 15 individual pathologies. Uncomputable results due to dataset size and statistical restrictions are marked by U (additional explanation for the causes is available in the Methods section and Supplementary Note | Statistical modeling for AUROC analysis). Non-applicable results are marked by N/A.

	Lower subgroup	Higher subgroup	P-value for testing against subgroup equality	Benjamini-Hochberg adjusted p-value
All pathologies aggregated	0.038 (95% CI: 0.023 – 0.052)	0.032 (95% CI: 0.021 – 0.043)	0.521	N/A
Abnormal	0.053 (95% CI: 0.014 – 0.092)	0.020 (95% CI: -0.013 – 0.052)	0.193	2.899
Airspace opacity	0.021 (95% CI: -0.010 – 0.052)	0.044 (95% CI: -0.006 – 0.094)	0.436	3.268
Atelectasis	U	U	U	U
Bacterial/lobar pneumonia	U	U	U	U
Cardiomediastinal abnormality	U	U	U	U
Cardiomegaly	U	U	U	U

Consolidation	U	U	U	U
Edema	U	U	U	U
Lesion	U	U	U	U
Pleural effusion	U	U	U	U
Pleural other	U	U	U	U
Pneumothorax	U	U	U	U
Rib fracture	U	U	U	U
Shoulder fracture	U	U	U	U
Support device hardware	U	U	U	U

Supplementary Table 28 | Treatment effects on AUROC of subgroups split based on years of experience. The treatment effects, 95% confidence intervals and p-values of radiologists in binary subgroups split based on years of experience. The Wald test was used to test regression coefficients that estimate treatment effects against the null hypothesis of joint equality among treatment effects of different subgroups. Details of the statistical models are available in Methods. The Benjamini-Hochberg procedure was used to correct for multiple hypothesis testing over 15 individual pathologies. Uncomputable results due to dataset size and statistical restrictions are marked by U (additional explanation for the causes is available in the Methods section and Supplementary Note | Statistical modeling for AUROC analysis). Non-applicable results are marked by N/A.

	Subgroup of less than or equal to 6 years of experience	Subgroup of more than 6 years of experience	P-value for testing against subgroup equality	Benjamini-Hochberg adjusted p-value
All pathologies aggregated	0.038 (95% CI: 0.025 – 0.052)	0.028 (95% CI: 0.017 – 0.040)	0.266	N/A
Abnormal	0.059 (95% CI: 0.024 – 0.094)	0.010 (95% CI: -0.025 – 0.045)	0.053	0.799
Airspace opacity	0.027 (95% CI: -0.019 – 0.074)	0.035 (95% CI: 0.003 – 0.068)	0.781	5.855
Atelectasis	U	U	U	U
Bacterial/lobar pneumonia	U	U	U	U
Cardiomediastinal abnormality	U	U	U	U
Cardiomegaly	U	U	U	U
Consolidation	U	U	U	U

Edema	U	U	U	U
Lesion	U	U	U	U
Pleural effusion	U	U	U	U
Pleural other	U	U	U	U
Pneumothorax	U	U	U	U
Rib fracture	U	U	U	U
Shoulder fracture	U	U	U	U
Support device hardware	U	U	U	U

Supplementary Table 29 | Treatment effects on AUROC of subgroups split based on subspecialty in thoracic radiology. The treatment effects, 95% confidence intervals and p-values of radiologists in binary subgroups split based on subspecialty in thoracic radiology. The Wald test was used to test regression coefficients that estimate treatment effects against the null hypothesis of joint equality among treatment effects of different subgroups. Details of the statistical models are available in Methods. The Benjamini-Hochberg procedure was used to correct for multiple hypothesis testing over 15 individual pathologies. Uncomputable results due to dataset size and statistical restrictions are marked by U (additional explanation for the causes is available in the Methods section and Supplementary Note | Statistical modeling for AUROC analysis). Non-applicable results are marked by N/A.

	Subgroup of radiologists that do not specialize in thoracic radiology	Subgroup of radiologists that specialize in thoracic radiology	P-value for testing against subgroup equality	Benjamini-Hochberg adjusted p-value
All pathologies aggregated	0.030 (95% CI: 0.019 – 0.041)	0.041 (95% CI: 0.026 – 0.056)	0.246	N/A
Abnormal	0.043 (95% CI: 0.017 – 0.070)	0.010 (95% CI: -0.050 – 0.069)	0.314	4.709
Airspace opacity	0.032 (95% CI: -0.003 – 0.067)	0.031 (95% CI: -0.014 – 0.075)	0.967	7.254
Atelectasis	U	U	U	U
Bacterial/lobar pneumonia	U	U	U	U
Cardiomediastinal abnormality	U	U	U	U
Cardiomegaly	U	U	U	U
Consolidation	U	U	U	U
Edema	U	U	U	U

Lesion	U	U	U	U
Pleural effusion	U	U	U	U
Pleural other	U	U	U	U
Pneumothorax	U	U	U	U
Rib fracture	U	U	U	U
Shoulder fracture	U	U	U	U
Support device hardware	U	U	U	U

Supplementary Table 30 | Treatment effects on AUROC of subgroups split based on experience with AI tools. The treatment effects, 95% confidence intervals and p-values of radiologists in binary subgroups split based on experience with AI tools. The Wald test was used to test regression coefficients that estimate treatment effects against the null hypothesis of joint equality among treatment effects of different subgroups. Details of the statistical models are available in Methods. The Benjamini-Hochberg procedure was used to correct for multiple hypothesis testing over 15 individual pathologies. Underscore indicates a difference in the status of statistical significance from that of the same pathology under absolute error and continuous ground truth probabilities. Uncomputable results due to dataset size and statistical restrictions are marked by U (additional explanation for the causes is available in the Methods section and Supplementary Note | Statistical modeling for AUROC analysis). Non-applicable results are marked by N/A.

	Subgroup of radiologists that do not have experience with AI tools	Subgroup of radiologists that have experience with AI tools	P-value for testing against subgroup equality	Benjamini-Hochberg adjusted p-value
All pathologies aggregated	0.039 (95% CI: 0.025 – 0.053)	0.028 (95% CI: 0.017 – 0.040)	0.266	N/A
Abnormal	0.029 (95% CI: -0.007 – 0.066)	0.039 (95% CI: 0.005 – 0.074)	0.690	5.178
Airspace opacity	0.063 (95% CI: 0.012 – 0.114)	0.004 (95% CI: -0.023 – 0.031)	0.045	0.679
Atelectasis	U	U	U	U
Bacterial/lobar pneumonia	U	U	U	U
Cardiomediastinal abnormality	U	U	U	U
Cardiomegaly	U	U	U	U
Consolidation	U	U	U	U
Edema	U	U	U	U

Lesion	U	U	U	U
Pleural effusion	U	U	U	U
Pleural other	U	U	U	U
Pneumothorax	U	U	U	U
Rib fracture	U	U	U	U
Shoulder fracture	U	U	U	U
Support device hardware	U	U	U	U

Supplementary Table 31 | Regression results for treatment effect on AUROC vs. unassisted AUROC. The regression coefficients, adjustment for attenuation bias and p-values of the analysis on the relationship between treatment effect on AUROC and unassisted AUROC. The Wald test was used to test regression coefficients against the null hypothesis of zero to determine in a continuous analysis if the independent variable is a predictor of the dependent variable. Details of the statistical models are available in Methods. The Benjamini-Hochberg procedure was used to correct for multiple hypothesis testing over 15 individual pathologies. Uncomputable results due to dataset size and statistical restrictions are marked by U (additional explanation for the causes is available in the Methods section and Supplementary Note | Statistical modeling for AUROC analysis). Non-applicable results are marked by N/A.

	Unassisted AUROC coefficient before adjustment for attenuation bias	Intercept coefficient before adjustment for attenuation bias	Unassisted AUROC coefficient after adjustment for attenuation bias	P-value for testing unassisted AUROC coefficient against 0	Benjamini-Hochberg adjusted p-value
All pathologies aggregated	-0.013 (95% CI: -0.237 – 0.212)	0.043 (95% CI: -0.153 – 0.238)	-0.039 (95% CI: -0.728 – 0.650)	0.911	N/A
Abnormal	U	U	U	U	U
Airspace opacity	U	U	U	U	U
Atelectasis	U	U	U	U	U
Bacterial/lobar pneumonia	U	U	U	U	U
Cardiomediastinal abnormality	U	U	U	U	U
Cardiomegaly	U	U	U	U	U
Consolidation	U	U	U	U	U
Edema	U	U	U	U	U
Lesion	U	U	U	U	U

Pleural effusion	U	U	U	U	U
Pleural other	U	U	U	U	U
Pneumothorax	U	U	U	U	U
Rib fracture	U	U	U	U	U
Shoulder fracture	U	U	U	U	U
Support device hardware	U	U	U	U	U

Supplementary Table 32 | Regression results for treatment effect on AUROC vs. unassisted AUROC without split sampling. The regression coefficients, adjustment for attenuation bias and p-values of the hypothetical analysis on the relationship between treatment effect on AUROC and unassisted AUROC without split sampling. The Wald test was used to test regression coefficients against the null hypothesis of zero to determine in a continuous analysis if the independent variable is a predictor of the dependent variable. Details of the statistical models are available in Methods. The Benjamini-Hochberg procedure was used to correct for multiple hypothesis testing over 15 individual pathologies. Uncomputable results due to dataset size and statistical restrictions are marked by U (additional explanation for the causes is available in the Methods section and Supplementary Note | Statistical modeling for AUROC analysis). Non-applicable results are marked by N/A.

	Unassisted AUROC coefficient before adjustment for attenuation bias	Intercept coefficient before adjustment for attenuation bias	Unassisted AUROC coefficient after adjustment for attenuation bias	P-value for testing unassisted AUROC coefficient against 0	Benjamini-Hochberg adjusted p-value
All pathologies aggregated	-0.460 (95% CI: -0.589 – -0.330)	0.424 (95% CI: 0.311 – 0.537)	-0.925 (95% CI: -1.189 – -0.662)	3.64e-12, < 0.001	N/A
Abnormal	-0.848 (95% CI: -1.031 – -0.664)	0.709 (95% CI: 0.558 – 0.859)	-5.318 (95% CI: -6.477 – -4.158)	1.22e-19, < 0.001	9.12e-19, < 0.001
Airspace opacity	-0.784 (95% CI: -0.918 – -0.650)	0.637 (95% CI: 0.530 – 0.745)	-2.370 (95% CI: -2.778 – -1.961)	1.74e-30, < 0.001	2.61e-29, < 0.001
Atelectasis	U	U	U	U	U
Bacterial/lobar pneumonia	U	U	U	U	U
Cardiomediastinal abnormality	U	U	U	U	U
Cardiomegaly	U	U	U	U	U
Consolidation	U	U	U	U	U
Edema	U	U	U	U	U
Lesion	U	U	U	U	U

Pleural effusion	U	U	U	U	U
Pleural other	U	U	U	U	U
Pneumothorax	U	U	U	U	U
Rib fracture	U	U	U	U	U
Shoulder fracture	U	U	U	U	U
Support device hardware	U	U	U	U	U

Supplementary Table 33 | Regression results for assisted AUROC vs. unassisted AUROC. The regression coefficients, adjustment for attenuation bias and p-values of the analysis on the relationship between assisted AUROC and unassisted AUROC. Underscore indicates a difference in the status of statistical significance from that of the same pathology under absolute error and continuous ground truth probabilities. The Wald test was used to test regression coefficients against the null hypothesis of zero to determine in a continuous analysis if the independent variable is a predictor of the dependent variable. Details of the statistical models are available in Methods. The Benjamini-Hochberg procedure was used to correct for multiple hypothesis testing over 15 individual pathologies. Uncomputable results due to dataset size and statistical restrictions are marked by U (additional explanation for the causes is available in the Methods section and Supplementary Note | Statistical modeling for AUROC analysis). Non-applicable results are marked by N/A.

	Unassisted AUROC coefficient before adjustment for attenuation bias	Intercept coefficient before adjustment for attenuation bias	Unassisted AUROC coefficient after adjustment for attenuation bias	P-value for testing unassisted AUROC coefficient against 0	Benjamini-Hochberg adjusted p-value
All pathologies aggregated	0.513 (95% CI: 0.382 – 0.644)	0.447 (95% CI: 0.333 – 0.560)	1.049 (95% CI: 0.779 – 1.319)	1.54e-14, < 0.001	N/A
<u>Abnormal</u>	0.108 (95% CI: -0.062 – 0.278)	0.745 (95% CI: 0.605 – 0.885)	0.579 (95% CI: -0.343 – 1.502)	0.214	3.213
Airspace opacity	U	U	U	U	U
Atelectasis	U	U	U	U	U
Bacterial/lobar pneumonia	U	U	U	U	U
Cardiomediastinal abnormality	U	U	U	U	U
Cardiomegaly	U	U	U	U	U
Consolidation	U	U	U	U	U
Edema	U	U	U	U	U
Lesion	U	U	U	U	U

Pleural effusion	U	U	U	U	U
Pleural other	U	U	U	U	U
Pneumothorax	U	U	U	U	U
Rib fracture	U	U	U	U	U
Shoulder fracture	U	U	U	U	U
Support device hardware	U	U	U	U	U

Supplementary Table 34 | Treatment effects on AUROC of absolute AI error ranges and overall treatment effect on AUROC across ranges under binary ground truth labels. The treatment effects on AUROC and 95% confidence intervals of different absolute AI error ranges and the statistics for the overall treatment effect on AUROC across ranges. The F-test was used to determine whether there is a statistically significant difference between treatment effects on AUROC in different bins. Specifically, we used the number of reads that fall into each bin as the group size. We used the grand mean AUROC and group AUROCs along with group sizes to compute the sum of squares between; we used the estimated standard error of each group AUROC along with the group size to compute the sum of squares within (error). Details of the statistical models are available in Methods. The Benjamini-Hochberg procedure was used to correct for multiple hypothesis testing over 15 individual pathologies. Underscore indicates a difference in the status of statistical significance and/or treatment effect trend from that of the same pathology under continuous ground truth probabilities and absolute error. Uncomputable results due to dataset size and statistical restrictions are marked by U (additional explanation for the causes is available in the Methods section and Supplementary Note | Statistical modeling for AUROC analysis). Non-applicable results are marked by N/A.

	AI absolute error in [0, 20]	AI absolute error in (20, 40]	AI absolute error in (40, 60]	AI absolute error in (60, 80]	AI absolute error in (80, 100]	P-value for testing against joint equality	Benjamini-Hochberg adjusted p-value	Across AI absolute error ranges
All pathologies aggregated	0.009 (95% CI: 0.003 – 0.016)	0.055 (95% CI: 0.038 – 0.073)	0.044 (95% CI: 0.020 – 0.069)	-0.039 (95% CI: -0.064 – -0.013)	-0.084 (95% CI: -0.108 – -0.061)	1.11e-16, < 0.001	N/A	0.024 (95% CI: 0.016 – 0.032)
Abnormal	U	0.112 (95% CI: 0.053 – 0.171)	-0.022 (95% CI: -0.157 – 0.113)	U	U	U	U	0.032 (95% CI: 0.011 – 0.053)
Airspace opacity	0.005 (95% CI: -0.082 – 0.092)	0.034 (95% CI: 0.001 – 0.066)	-0.014 (95% CI: -0.061 – 0.034)	-0.143 (95% CI: -0.221 – -0.064)	-0.222 (95% CI: -0.346 – -0.097)	1.11e-16, < 0.001	1.67e-15, < 0.001	0.022 (95% CI: -0.001 – 0.044)
Atelectasis	U	U	-0.028 (95% CI: -0.112 – 0.057)	U	U	U	U	0.045 (95% CI: 0.017 – 0.074)
Bacterial/lobar pneumonia	U	0.007 (95% CI: -0.111 – 0.126)	0.134 (95% CI: -0.064 – 0.333)	U	U	U	U	0.077 (95% CI: 0.010 – 0.143)
Cardiomediastinal abnormality	0.055 (95% CI: -0.014 – 0.124)	0.122 (95% CI: 0.064 – 0.179)	-0.018 (95% CI: -0.091 – 0.055)	-0.184 (95% CI: -0.287 – -0.082)	U	U	U	0.038 (95% CI: 0.014 – 0.062)
Cardiomegaly	0.044 (95% CI: -0.024 – 0.113)	0.156 (95% CI: 0.062 – 0.251)	-0.006 (95% CI: -0.087 – 0.076)	-0.070 (95% CI: -0.195 – 0.055)	U	U	U	0.052 (95% CI: 0.014 – 0.090)
Consolidation	U	0.011 (95% CI: -0.091 – 0.113)	0.122 (95% CI: -0.030 – 0.275)	U	U	U	U	0.036 (95% CI: -0.010 – 0.082)

Edema	0.046 (95% CI: -0.135 – 0.227)	0.022 (95% CI: -0.071 – 0.115)	-0.032 (95% CI: -0.126 – 0.062)	U	U	U	U	0.023 (95% CI: -0.031 – 0.077)
Lesion	U	U	N/A	N/A	N/A	U	U	U
Pleural effusion	0.016 (95% CI: -0.016 – 0.047)	0.082 (95% CI: 0.018 – 0.146)	0.020 (95% CI: -0.153 – 0.193)	U	N/A	U	U	0.047 (95% CI: 0.010 – 0.084)
Pleural other	U	U	N/A	N/A	N/A	U	U	U
Pneumothorax	U	N/A	N/A	N/A	U	U	U	-0.100 (95% CI: -0.289 – 0.089)
Rib fracture	U	U	N/A	N/A	U	U	U	-0.017 (95% CI: -0.125 – 0.091)
Shoulder fracture	U	U	N/A	N/A	N/A	U	U	N/A
Support device hardware	0.039 (95% CI: 0.017 – 0.062)	0.028 (95% CI: -0.004 – 0.060)	-0.008 (95% CI: -0.066 – 0.050)	-0.042 (95% CI: -0.135 – 0.052)	U	U	U	0.028 (95% CI: 0.010 – 0.045)

Supplementary Table 35 | Treatment effects on AUROC of signed AI error ranges and overall treatment effect on AUROC across ranges under binary ground truth labels. The treatment effects on AUROC and 95% confidence intervals of different signed AI error ranges and the statistics for the overall treatment effect on AUROC across ranges. Underscore indicates a difference in the status of statistical significance and/or treatment effect trend from that of the same pathology under continuous ground truth probabilities and absolute error. Uncomputable results due to dataset size and statistical restrictions are marked by U (additional explanation for the causes is available in the Methods section and Supplementary Note | Statistical modeling for AUROC analysis). Each investigated pathology is found to be not present (defined as having a ground truth probability of less than or equal to 50) in all patient cases where the AI predicted probability overestimates the pathology probability. This causes AUROC to be undefined in all positive bins. Each investigated pathology is found to be present (defined as having a ground truth probability of greater than 50) in all patient cases where the AI predicted probability underestimates by at least 20. This causes AUROC to be undefined in the corresponding negative bins. Non-applicable results are marked by N/A.

	AI signed error in [-100, -80]	AI signed error in (-80, -60]	AI signed error in (-60, -40]	AI signed error in (-40, -20]	AI signed error in (-20, 0]	AI signed error in [0, 20]	AI signed error in (20, 40]	*...... continued in the following table ......*
All pathologies aggregated	U	U	U	U	0.002 (95% CI: -0.002 – 0.007)	U	U	*...... continued in the following table ......*
Abnormal	N/A	U	U	U	U	N/A	U	*...... continued in the following table ......*
Airspace opacity	U	U	U	U	U	U	U	*...... continued in the following table ......*
Atelectasis	U	U	U	N/A	N/A	U	U	*...... continued in the following table ......*
Bacterial/lobar pneumonia	U	U	U	U	N/A	U	U	*...... continued in the following table ......*

Cardiomediastinal abnormality	U	U	U	U	U	U	U	...... *continued in the following table* ......
Cardiomegaly	U	U	U	U	0.018 (95% CI: -0.039 - 0.074)	U	U	...... *continued in the following table* ......
Consolidation	U	U	U	U	N/A	U	U	...... *continued in the following table* ......
Edema	N/A	N/A	U	U	0.009 (95% CI: -0.166 – 0.183)	U	U	...... *continued in the following table* ......
Lesion	N/A	N/A	N/A	N/A	U	U	U	...... *continued in the following table* ......
Pleural effusion	N/A	N/A	U	U	0.013 (95% CI: -0.012 – 0.039)	U	U	...... *continued in the following table* ......
Pleural other	N/A	N/A	N/A	N/A	U	U	U	...... *continued in the following table* ......
Pneumothorax	U	N/A	N/A	N/A	U	U	N/A	...... *continued in the following table* ......
Rib fracture	U	N/A	N/A	N/A	U	U	U	...... *continued in the following table* ......
Shoulder fracture	N/A	N/A	N/A	N/A	U	U	U	...... *continued in the following table* ......
Support device hardware	U	U	U	U	U	U	U	...... *continued in the following table* ......

Cont'd

	...... *continued*	AI signed error in (40, 60]	AI signed error in (60, 80]	AI signed error in (80, 100]	P-value for testing against joint equality	Benjamini-Hochberg adjusted p-value
All pathologies aggregated	...... *continued*	U	U	U	U	N/A
Abnormal	...... *continued*	U	U	U	U	U
Airspace opacity	...... *continued*	U	U	U	U	U
Atelectasis	...... *continued*	U	N/A	N/A	U	U
Bacterial/lobar pneumonia	...... *continued*	U	N/A	N/A	U	U
Cardiomediastinal abnormality	...... *continued*	U	U	N/A	U	U
Cardiomegaly	...... *continued*	U	U	N/A	U	U
Consolidation	...... *continued*	U	N/A	N/A	U	U
Edema	...... *continued*	U	U	U	U	U
Lesion	...... *continued*	N/A	N/A	N/A	U	U

Pleural effusion	...... *continued*	U	U	N/A	U	U
Pleural other	...... *continued*	N/A	N/A	N/A	U	U
Pneumothorax	...... *continued*	N/A	N/A	N/A	U	U
Rib fracture	...... *continued*	N/A	N/A	N/A	U	U
Shoulder fracture	...... *continued*	N/A	N/A	N/A	U	U
Support device hardware	...... *continued*	U	U	N/A	U	U

Supplementary Table 36 | Treatment effects on AUROC of subgroups split based on years of experience computed using the Obuchowski and Rockette analysis. The treatment effects, 95% confidence intervals and p-values of radiologists in binary subgroups split based on years of experience. We used the treatment effects and standard errors outputted by the Obuchowski and Rockette analysis for subgroups to estimate the standard errors of the difference in treatment effects, while assuming a covariance of zero. We then ran the Wald test for testing the difference against the null hypothesis of zero. Uncomputable results due to dataset size and statistical restrictions are marked by U (additional explanation for the causes is available in the Methods section and Supplementary Note | Statistical modeling for AUROC analysis). Non-applicable results are marked by N/A.

	Subgroup of less than or equal to 6 years of experience	Subgroup of more than 6 years of experience	P-value for testing against subgroup equality	Benjamini-Hochberg adjusted p-value
All pathologies aggregated	0.038 (95% CI: 0.024 – 0.052)	0.028 (95% CI: 0.016 – 0.040)	0.266	N/A
Abnormal	U	U	U	U
Airspace opacity	U	U	U	U
Atelectasis	U	U	U	U
Bacterial/lobar pneumonia	U	U	U	U
Cardiomediastinal abnormality	U	U	U	U
Cardiomegaly	U	U	U	U
Consolidation	U	U	U	U
Edema	U	U	U	U
Lesion	U	U	U	U
Pleural effusion	U	U	U	U
Pleural other	U	U	U	U

Pneumothorax	U	U	U	U
Rib fracture	U	U	U	U
Shoulder fracture	U	U	U	U
Support device hardware	U	U	U	U

Supplementary Table 37 | Treatment effects on AUROC of subgroups split based on subspecialty in thoracic radiology computed using the Obuchowski and Rockette analysis. The treatment effects, 95% confidence intervals and p-values of radiologists in binary subgroups split based on subspecialty in thoracic radiology. We used the treatment effects and standard errors outputted by the Obuchowski and Rockette analysis for subgroups to estimate the standard errors of the difference in treatment effects, while assuming a covariance of zero. We then ran the Wald test for testing the difference against the null hypothesis of zero. Uncomputable results due to dataset size and statistical restrictions are marked by U (additional explanation for the causes is available in the Methods section and Supplementary Note | Statistical modeling for AUROC analysis). Non-applicable results are marked by N/A.

	Subgroup of radiologists that do not specialize in thoracic radiology	Subgroup of radiologists that specialize in thoracic radiology	P-value for testing against subgroup equality	Benjamini-Hochberg adjusted p-value
All pathologies aggregated	0.030 (95% CI: 0.019 – 0.041)	0.041 (95% CI: 0.026 – 0.057)	0.247	N/A
Abnormal	U	0.009 (95% CI: -0.053 – 0.072)	U	U
Airspace opacity	U	U	U	U
Atelectasis	U	U	U	U
Bacterial/lobar pneumonia	U	U	U	U
Cardiomediastinal abnormality	U	U	U	U
Cardiomegaly	U	U	U	U
Consolidation	U	U	U	U
Edema	U	U	U	U
Lesion	U	U	U	U
Pleural effusion	U	U	U	U
Pleural other	U	U	U	U
Pneumothorax	U	U	U	U

Rib fracture	U	U	U	U
Shoulder fracture	U	U	U	U
Support device hardware	U	0.064 (95% CI: 0.001 – 0.128)	U	U

Supplementary Table 38 | Treatment effects on AUROC of subgroups split based on experience with AI tools computed using the Obuchowski and Rockette analysis. The treatment effects, 95% confidence intervals and p-values of radiologists in binary subgroups split based on experience with AI tools. We used the treatment effects and standard errors outputted by the Obuchowski and Rockette analysis for subgroups to estimate the standard errors of the difference in treatment effects, while assuming a covariance of zero. We then ran the Wald test for testing the difference against the null hypothesis of zero. Uncomputable results due to dataset size and statistical restrictions are marked by U (additional explanation for the causes is available in the Methods section and Supplementary Note | Statistical modeling for AUROC analysis). Non-applicable results are marked by N/A.

	Subgroup of radiologists that do not have experience with AI tools	Subgroup of radiologists that have experience with AI tools	P-value for testing against subgroup equality	Benjamini-Hochberg adjusted p-value
All pathologies aggregated	0.039 (95% CI: 0.025 – 0.053)	0.028 (95% CI: 0.017 – 0.040)	0.265	N/A
Abnormal	U	U	U	U
Airspace opacity	U	U	U	U
Atelectasis	U	U	U	U
Bacterial/lobar pneumonia	U	U	U	U
Cardiomediastinal abnormality	U	U	U	U
Cardiomegaly	U	U	U	U
Consolidation	U	U	U	U
Edema	U	U	U	U
Lesion	U	U	U	U
Pleural effusion	U	U	U	U
Pleural other	U	U	U	U
Pneumothorax	U	U	U	U
Rib fracture	U	U	U	U
Shoulder fracture	U	U	U	U

Support device hardware	U	0.029 (95% CI: -0.001 – 0.059)	U	U

Supplementary Table 39 | Sex and gender statistics of participating radiologists. Information is collected through the survey question “How do you identify?” with four possible answer options. There are 136 radiologists with available survey data.

Female	42
Male	83
Other	1
Prefer not to answer	10

Supplementary Table 40 | Sex and gender statistics of patient cases. Information is processed from the corresponding indications of the patient cases. There are 324 patient cases in total.

Female	131
Male	151
Unknown	42

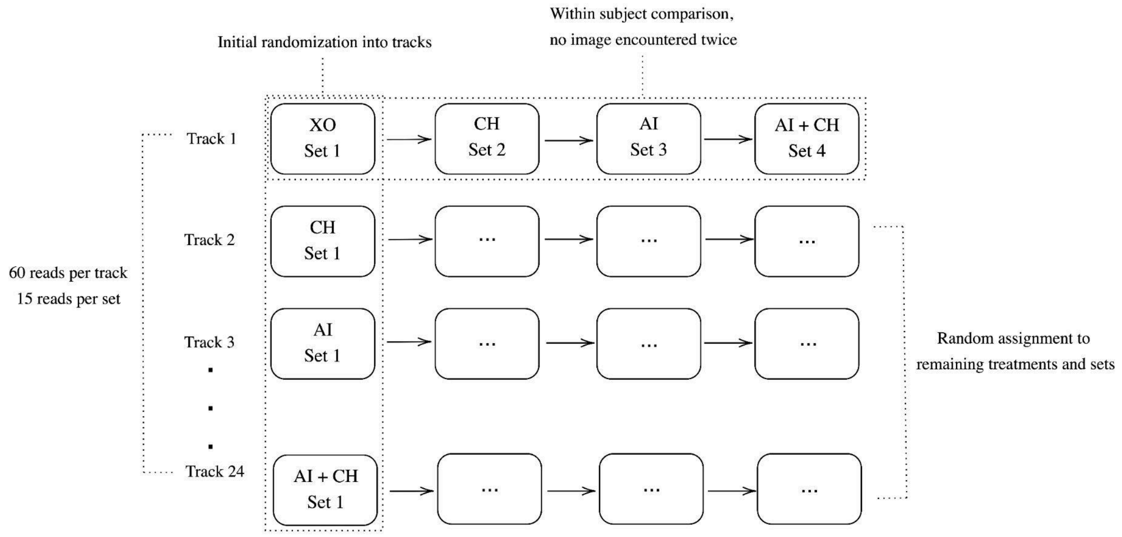

Supplementary Fig. 1 | Non-repeated-measure design. Illustration of the data collection process in the non-repeated-measure design.

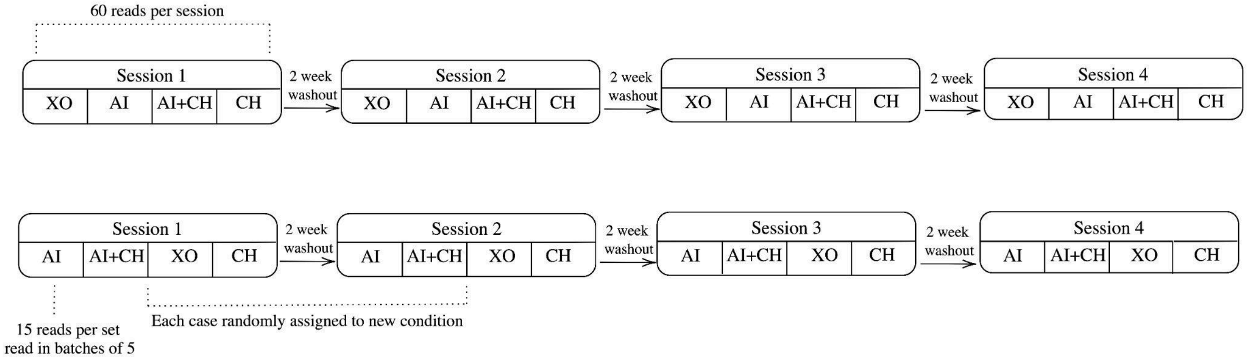

Supplementary Fig. 2 | Repeated-measure design. Illustration of the data collection process in the repeated-measure design.

Supplementary Note | Statistical modeling for AUROC analysis. Description of the statistical modeling methods used for the AUROC analysis.

Subgroup-specific treatment effect models

For the analyses on experience-based radiologist characteristics, we computed the treatment effects of subgroups split based on the predictor of interest by building a linear regression model with the following formulation using the statsmodels library: *AUROC ~ 1 + C(subgroup) + C(treatment):C(subgroup)*. Here, each data point corresponds to a radiologist. AUROC refers to the AUROC of the radiologist's predictions, 1 refers to an intercept term, subgroup refers to an indicator of the subgroup the radiologist is split into, and treatment refers to a binary indicator of whether the predictions are made with or without AI assistance. This formulation allows us to compute the subgroup-specific treatment effect of AI assistance for both non-repeated-measure data and repeated-measure data. We computed cluster-robust standard errors clustered at the radiologist level.

A subgroup analysis based on AI error, which has been shown for the absolute AI error and signed AI error, is not applicable to the AUROC analysis. This is because AUROC is an aggregate metric computed over a set of patient cases and therefore cannot be computed at the observation level or patient case level.

Split sampling construction to avoid reversion to the mean

To study unassisted AUROC as a predictor of treatment effect on AUROC, we built a linear regression model with the following formulation using the statsmodels library: *treatment effect on AUROC ~ 1 + unassisted AUROC*.

For the non-repeated-measure design, we randomly split the unassisted patient cases into two sets of the same size. We used one set of unassisted cases to compute the unassisted AUROC performance of the radiologist. We used the other set of unassisted cases and all assisted cases to compute the treatment effect on AUROC, which is defined as the difference between the unassisted AUROC on the disjoint set of unassisted cases and the assisted AUROC.

For the repeated-measure design, we randomly split the patient cases into two sets of the same size. We used one set of patient cases to compute the unassisted AUROC performance of the radiologist. We used the other set of patient cases to compute the treatment effect on AUROC, which is defined as the difference between the unassisted AUROC and the assisted AUROC on the disjoint set of cases.

To study unassisted AUROC as a predictor of assisted AUROC, we built a linear regression model with the following formulation using the statsmodels library: *assisted AUROC~ 1 + unassisted AUROC*.

For the non-repeated-measure design, because the unassisted and assisted patient cases are disjoint, we used all unassisted cases to compute the unassisted AUROC performance and all assisted cases to compute the assisted AUROC performance of the radiologist.

For the repeated-measure design, we randomly split the patient cases into two sets of the same size. We used one set of patient cases to compute the unassisted AUROC performance of the radiologist.

We used the other set of patient cases to compute the assisted AUROC performance of the radiologist.

Adjustment for attenuation bias

We adjusted for attenuation bias for the AUROC analysis on the relationship between treatment effect on AUROC and unassisted AUROC, and assisted AUROC and unassisted AUROC.

We want to estimate regressions of the form:

$$Y_r = \beta_0 + \beta_1 E[m_r] + \varepsilon_r$$

where Y_r is an outcome for radiologist r and $E[m_r]$ is radiologist r's unassisted AUROC performance. We observe a noisy version of the unassisted AUROC $\tilde{m}_r = E[m_r] + \eta_r$, where $E[\eta_r m_r] = 0$ and $E[\eta_r \varepsilon_r] = 0$, which are justified by independent and identically distributed (i.i.d.) sampling of cases and split sampling respectively.

Using observations from the experiment, we estimate the following regression:

$$Y_r = \gamma_0 + \gamma_1 \tilde{m}_r + \varepsilon_r$$

Recall that

$$\hat{\gamma}_1 \to^p \frac{E[(m_r+\eta_r-E[m_r])(Y_r-E[Y_r])]}{E[(m_r+\eta_r-E[m_r])^2]} = \frac{E[(m_r-E[m_r])(Y_r-E[Y_r])]}{E[(m_r-E[m_r])^2]+E[\eta_r^2]} = \beta_1\lambda$$

where $\lambda = \frac{E[(m_r-E[m_r])^2]}{E[(m_r-E[m_r])^2]+E[\eta_r^2]}$ and $\beta_1 = \frac{E[(m_r-E[m_r])(Y_r-E[Y_r])]}{E[(m_r-E[m_r])^2]}$. We can estimate λ using a plug-in estimator for each term in the data: (1) We estimate $E[\eta_r^2]$ using $E[s.e.(\tilde{m}_r)^2]$. (2) $E[(m_r - E[m_r])^2] = E[(\tilde{m}_r - E[\tilde{m}_r])^2] - E[\eta_r^2]$, which can be estimated by taking the difference between the variance of the observed $\tilde{m}_r$'s and the estimated $E[\eta_r^2]$. The denominator of λ is effectively $E[(\tilde{m}_r - E[\tilde{m}_r])^2]$.

Finally, with $\hat{\lambda}$, we can estimate β_1 using the estimator $\hat{\beta}_1 = \hat{\gamma}_1/\hat{\lambda}$.

For inference, notice that $\sqrt{n}(\hat{\gamma}_1 - \gamma_1) \to^d N(0, \sigma_\gamma^2)$ and $\hat{\lambda} \to^p \lambda$. By Slutsky's theorem, we know that $\sqrt{n}\frac{(\hat{\gamma}-\gamma)}{\hat{\lambda}} \to^d N\left(0, \frac{\sigma_\gamma^2}{\lambda^2}\right)$. Therefore, we divide the standard errors of $\hat{\gamma}_1$ by $\hat{\lambda}$ to obtain the standard errors of $\hat{\beta}_1$.

This concludes the adjustment for attenuation bias for the slope term.

Standard error of AUROC

We used the se_auc implementation from the R package auctestr (v1.0.0) to compute the standard error of AUROC. This implementation estimates standard error based on the AUROC metric value and the number of positive and negative cases.

In Supplementary Table 34, we estimated the standard error of the treatment effect on AUROC by assuming independence between the standard error of the unassisted AUROC and assisted AUROC

and computing the square root of the sum of the squared standard errors of the unassisted and assisted AUROCs. The 95% confidence interval of the treatment effect on AUROC was then computed based on the normal distribution:
$CI = (t - c.d.f.(1-0.05/2) \times s.e.(t), t + c.d.f.(1-0.05/2) \times s.e.(t))$.

Obuchowski and Rockette analysis

As a sanity check on the statistical models proposed, we additionally applied the Obushowski and Rockette analysis[1], which allowed us to compare the AUROC performance of radiologists with and without AI assistance under a multi-reader, multi-case setting and compute the treatment effect of AI on AUROC, to perform the analysis of experienced-based characteristics being potential predictors of treatment effect. Specifically, we applied the Obuchowski and Rockette analysis on each subgroup of data, as defined by years of experience, specialty in thoracic radiology or experience with AI tools, computed the treatment effect on AUROC in each subgroup, and compared the subgroup-specific treatment effects. We found consistent results as those computed from using the statistical models proposed (Supplementary Table 36-38). We used the R package MRMCaov (v0.3.0)[2-3].

Numerical challenges and statistical restrictions for AUROC analysis

Because AUROC is an aggregate metric over a set of patient cases and is undefined for a single case, the analysis of how the quality of AI assistance affects treatment effect cannot be repeated with AUROC as the performance metric for AI quality, because said analysis requires case-level decisions on whether a case has a relatively accurate AI prediction or a relatively inaccurate AI prediction.

Given the properties of AUROC, dataset size, and generally low prevalence of pathologies, using AUROC as a metric poses significant challenges, in addition to the ones faced with using an observation-level metric such as absolute error. For instance, some radiologists only reviewed negative patient cases of a pathology, under which scenario AUROC would be undefined. These radiologists also could not be excluded from the analysis post-hoc, because such an exclusion would bias the data distribution. This situation would be further exacerbated with split sampling, where only subsets of patient cases of a radiologist could be considered. As a result of these issues, AUROC analyses could not be performed on many pathologies. Additionally, because AUROC is a radiologist-level aggregate metric, we lose statistical power in terms of the number of observations we can use to test hypotheses.

All computable results have been added to Supplementary Table 27-36, while uncomputable ones are marked accordingly.

Supplementary Note | Participant recruitment and affiliation. Description of the participating radiologists in the experiment.

Participating radiologists in the non-repeated-measure design were recruited from teleradiology companies. The radiologists are duly licensed Medical Doctors (MDs) in India and many of them are licensed to practice in the United Kingdom. They trained under US board-certified radiologists from the teleradiology companies and are monitored for quality control by a US board-certified team of radiologists from the teleradiology companies. They follow US guidelines for reporting and quality.

Participating radiologists were compensated with a piece-rate. Additionally, they were offered monetary incentives for providing their best estimates, as shown in page 8 of Supplementary Note | Experiment interface and instructions. Investigators did not directly interact with the radiologists except answering questions about the experiment or technical support, and were blinded to the radiologists' treatment condition setup when the experiment was in progress.

The repeated-measure design required radiologists' continued participation over a few months because of the installation of washout periods between sessions. For the repeated-measure design, staff radiologists from the VinMec healthcare system in Vietnam were recruited.

The information listed above and additional details about participants can be found in a separate study[4].

Details about the institutions of the participating radiologists in the non-repeated-measure design are listed in Supplementary Table A.1 and Supplementary Table A.2. Details about the institutions of the participating radiologists in the repeated-measure design are listed in Supplementary Table A.3 and Supplementary Table A.4.

Supplementary Table A.1 | Size and type of primary affiliation for participating radiologists in the non-repeated-measure design. Information is collected through the survey question "What best describes your primary affiliation?" with four possible answer options. There are 107 radiologists in the non-repeated-measure design.

Large clinical setting	48
Medium clinical setting	41
Small clinical setting	17
Non-clinical affiliation	1

Supplementary Table A.2 | Academic hospital affiliation for participating radiologists in the non-repeated-measure design. Information is collected through the survey question "Are you affiliated with an academic hospital?" with two possible answer options. There are 107 radiologists in the non-repeated-measure design.

Yes	69
No	38

Supplementary Table A.3 | Size and type of primary affiliation for participating radiologists in the repeated-measure design. Information is collected through the survey question "What best describes your primary affiliation?" with four possible answer options. There are 33 radiologists in the repeated-measure design.

Large clinical setting	19
Medium clinical setting	7
Small clinical setting	3
Non-clinical affiliation	0
No available survey data	4

Supplementary Table A.4 | Academic hospital affiliation for participating radiologists in the repeated-measure design. Information is collected through the survey question "Are you affiliated with an academic hospital?" with two possible answer options. There are 33 radiologists in the repeated-measure design.

Yes	21
No	8
No available survey data	4

Supplementary Note | Experiment interface and instructions. A complete walkthrough of the experiment interface and instructions that participating radiologists received in the experiment[4]. Participants could navigate from one page to the next by clicking on a "Next" button. Text in italics represents comments on the experiment interface and instructions and they were not presented to the participants.

Page 1

Instructions

You are about to participate in a study on medical decision making. You may pause the study at any time. To resume, revisit the link you were given and your progress will have been saved.

We will present you with adult patients with potential thoracic pathologies. These patients will be presented under the following four scenarios:

1. Only a chest X-ray is shown.
2. An X-ray is accompanied with additional information about the clinical history
3. An X-ray is shown along with Artificial Intelligence (AI) support. This AI tool is described in further detail below.
4. An X-ray is shown along with both additional information on clinical history and the AI support.

The patients are randomly assigned to each of these scenarios. That is, availability of clinical history and/or AI support is unrelated to the patient.

Clinical History: includes available lab results or indications by the treating physician, if any.

AI support: This tool uses only the X-ray image to predict the probability of each potential pathology of interest. The tool is based on state-of-the-art machine learning algorithms developed by a leading team of researchers at Stanford University.

Responses

For each patient and pathology, we will ask for both an assessment and a treatment decision:

1. We will first ask for your assessment of the *probability* that each condition is present in a patient. **Please consider all pathologies and findings that would be relevant in a radiology report for the patient. You should express your uncertainty about the presence of one or many conditions by appropriately choosing the probability.**
   Note that it is possible that the patient has multiple such conditions or none of them.
2. If you determine that a pathology may be present, we may ask you to rate the severity and/or extent of the disease on a scale.
3. Finally, when relevant we will ask whether you would recommend treatment or follow up according to the clinical standard of care if you determine that the pathology may be present.

The first two responses are diagnostic while the third is a clinical decision. We are aware that a single physician or radiologist typically does not perform both tasks. However, for this study, we ask that you respond to the best of your ability in both of these roles.

Browser Compatibility

This platform supports desktop versions of Chrome, Firefox, and Edge. Important features on non-supported browsers (including Safari) are missing and we discourage their use for this experiment. In addition, the platform does not support any mobile devices and the platform will perform poorly on mobile. If you encounter any issues during the experiment, please send an email to DiagnosticAI@mit.edu and we will follow-up quickly.

Page 2

Clinical Hierarchy

The interface uses a *hierarchy* to categorize various thoracic conditions. It will be useful to familiarize yourself with this hierarchy before you start, but you may also revisit the hierarchy at any time throughout the experiment by clicking the help tab in the upper right corner.

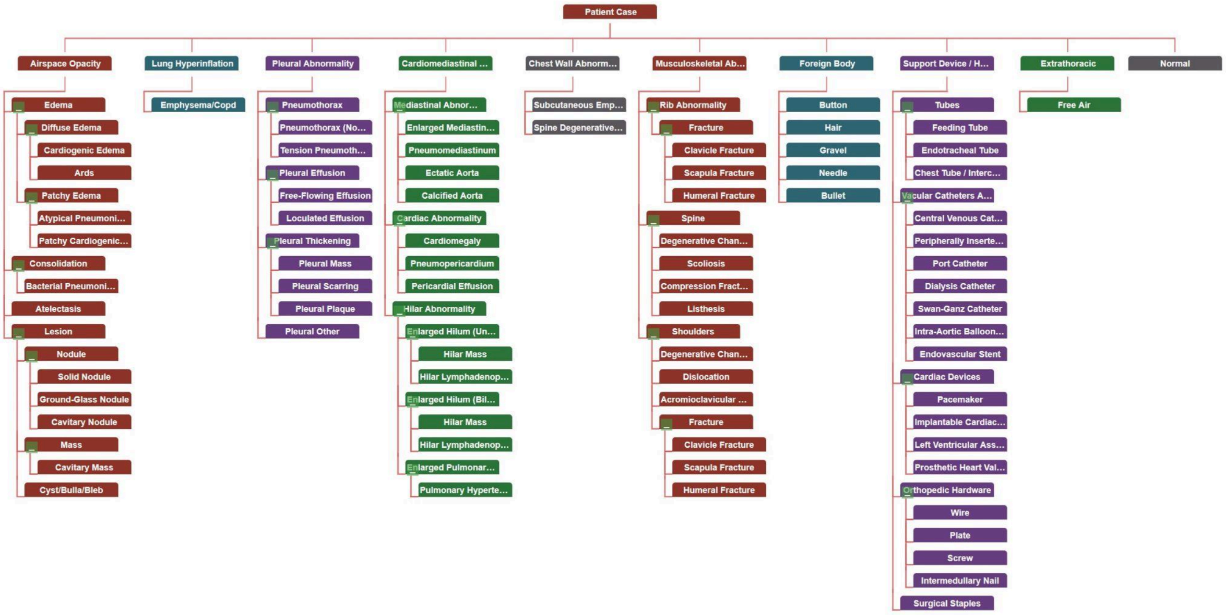

Supplementary Fig. B.1 | Hierarchy of pathological findings. *This visualization of the hierarchy was presented to participating radiologists. The hierarchy was also used to organize the diagnoses radiologists needed to provide.*

Page 3

AI Support Tool

The AI support tool that is provided uses only the X-ray image to predict the probability of each potential pathology of interest. The tool is based on

state-of-the-art machine learning algorithms developed by a leading team of researchers at Stanford University. **The tool is trained only on X-ray images, meaning it does not incorporate the clinical history of the patients.**

Performance of the AI Support Tool

The AI tool is described in Irvin et al. [2019][1], which showed the **AI tool performed at or near expert levels** across the pathologies studied. Below we plot two measures of performance of the AI tool. We plot in blue the accuracy of the tool, defined as the share of cases correctly diagnosed when treating false positives and false negatives equally. In red, we plot the Area Under the ROC curve (AUC), which is another measure of AI classification performance. The AUC is a number between 0 and 100%, with numbers close to 100% representing better algorithm performance. The AUC is equal to the probability that a randomly chosen positive case is ranked higher than a randomly chosen negative case.

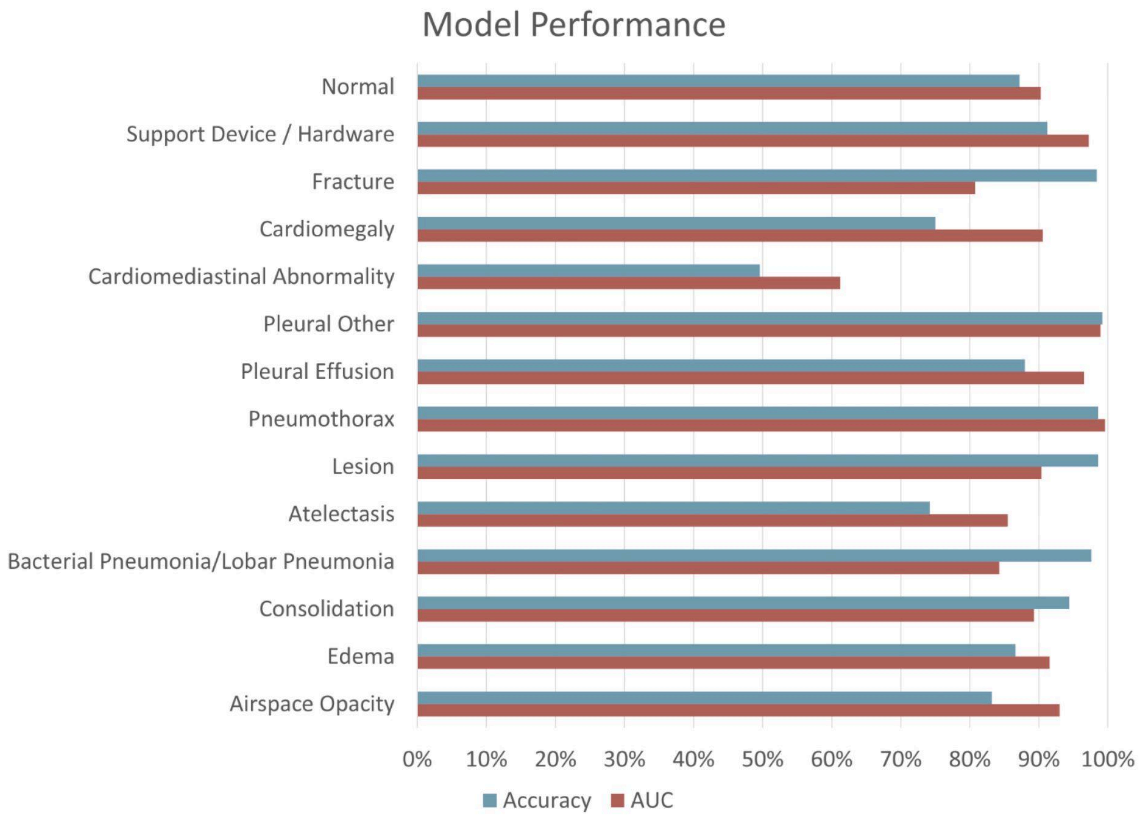

Supplementary Fig. B.2 | AUC and accuracy of AI model. *Bar plot of the AUC and accuracy performance of the model that generates AI predictions, over 14 findings.*

Example Images

Below are 50 example images with the associated AI tool predictions. These images are randomly chosen to allow you to familiarize yourself with the AI support tool and its accuracy.

Example 1

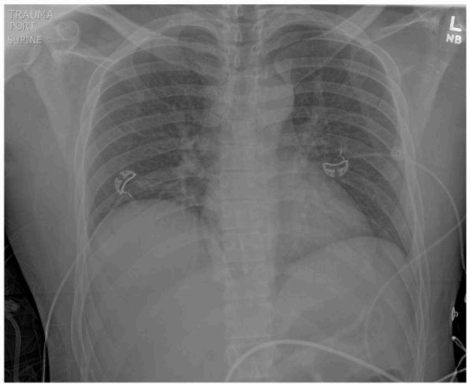

Pathology	AI Prediction
Airspace Opacity	16%
• Edema	7%
• Consolidation	3%
◦ Bacterial Pneumonia/Lobar Pneumonia	3%
• Atelectasis	9%
• Lesion	4%
Pleural Abnormality	
• Pneumothorax	7%
• Pleural Effusion	1%
• Pleural Other	0%
Cardiomediastinal Abnormality	14%
◦ Cardiomegaly	1%
Musculoskeletal Abnormality	
◦ Fracture	9%
Support Device / Hardware	12%
Normal	47%

Example 2

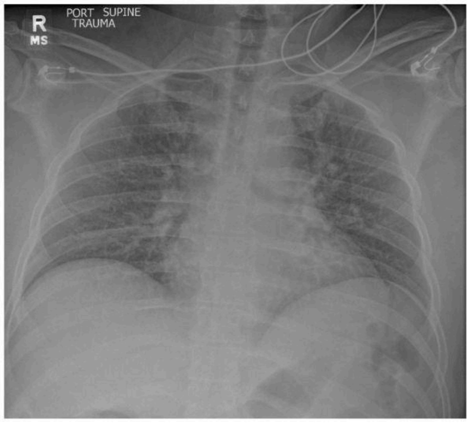

Pathology	AI Prediction
Airspace Opacity	42%
• Edema	42%
• Consolidation	14%
◦ Bacterial Pneumonia/Lobar Pneumonia	14%
• Atelectasis	5%
• Lesion	5%
Pleural Abnormality	
• Pneumothorax	3%
• Pleural Effusion	0%
• Pleural Other	1%
Cardiomediastinal Abnormality	16%
◦ Cardiomegaly	5%
Musculoskeletal Abnormality	
◦ Fracture	9%
Support Device / Hardware	3%
Normal	21%

Supplementary Fig. B.3 | Example chest X-rays with AI predictions. *Two out of 50 example chest X-rays juxtaposed with the corresponding AI predictions.*

We omit the remaining 48 examples for brevity. The remaining examples follow the same format as the examples shown above.

1. Irvin, Jeremy, et al. "Chexpert: A large chest radiograph dataset with uncertainty labels and expert comparison." Proceedings of the AAAI Conference on Artificial Intelligence. Vol. 33. No. 01. 2019.

Page 4

Demonstration

The brief video below walks you through the interface and a few examples.

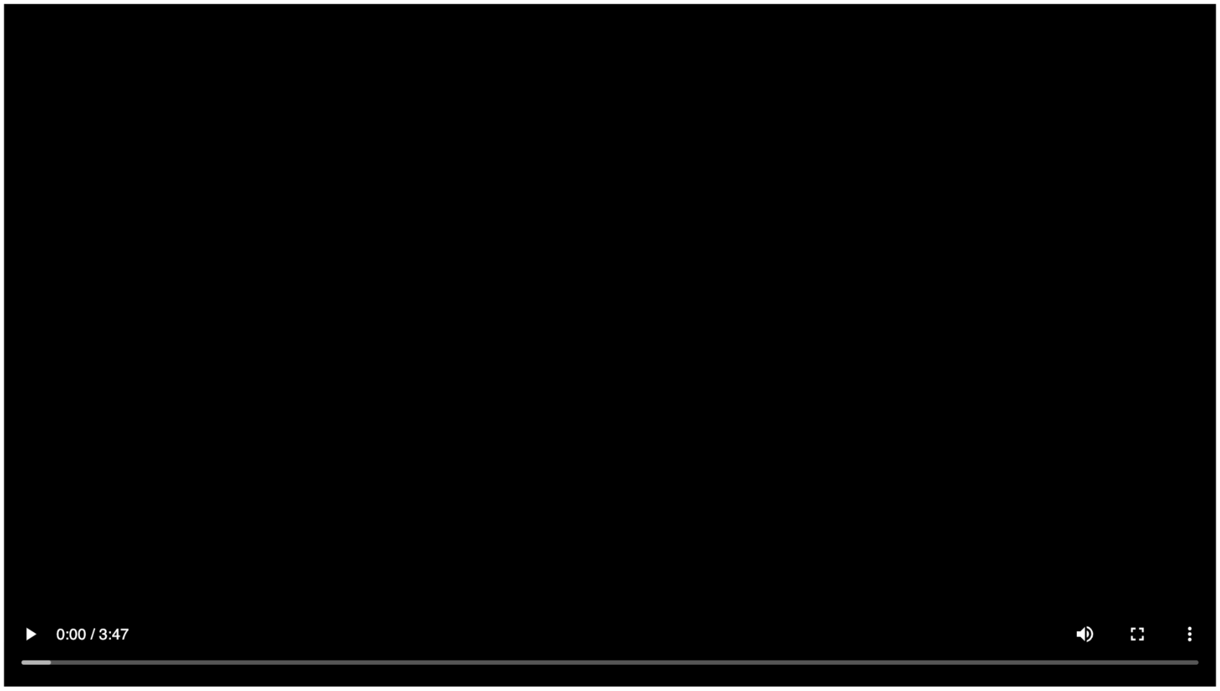

Supplementary Fig. B.4 | Instruction video player. *Screenshot of the instruction video player that participating radiologists used to view the video.*

The instruction video can be found at Supplementary Video | Experiment_Instruction_video.mp4.

Page 5

Consent

You have been asked to participate in a study conducted by researchers from the Massachusetts Institute of Technology (M.I.T.) and Harvard University.

The information below provides a summary of the research. Your participation in this research is voluntary and you can withdraw at any time.

1. Study procedure: We will ask you to examine a number of chest x-rays. We will vary both the amount of information provided about the patient and the availability of an AI support tool.
2. Potential Risks & Benefits: There are no foreseeable risks associated with this study and you will receive no direct benefit from participating

Your participation in this study is completely voluntary and you are free to choose whether to be in it or not. If you choose to be in this study, you may subsequently withdraw from it at any time without penalty or consequences of any kind. The investigator may withdraw you from this research if circumstances arise.

Page 6

Consent

Privacy & Confidentiality

The only people who will know that you are a research subject are members of the research team which might include outside collaborators not affiliated with MIT. No identifiable information about you, or provided by you during the research, will be disclosed to others without your written permission, except: if necessary to protect your rights or welfare, or if required by law. In addition,

your information may be reviewed by authorized MIT representatives to ensure compliance with MIT policies and procedures.

When the results of the research are published or discussed in conferences, no information will be included that would reveal your identity.

Questions
If you have any questions or concerns about the research, please feel free to contact us directly at diagnosticAI@mit.edu.

Your Rights
You are not waiving any legal claims, rights or remedies because of your participation in this research study. If you feel you have been treated unfairly, or you have questions regarding your rights as a research subject, you may contact the Chairman of the Committee on the Use of Humans as Experimental Subjects, M.I.T., Room E25-143B, 77 Massachusetts Ave, Cambridge, MA 02139, phone 1-617-253 6787.

I understand the procedures described above. By clicking next, I am acknowledging my questions have been answered to my satisfaction, and I agree to participate in this study.

Page 7

Interface questions

Before beginning the experiment, we would like to confirm a few facts through the following comprehension questions. Please answer True or False to the following questions.

On this page, participants had access to a "Help" button with the following 3 options:

1. *General Help: "If you encounter any problems, please send an email at DiagnosticAI@mit.edu describing your problem."*
2. *Hierarchy: The pathology hierarchy as presented on page 2.*
3. *Experiment Instructions: Experiment instructions as presented on page 1.*

Participants selected either "True" or "False" to each question through a radio button. They were not able to start the experiment without answering each question correctly. The correct answers are: FFTFTTTT.

1) The algorithm's prediction is based on information from both the X-ray scan as well as the clinical history.

2) When the algorithm does not show a prediction, it is because the algorithm thinks the pathology is not present.

3) The follow up decision refers to any treatment or additional diagnostic procedures that one would conduct based on the findings of the report.

4) Two patients with the same probability score for a condition ought to always receive the same "follow-up" recommendation.

5) When a condition at a higher level of the hierarchy receives a less than ten percent chance of being present then all the lower level conditions within this branch automatically receive a zero probability of being present.

6) If the algorithm says that the probability of a pathology is present with 80% probability, it means that the AI predicts 80 cases out of 100 have the pathology present.

7) Suppose your assessment is that the patient definitely has either edema or consolidation, and you believe that edema is twice as likely as consolidation. Then you would assign 66.67% to edema and 33.33% to consolidation:

8) I should only indicate pathologies and findings that would be relevant in a radiology report for the patient.

Page 8

Bonus Payment

Thank you again for participating in our study. If your responses in this section are close to the average response of an independent group of radiologists for each case, we will give you a $120 gift card to a large e-commerce retailer of your choice (e.g. Amazon, Flipkart). This payment rule is designed so that your chances of winning the prize is highest if you report your best estimate of the probability that the pathology is present. The precise payment rule is available on request, and we will follow up after the experiment if you win the gift card.

Page 9

Practice Images

First, we will present you with 8 patients to practice and familiarize yourself with the interface. In the practice you will see 2 patient cases under each of the possible combinations of AI support and clinical history availability. You will be compensated for these reads even though they are just for practice.

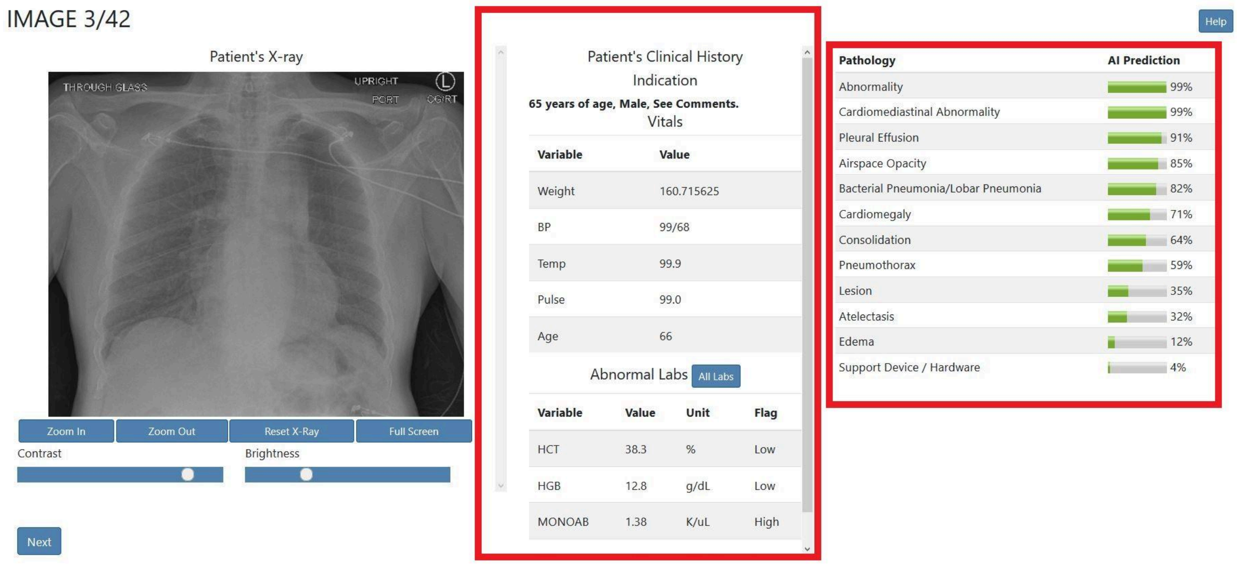

Supplementary Fig. B.5 | Practice patient case preview. *Preview of practice patient cases, with red boxes highlighting the clinical history panel (left) and the AI predictions panel (right).*

Page 10 and onward

Participants were presented with 8 practice patient cases with 2 patient cases under each of the four treatment conditions, followed by patient cases that were considered part of the experiment.

Supplementary Fig. B.6 shows the interface in which clinical history was presented to participants under the relevant treatment conditions. Clicking on "All Labs" would cause a window to pop up that lists all lab values known for the patient, in addition to the abnormal lab values.

Indication

30 years of age, Female, history of hypertension, abnormal EKG, abdominal pain, evaluate for cardiomegaly or mediastinal widening.

Vitals

Variable	Value
Weight	170 lbs
BP	243/166 mmHg
Temp	99.1F
Pulse	99.0 bpm
Age	30

Abnormal Labs All Labs

Variable	Value	Unit	Flag
ALT (SGPT), Ser/Plas	38.0	U/L	High
AST (SGOT), Ser/Plas	39.0	U/L	High
Eosinophil, Absolute	0.01	K/uL	Low

Supplementary Fig. B.6 | Clinical history panel. *The interface in which clinical history was presented to participants. Clinically history includes the indication (top), vitals (middle), and abnormal lab values (bottom).*

The thoroughness of the indication varies across patients. Some examples of varying indications are:

1. *68 years of age, Female, chest pain*
2. *Unknown age, Unknown, Trauma*
3. *55 years of age, Male, Order History: Relevant PMH gastroparesis. Presents with vomiting, retching chest discomfort for a duration of today. Concern for PTX, perforated viscus, pneumomediastinum.*
4. *74 years of age, Female, s/p unwitnessed fall, r/o rib fx, pna or effusion*
5. *Trauma*
6. *56 years of age, Male, S/P ICD/ Pacemaker insertion / Complete X-ray without lifting arms above shoulders.*

Participants used a slider to indicate the probability of a pathology being present, as shown in Supplementary Fig. B.7. When the indicated probability was greater than 10%, the participants were required to indicate the probability for any sub-pathologies and whether a follow-up is recommended. Depending on the pathology, participants were also asked to indicate the size, severity, or position of the pathology.

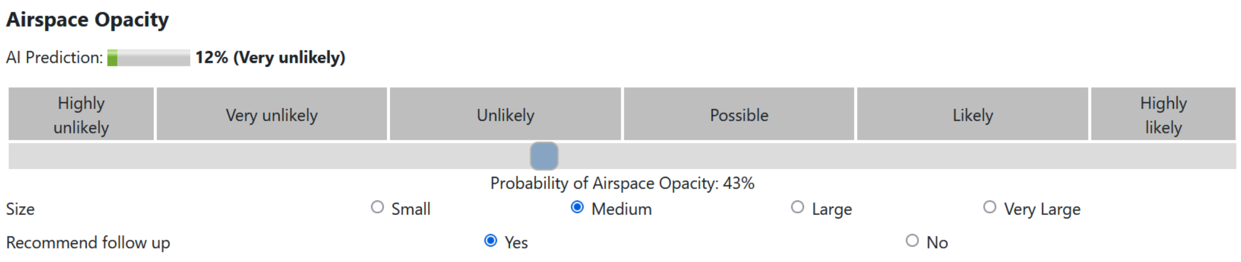

Supplementary Fig. B.7 | Example interface for making a diagnosis. *The interface participants used to indicate the probability of a pathology being present and provide other information such as the recommendation for a follow-up.*

Supplementary References

1. Obuchowski, N. A. & Bullen, J. Multireader Diagnostic Accuracy Imaging Studies: Fundamentals of Design and Analysis. Radiology (2022) doi:10.1148/radiol.211593.
2. Smith, B. J. & Hillis, S. L. Multi-reader multi-case analysis of variance software for diagnostic performance comparison of imaging modalities. *Proceedings of SPIE--the International Society for Optical Engineering 11316*, (2020).
3. Smith, B. J., Hillis, S. L. & Pesce, L. L. *MCMCaov: Multi-Reader Multi-Case Analysis of Variance*. (2023).
4. Agarwal, N., Moehring, A., Rajpurkar, P. & Salz, T. Combining human expertise with artificial intelligence: experimental evidence from radiology. National Bureau of Economic Research. Working paper 31422. https://doi.org/10.3386/w31422 (2023).
