# Supplementary material for: Heterogeneity and predictors of the effects of AI assistance on radiologists
Source: Nat Med. 2024 Mar 19;30(3):837–49. doi: 10.1038/s41591-024-02850-w (PMC10957478; doi:10.1038/s41591-024-02850-w)
Supplement: Supplementary file 2 — Reporting Summary [file 41591_2024_2850_MOESM2_ESM.pdf]

nature portfolio

Corresponding author(s): Pranav Rajpurkar

Last updated by author(s): Jan 7, 2024

Reporting Summary

Nature Portfolio wishes to improve the reproducibility of the work that we publish. This form provides structure for consistency and transparency in reporting. For further information on Nature Portfolio policies, see our Editorial Policies and the Editorial Policy Checklist.

Statistics

For all statistical analyses, confirm that the following items are present in the figure legend, table legend, main text, or Methods section.

n/a	Confirmed	
☐	☒	The exact sample size (*n*) for each experimental group/condition, given as a discrete number and unit of measurement
☐	☒	A statement on whether measurements were taken from distinct samples or whether the same sample was measured repeatedly
☐	☒	The statistical test(s) used AND whether they are one- or two-sided<br>*Only common tests should be described solely by name; describe more complex techniques in the Methods section.*
☐	☒	A description of all covariates tested
☐	☒	A description of any assumptions or corrections, such as tests of normality and adjustment for multiple comparisons
☐	☒	A full description of the statistical parameters including central tendency (e.g. means) or other basic estimates (e.g. regression coefficient) AND variation (e.g. standard deviation) or associated estimates of uncertainty (e.g. confidence intervals)
☐	☒	For null hypothesis testing, the test statistic (e.g. *F*, *t*, *r*) with confidence intervals, effect sizes, degrees of freedom and *P* value noted<br>*Give P values as exact values whenever suitable.*
☐	☒	For Bayesian analysis, information on the choice of priors and Markov chain Monte Carlo settings
☒	☐	For hierarchical and complex designs, identification of the appropriate level for tests and full reporting of outcomes
☒	☐	Estimates of effect sizes (e.g. Cohen's *d*, Pearson's *r*), indicating how they were calculated

Our web collection on statistics for biologists contains articles on many of the points above.

Software and code

Policy information about availability of computer code

Data collection	The experimental interface and system used for data collection are detailed in a separate work: Nikhil Agarwal, Alex Moehring, Pranav Rajpurkar, and Tobias Salz. Combining Human Expertise with Artificial Intelligence: Experimental Evidence from Radiology. (2023). This work does not involve data collection.
Data analysis	Data analysis was conducted using Python 3.9.7 and libraries statsmodels 0.13.5 and scipy 1.10.1; and R 4.1.3 and libraries MRMCaov 0.3.0 and auctestr 1.0.0. Code for the analysis is available at https://https://doi.org/10.5281/zenodo.10467492.

For manuscripts utilizing custom algorithms or software that are central to the research but not yet described in published literature, software must be made available to editors and reviewers. We strongly encourage code deposition in a community repository (e.g. GitHub). See the Nature Portfolio guidelines for submitting code & software for further information.

Data

Policy information about availability of data

All manuscripts must include a data availability statement. This statement should provide the following information, where applicable:

- Accession codes, unique identifiers, or web links for publicly available datasets
- A description of any restrictions on data availability
- For clinical datasets or third party data, please ensure that the statement adheres to our policy

The 324 patient cases from Stanford University’s health care system were used under licensing. They are available at https://stanfordaimi.azurewebsites.net/

datasets/5194008e-61cf-4083-9896-3d4bd8bf8b0b conditioned on the Stanford University Data Research Use Agreement.
The AI predictions used in the experiment were generated by the CheXpert model trained on the CheXpert dataset, which is publicly available.
The clinician-AI collaboration dataset is available at https://osf.io/z7apq/ upon request for access at the OSF dataset page.

Research involving human participants, their data, or biological material

Policy information about studies with human participants or human data. See also policy information about sex, gender (identity/presentation), and sexual orientation and race, ethnicity and racism.

Reporting on sex and gender	The study analyzes data collected from a total of 140 radiologists. Details about the data can be found in the following work: Nikhil Agarwal, Alex Moehring, Pranav Rajpurkar, and Tobias Salz. Combining Human Expertise with Artificial Intelligence: Experimental Evidence from Radiology. (2023).<br>Information about sex and gender of the participating radiologists was collected through the survey question "How do you identify?" with four possible answer options: female (42), male (83), other (1), and "prefer not to answer" (10). These statistics are self-reported. Sex and gender were not considered in the original data collection procedures. No sex- and gender-based analyses have been performed because they require further division of the existing dataset, which decreases statistical power and therefore decreases the strength of analyses that build upon a lack of statistical insignificance.
Reporting on race, ethnicity, or other socially relevant groupings	The study analyzes data collected from a total of 140 radiologists. Details about the data can be found in the following work: Nikhil Agarwal, Alex Moehring, Pranav Rajpurkar, and Tobias Salz. Combining Human Expertise with Artificial Intelligence: Experimental Evidence from Radiology. (2023).
Population characteristics	The study analyzes data collected from a total of 140 radiologists. Details about the data can be found in the following work: Nikhil Agarwal, Alex Moehring, Pranav Rajpurkar, and Tobias Salz. Combining Human Expertise with Artificial Intelligence: Experimental Evidence from Radiology. (2023). There are 136 radiologists with available survey data on covariates: age, years of experience, specialty, and prior experience with AI tools in radiology. The age population characteristics are: The mean age is 37.9 with a standard deviation of 9.8; the age range is [26, 76]. The years of experience population characteristics are: The mean years of experience is 9.6 with a standard deviation of 9.0; the years of experience range is [1, 45]. The specialty population characteristics are: There are 101 out of 136 radiologists who did not specialize in thoracic radiology; there are 35 out of 136 radiologists who specialized in thoracic radiology. The prior experience with AI tools in radiology population characteristics are: There are 63 out of 136 radiologists without prior experience; there are 73 out of 136 radiologists with prior experience.
Recruitment	The study analyzes data collected from a total of 140 radiologists. Details about the data can be found in the following work: Nikhil Agarwal, Alex Moehring, Pranav Rajpurkar, and Tobias Salz. Combining Human Expertise with Artificial Intelligence: Experimental Evidence from Radiology. (2023).
Ethics oversight	The experiment was determined exempt by the MIT Committee on the Use of Humans as Experimental Subjects through exempt determination E-2953. Details about the data can be found in the following work: Nikhil Agarwal, Alex Moehring, Pranav Rajpurkar, and Tobias Salz. Combining Human Expertise with Artificial Intelligence: Experimental Evidence from Radiology. (2023).

Note that full information on the approval of the study protocol must also be provided in the manuscript.

Field-specific reporting

Please select the one below that is the best fit for your research. If you are not sure, read the appropriate sections before making your selection.

☒ Life sciences ☐ Behavioural & social sciences ☐ Ecological, evolutionary & environmental sciences

For a reference copy of the document with all sections, see nature.com/documents/nr-reporting-summary-flat.pdf

Life sciences study design

All studies must disclose on these points even when the disclosure is negative.

Sample size	The study analyzes data collected from a total of 140 radiologists participating in two experiment designs. The non-repeated-measure design included 107 radiologists in an non-repeated-measure design. Each radiologist read 60 patient cases across four subsequences that each contained 15 cases. Each subsequence corresponded to one of four treatment conditions: with AI assistance and clinical histories, with AI assistance and without clinical history, without AI assistance and with clinical histories, and with AI assistance and clinical histories. The four subsequences were organized in a random order and the patient cases were randomly assigned. The repeated-measure design included 33 radiologists in a repeated-measure design. Each radiologist read a total of 60 patient cases, each under each of the four treatment conditions and producing a total of 240 diagnoses. The radiologist completed the experiment in four sessions. In each session, the radiologist read 60 unique patient cases in a sequence of four subsequences that each contained 15 cases, with each subsequence corresponding to one of four treatment conditions. The radiologist read each patient case under a different treatment condition over the four sessions.<br><br>Power calculations were conducted for the data described above and further detailed in the following work: Nikhil Agarwal, Alex Moehring, Pranav Rajpurkar, and Tobias Salz. Combining Human Expertise with Artificial Intelligence: Experimental Evidence from Radiology. (2023). The power calculations were based on a small pilot study that acquired 10 reads in each treatment arm from five radiologists. They simulated draws from the pilot sample for airspace opacity to determine the sample size required to achieve 80% power at 1% significance. Airspace opacity was chosen because it had a reasonable treatment effect of 1.5 percentage points improvement in accuracy in the AI condition during the pilot. They sampled radiologist-patient cases with replacement from the pilot for a grid of potential sample sizes (per treatment arm).

They allocated the N cases to 10 fictitious radiologists. They then estimated the empirical model, regressing the prediction error on radiologist fixed effects and a treatment indicator. Power for a given N is the share of simulations in which they rejected the null of no treatment effect. For each N, they ran 200 simulations. Based on these computations they found that at least 4000 reads in total were needed. The experiment yielded more reads than this baseline.

Data exclusions	In the analysis on experience-based characteristics, 136 out of 140 radiologists provided survey data on experience-based characteristics. The 4 radiologists who did not provide these data were excluded from the analysis. All available data were used in other analyses in the study.
Replication	All randomization involved in the analyses in the study, such as through random sample selection during split sampling, had controlled seeds to ensure reproducibility. All attempts at executing the code led to the same outputs. During development and verification of the results introduced in this work, the same analyses were run at least 5 times and all yielded the same results with the same seeds.
Randomization	Allocation into with-AI and without-AI treatment groups was random. Radiologists were randomly assigned a subset of patient cases to read, and the treatment conditions under which the cases were shown were also selected at random.
Blinding	The investigators were blinded to the with-AI and without-AI treatment group allocation during data collection/analysis.

Reporting for specific materials, systems and methods

We require information from authors about some types of materials, experimental systems and methods used in many studies. Here, indicate whether each material, system or method listed is relevant to your study. If you are not sure if a list item applies to your research, read the appropriate section before selecting a response.

Materials & experimental systems

n/a	Involved in the study	
☒	☐	Antibodies
☒	☐	Eukaryotic cell lines
☒	☐	Palaeontology and archaeology
☒	☐	Animals and other organisms
☒	☐	Clinical data
☒	☐	Dual use research of concern
☒	☐	Plants

Methods

n/a	Involved in the study	
☒	☐	ChIP-seq
☒	☐	Flow cytometry
☒	☐	MRI-based neuroimaging
